# Supplementary material for: Refining clinical algorithms for a neonatal digital platform for low-income countries: a modified Delphi technique
Source: BMJ Open. 2021 May 18;11(5):e042124. doi: 10.1136/bmjopen-2020-042124 (PMC8130744; doi:10.1136/bmjopen-2020-042124)
Supplement: Supplementary data [file bmjopen-2020-042124supp001.pdf]

Round one questionnaire

NeoTree questions on algorithms for expert review

Prior to each of these algorithms the user of the NeoTree must complete the emergency triage section. This section ensures that the baby is resuscitated as per Helping Babies Breathe, guiding the user to check for danger signs. Any abnormal parameters entered trigger immediate prompting to give appropriate resuscitation before addressing these diagnoses. Included in this will be a blood sugar measurement.

Section 1. Neonatal Sepsis

Summary of sepsis algorithm

For this part of the algorithm please assume you have the following equipment/resources:

- the NeoTree application on a tablet device
- a pulse oximeter
- a thermometer
- a clock
- a stethoscope
- oxygen
- bubble CPAP

Please assume, however, that you are working in an environment where there is no availability for blood cultures, inflammatory markers, white cell count, or chest x-ray. Lumbar puncture is available for gram stain, protein and cell count only. In the future these investigations may become more widely available in newborn facilities, and there is the potential to add these extra parameters to the NeoTree algorithm. But currently, when we say ‘sepsis’ we mean presumed sepsis that cannot be confirmed with laboratory investigations.

Within the NeoTree algorithm, the diagnosis of sepsis is divided into 4 categories according to the presence or absence of signs/symptoms and/or risk factors and the age of the newborn (Flowchart 1). In order to account for different levels of risk we have weighted both signs/symptoms and risk factors as ‘MAJOR’ or ‘MINOR’ according to COIN / WHO / best judgement. A MAJOR sign/symptom is weighted twice as heavily as a MINOR one, so that, for example, in the diagnosis of symptomatic sepsis either 1 MAJOR sign/symptom or 2 MINOR signs/symptoms will trigger the diagnosis. For asymptomatic sepsis only, risk factors are required. We have used a similar weighting system for risk factors.

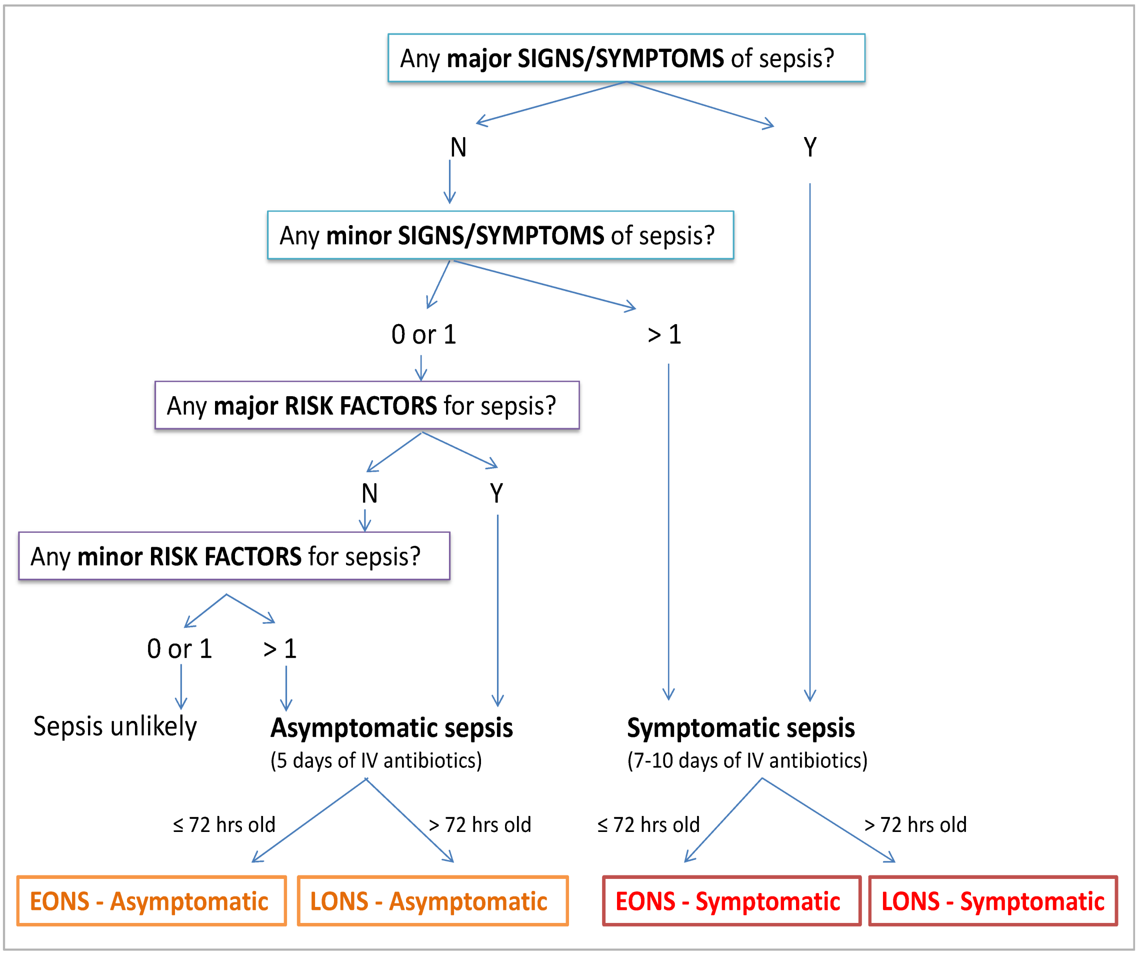

EONS = Early onset neonatal sepsis, LONS = Late onset neonatal sepsis

Flowchart 1: Categories of sepsis are divided by presence (symptomatic) or absence (asymptomatic) of signs/symptoms and by age of newborn

Table 1: Risk factors and signs/symptoms of sepsis	
**MAJOR risk factors**	**MINOR risk factors**
1. Maternal fever >38°C in labour *+<br>2. Offensive liquor*+<br>3. Prolonged rupture of membranes (PROM) >18 hrs*+<br>4. Prolonged second stage (>3 hrs of active pushing)*<br>5. Very / extremely premature (<32/40 weeks gestation)~	1. Born before arrival (BBA)~<br>2. Prematurity (32-36/40 weeks gestation) (N.B in the app you can only round to the closest week i.e 36 or 37 weeks)~
**MAJOR signs/symptoms**	**MINOR Signs/symptoms**
1. Temperature >37.5°C*<br>2. Pustules all over body*+<br>3. Boil/abscess*+<br>4. Red skin all around umbilicus*+<br>5. Bulging fontanelle*+<br>6. Grunting or severe respiratory distress/mod-severe work of breathing*+<br>7. History of apnoea~<br>8. Jaundice <24 hrs old+<br>9. Tachypnoea >60*+ (except 60-80 bpm at <2hrs old)~<br>10. Lethargy+<br>11. Convulsions+<br>12. Bilious vomiting~	1. Mild work of breathing~<br>2. Tachycardia (HR>160) that can't be explained by fever or crying~<br>3. Tachypnoea 60-80 bpm*+ at <2hrs old~<br>4. Pallor+<br>5. Abdominal distension~<br>6. Poor feeding/vomiting+<br>7. Weak or absent suck (AND gestation >34 weeks)~<br>8. Irritability*+

bpm = breaths per minute, HR = heart rate

Notes: *Source/Evidence used: *COIN, +WHO, ~NeoTree team best judgement*

Consider Meningitis:

For the following scenarios the NeoTree will ask the health care worker (HCW) to consider meningitis:

- Temp >37.5°C* AND any of
  - Bulging Fontanelle*+
  - Hypertonia+
  - Persistent irritability*+
  - Lethargy*+

OR

- Convulsions*+ / History of convulsions*
- Hydrocephalus (obviously enlarged head or OFC >37cm)~

Notes: *Source/Evidence used: *COIN, +WHO, ~NeoTree team best judgement*

Explanatory notes for Sepsis algorithm:

- Regarding work of breathing, for the sake of simplicity the NeoTree algorithm asks the user to observe the work of breathing and rate it as mild, moderate or severe with the aid of educational videos on the app. There is the potential to include a validated respiratory distress score in future iterations.

- Hypothermia has not been included as a sign of sepsis due to the high rates of environmental hypothermia on admission in these settings. Persistent hypothermia despite warming may be included in future iterations.

- We have separated mild tachypnoea of 60-80 in ages <2 hours into the minor signs/symptoms so that if it exists without any other signs/symptoms or risk factors it can have the diagnosis of transient tachypnoea of the newborn rather than sepsis.

Sepsis diagnosis questions:

Table 2: Sepsis diagnosis questions

**Question**	**Disagree → Agree**	**Discuss responses**
**1. Do you agree with the following MAJOR risk factors?**		
a. Maternal fever >38 °C in labour<br>b. Offensive liquor<br>c. PROM >18 hours<br>d. Prolonged second stage (>3 hours of active pushing)<br>e. Very / extremely premature (<32/40 weeks gestation)	1 2 3 4 5<br>1 2 3 4 5<br>1 2 3 4 5<br>1 2 3 4 5<br>1 2 3 4 5	
**2. Do you agree with the following MINOR risk factors?**		
a. Born before arrival (BBA)<br>b. Prematurity (32-36/40 weeks gestation)	1 2 3 4 5<br>1 2 3 4 5	
**3. Do you agree with the following MAJOR signs/symptoms?**		
a. Temperature >37.5 °C<br>b. Admitted with or history of fever<br>c. Pustules all over body<br>d. Boil/abscess<br>e. Red skin all around umbilicus<br>f. Bulging fontanelle<br>g. Grunting or severe respiratory distress or mod-severe WOB<br>h. History of apnoea<br>i. Jaundice <24 hours old<br>j. Tachypnoea >60 bpm **(except 60-80 & < 2 hours old)**<br>k. Lethargy<br>l. Convulsions<br>m. Bilious vomiting	1 2 3 4 5<br>1 2 3 4 5<br>1 2 3 4 5<br>1 2 3 4 5<br>1 2 3 4 5<br>1 2 3 4 5<br>1 2 3 4 5<br>1 2 3 4 5<br>1 2 3 4 5<br>1 2 3 4 5<br>1 2 3 4 5<br>1 2 3 4 5<br>1 2 3 4 5<br>1 2 3 4 5	
**4. Do you agree with the following MINOR signs/symptoms?**		
a. Mild work of breathing<br>b. Tachycardia that can't be explained by fever/crying<br>c. Pallor<br>d. Distended abdomen<br>e. Poor feeding/vomiting<br>f. Weak or absent suck (AND gestation >34 weeks)<br>g. Irritability<br>h. Tachypnoea 60-80 bpm & <2 hrs old	1 2 3 4 5<br>1 2 3 4 5<br>1 2 3 4 5<br>1 2 3 4 5<br>1 2 3 4 5<br>1 2 3 4 5<br>1 2 3 4 5<br>1 2 3 4 5	
**5. Are there any additional risk factors, signs or symptoms that you think should be included for a low-income setting? For example…**		

a. Hypothermia <35.5 °C<br>b. Joint swelling (NB NeoTree is more immediate mx).<br>c. Reduced movement of limbs	Y/N, if yes Maj/Minor<br>Y/N, if yes Maj/Minor<br>Y/N, if yes Maj/Minor	
**6. If you have not already stated. Should any of the risk factors or signs and symptoms be MAJOR or MINOR?**		
**7. Do you agree with the cut off from 72 hours for early and late neonatal sepsis?**	1 2 3 4 5	
**8. Do you agree with the definition of maternal fever being >38 degrees °C?**	1 2 3 4 5	
**9. Should PROM be >18 hours or >24 hours in this setting?**	18 hours = 1<br>24 hours = 5	
**10. Do you agree that fever in a newborn should be classified as >37.5 °C in this setting?**	1 2 3 4 5	
**11. Do you agree with the cut- off at 34 weeks gestation for the 'no suck' in a non-prem sepsis sign?**	1 2 3 4 5	
**12. Please comment on our weighting system of Major = 100%, Minor = 50%**		
**13. Please comment on the criteria for 'Consider meningitis'**		

Management of Sepsis:

Box 1. Management page for Sepsis in NeoTree:

IV access
Get IV access
If possible, perform sepsis screen
Sepsis screen: FBC, CRP, Blood Culture, +/- Lumbar Puncture
(CRP is C-reactive protein a marker of infection available in some labs)

Antibiotics
Start antibiotics according to local guidelines. If no guidelines give:
-Penicillin 50,000 iu/kg 12 hourly IV/IM and
-Gentamycin 3 or 5 mg/kg/dose 24 hourly IV/IM (LBW or Term)
Length of antibiotics treatment:
a) Asymptomatic sepsis, treat for 5 days
b) Symptomatic sepsis, treat for 7-10 days
Frequency of penicillin is 6 hourly if >7 days age
Use Flucloxacillin and Gentamycin if skin infection or pustules

Supportive care
Thermoregulation: aim for temperature between 36.5 - 37.5 °C
If baby has fever remove from warmer and unwrap
Provide oxygen or CPAP as needed according to TRY CPAP algorithm
Check blood glucose and provide feeding support as needed

- Consider 10ml/kg fluid bolus if shocked as per SHOCK algorithm
- Consider 2.5mls/kg 10% dextrose if BS <2.5 mmol or 45 mg/dl – as per HYPOGLYCAEMIA algorithm

*Source/Evidence used: *COIN*

- The NeoTree antibiotic management guidelines for sepsis follows the Malawi COIN guidelines. The NeoTree recommends 5 days of IV antibiotics for asymptomatic sepsis and 7-10 days for symptomatic sepsis.

Sepsis management questions:

Table 3. Sepsis management questions		
**Question**	**Disagree → Agree**	**Discuss responses**
**1. Do you agree with the specified sepsis investigations if possible?**	1 2 3 4 5	
**2. Do you agree with the antibiotic choices and doses if no local recommendation?**	1 2 3 4 5	
**3. Do you agree with antibiotic duration?**<br>a) Asymptomatic sepsis = 5 days<br>b) Symptomatic sepsis = 7-10 days	 1 2 3 4 5<br>1 2 3 4 5	

Section 2. Birth Asphyxia

Summary of Birth Asphyxia algorithm:

We have divided the diagnosis of birth asphyxia into 2 categories according to the presence or absence of risk factors or signs/symptoms in Table 4:

A. ***Consider birth asphyxia*** = 1 risk factor AND 1 sign/symptom of birth asphyxia
B. ***Birth asphyxia*** = either
   2 or more risk factors AND 1 sign/symptom OR
   No known risk factors AND 2 or more signs/symptoms

Table 4. Risk factors, signs and symptoms for birth asphyxia

**Risk factors**	**Signs/Symptoms**
• Foetal distress*<br>• Prolonged second stage*<br>• Delivery = Vacuum / EmCS / Breech~<br>• Apgar at 5 mins <7+<br>• Resuscitation: BVM for >5 mins* / CPR* / resuscitated for >10 mins~<br>• Birth injury~	• Absent suck AND >34/40 gestation~<br>• Hypotonia AND >34/40 gestation~<br>• Irritable*+<br>• Lethargy*+<br>• Coma*+<br>• Convulsions*+

EmCS = Emergency caesarian section, BVM = Bag valve mask ventilation, CPR = Cardio pulmonary resuscitation, BW = birth weight.

Evidence/Source used: *COIN, +WHO, ~NeoTree team best judgement

Birth asphyxia diagnosis questions:

Table 6: Birth asphyxia diagnosis questions

Questions	Disagree → Agree	Discuss responses
**1. Do you agree with the diagnostic criteria for:**		
A. 'Consider birth asphyxia'<br>B. 'Birth asphyxia'	1 2 3 4 5<br>1 2 3 4 5	
**2. Do you agree with the following risk factors for birth asphyxia?**		
a. Foetal distress<br>b. Prolonged second stage >3 hours duration<br>c. Delivery = Vacuum / EmCS / breech<br>d. Apgar at 5 mins <7<br>e. Resuscitation: BVM >5 mins / CPR lasted >10 mins<br>f. Birth injury	1 2 3 4 5<br>1 2 3 4 5<br>1 2 3 4 5<br>1 2 3 4 5<br>1 2 3 4 5<br>1 2 3 4 5	
**3. Do you agree with the following signs/symptoms for birth asphyxia?**		
a. Absent suck and gestation >34/40<br>b. Hypotonia and gestation >34/40<br>c. Irritable<br>d. Lethargy<br>e. Coma<br>f. Convulsions	1 2 3 4 5<br>1 2 3 4 5<br>1 2 3 4 5<br>1 2 3 4 5<br>1 2 3 4 5<br>1 2 3 4 5	
4. Should birth asphyxia be classified as mild, moderate or severe?	1 2 3 4 5	
**5. Do you think the following sign/symptoms and risk factors should be included?**		
a. Poor feeding<br>b. Respiratory distress<br>c. >4kg	Y/N<br>Y/N<br>Y/N	
6. Are there any other risk factors or signs/symptoms to be added/removed?		
7. Due to the difficulties of training HCW in checking safely for the Moro reflex in these settings we have removed from the assessment. Do you agree?	1 2 3 4 5	

Box 2: Management page for Birth Asphyxia in NeoTree:

1. **Keep warm**
   KMC/radiant warmer

   (Unless the patient is hyperthermic)

2. **Respiratory support**
   Put on appropriate respiratory support

3. **Feeding support, anticonvulsants and antibiotics**
   Mild/Moderate:
   - Give NG feeds- most will have a poor suck reflex
   - Mother can still offer breast to develop the suck reflex
   - Check blood sugar (BS) and give 10% dextrose bolus if BS <2.5mmol/L or <45mg/dL

   Severe: where NG feeds are not tolerated, consider IV fluids.

If evidence of seizure activity give **PHENOBARIBITONE** as per convulsions page.

Reassess suck, tone, coma, seizures every 8 hours for 72 hours in hospital

Treat for sepsis with antibiotics

Convulsions page in NeoTree:

- Load phenobarbitone 20 mg/kg
- If seizures ongoing can reload twice
- Start 5 mg/kg maintenance (24 hours after loading dose)
- Stop phenobarbitone at 72 hours if no further seizures and neurologically normal

*Source/Evidence used: *COIN*

Management of Birth Asphyxia questions:

**Table 7. Management of Birth Asphyxia questions**		
**Questions**	**Disagree → Agree**	**Discuss responses**
Do you agree not to do passive cooling for infants in low resource settings?	1 2 3 4 5	
Should the newborn be given IV fluids if not tolerating oral or NG feeds?	1 2 3 4 5	

Section 3. Respiratory distress of the newborn (RDN)

Summary of the RDN algorithm:

For this part of the algorithm please assume you have the following equipment/resources:

- the NeoTree application on a tablet device
- a pulse oximeter
- a thermometer
- a clock
- a stethoscope
- oxygen
- bubble CPAP

Please assume, however that you do not have chest x-ray available to you. We have divided this diagnosis into the following 4 categories:

1. Meconium aspiration

2. Respiratory Distress Syndrome (RDS)

3. Congenital pneumonia

4. Transient Tachypnoea of Newborn

Flowchart 2: RDN diagnostic algorithm

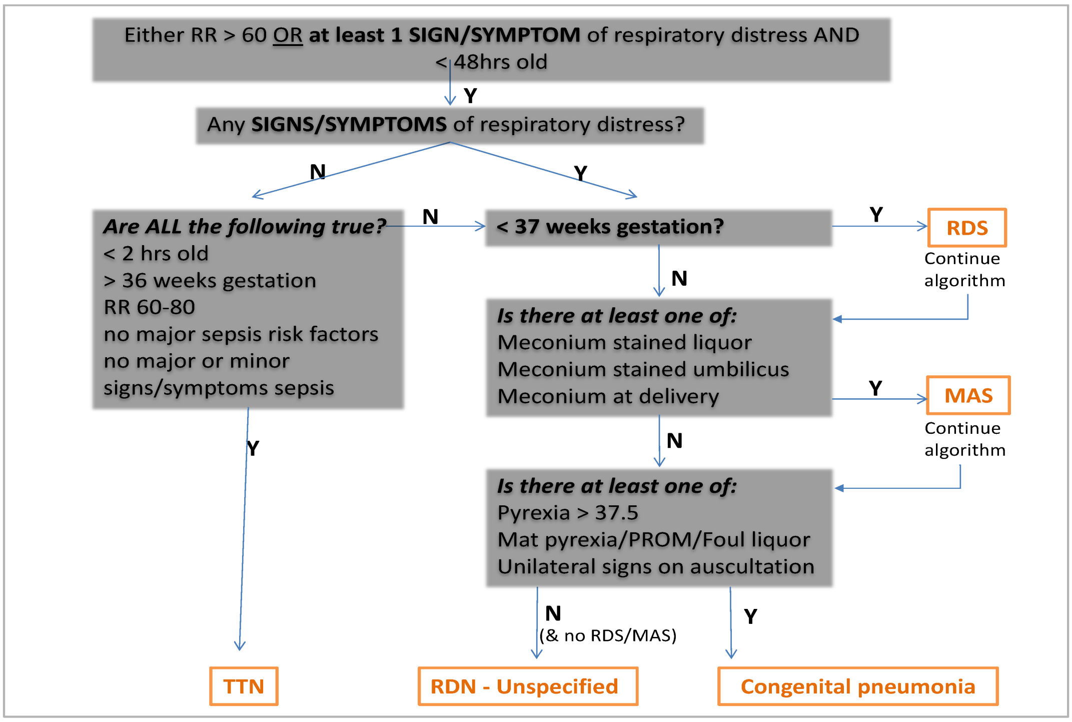

MAS = Meconium aspiration, PROM = prolonged rupture of membranes, RDN = Respiratory Distress of Newborn, RDS = Respiratory distress syndrome, RR = Respiratory rate, TTN = Transient Tachypnoea of Newborn, Y= Yes, N = No.

Table 8. Signs/ Symptoms for RDN
• Grunting or severe recessions or moderate-severe work of breathing*<br>• Blue~ / cyanotic*<br>• Apnoea~<br>• History of fast or laboured breathing+<br>• History of noisy breathing~<br>• Saturations in air or oxygen <90%~<br>• Bilateral wheeze or crackles on auscultation+<br>• History of cough (only included in algorithm for congenital pneumonia)~

Source/Evidence used: *COIN, +WHO, ~NeoTree team best judgement

RDN diagnosis questions:

Table 9: RDN diagnostic algorithm questions		
**Questions**	**Disagree → Agree**	**Discuss responses**
1. Do you agree that on admission a 'history' of fast or laboured breathing or noisy breathing is relevant as a sign or symptom of RDN when not present on admission?	1 2 3 4 5	
2. Do you agree that tachypnoea of 60 - 80bpm <2 hours old without signs or symptoms of sepsis should be treated as TTN, and no antibiotics given?	1 2 3 4 5	
3. Do you agree with tachypnoea of >60 for the other categories of RDN	1 2 3 4 5	
4. Are there any other signs/symptoms to be removed or to be considered?		
5. Do you agree with the diagnostic criteria for:<br>a) MAS?<br>b) RDS?<br>c) TTN?<br>d) Congenital pneumonia?	 1 2 3 4 5<br>1 2 3 4 5<br>1 2 3 4 5<br>1 2 3 4 5	
6. Are there any other risk factors to be considered? For example: Diabetic mother for MAS and RDS		

RDN Management:

We have presented the management for all 4 types of Respiratory distress of the newborn in one management page; however, in reality they each get an individual page in the app. NB. RDN – unspecified would be treated as per the 'GENERIC' section.

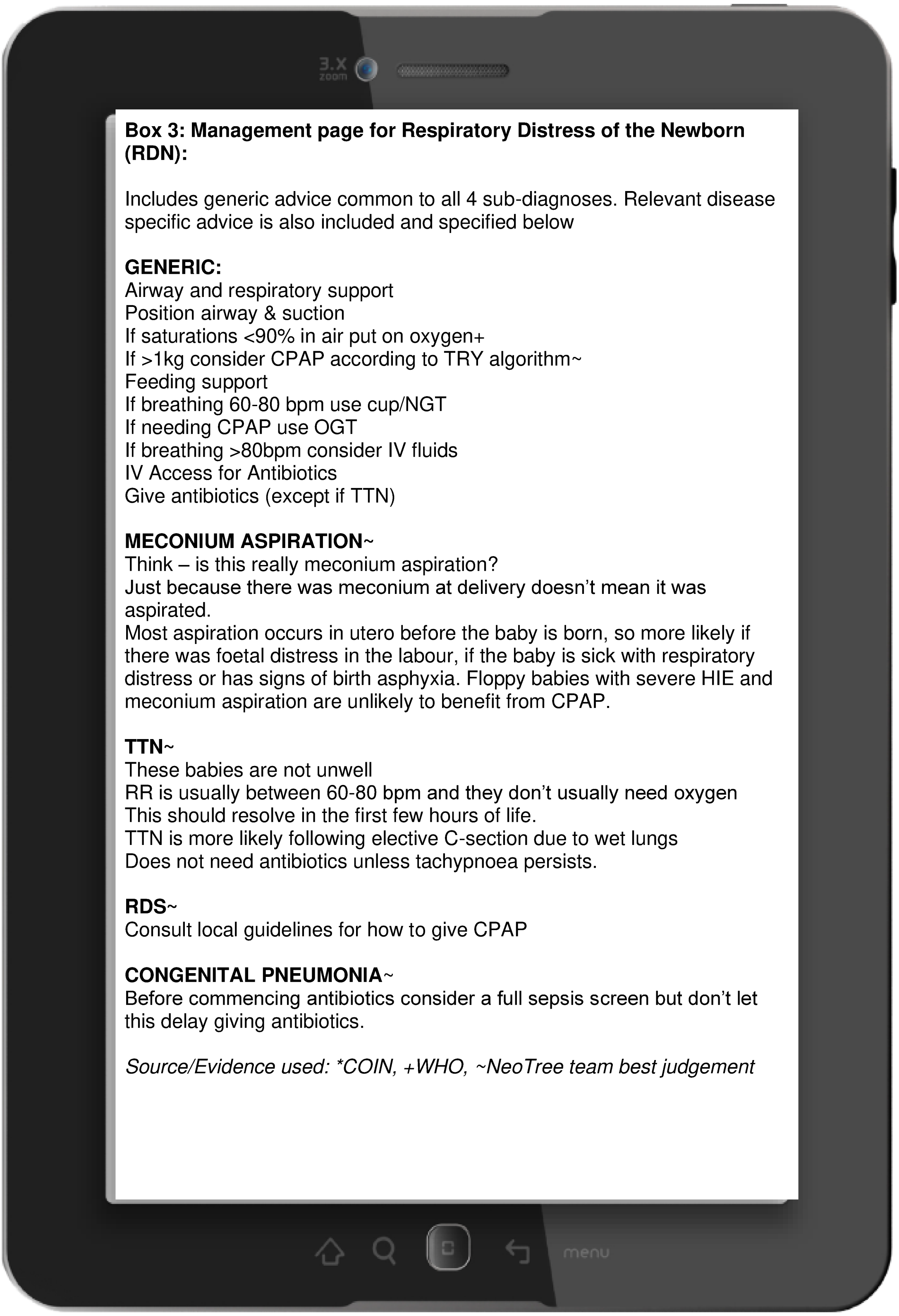

Box 3: Management page for Respiratory Distress of the Newborn (RDN):

Includes generic advice common to all 4 sub-diagnoses. Relevant disease specific advice is also included and specified below

GENERIC:
Airway and respiratory support
Position airway & suction
If saturations <90% in air put on oxygen+
If >1kg consider CPAP according to TRY algorithm~
Feeding support
If breathing 60-80 bpm use cup/NGT
If needing CPAP use OGT
If breathing >80bpm consider IV fluids
IV Access for Antibiotics
Give antibiotics (except if TTN)

MECONIUM ASPIRATION~
Think – is this really meconium aspiration?
Just because there was meconium at delivery doesn't mean it was aspirated.
Most aspiration occurs in utero before the baby is born, so more likely if there was foetal distress in the labour, if the baby is sick with respiratory distress or has signs of birth asphyxia. Floppy babies with severe HIE and meconium aspiration are unlikely to benefit from CPAP.

TTN~
These babies are not unwell
RR is usually between 60-80 bpm and they don't usually need oxygen
This should resolve in the first few hours of life.
TTN is more likely following elective C-section due to wet lungs
Does not need antibiotics unless tachypnoea persists.

RDS~
Consult local guidelines for how to give CPAP

CONGENITAL PNEUMONIA~
Before commencing antibiotics consider a full sepsis screen but don't let this delay giving antibiotics.

*Source/Evidence used: *COIN, +WHO, ~NeoTree team best judgement*

Management of RDN questions:

Table 10: Management of RDN questions		
**Questions:**	**Disagree → Agree**	**Discuss responses**
1. Do you agree with a cut off of 90% oxygen saturations before giving oxygen?	1 2 3 4 5	
2. Do you agree to give antibiotics in all cases except TTN?	1 2 3 4 5	
3. Do you agree with the time cut-off of at 2 hours for TTN? Would you have a higher or lower threshold?	1 2 3 4 5	
4. If it is possible to perform a CXR when and how would you recommend?		
5. Please give any other comments or opinions of the above.		

Section 4. Hypothermia

Box 4: Hypothermia management page in NeoTree:

Mild = 36 - 36.4°C+*

Mod = 32 - 35.9°C+*

Severe = <32°C+*

1. **Warm the baby**
   Mild: Skin to skin (KMC position)+*
   Moderate /severe: place on warmer/resuscitaire~

2. **Limit heat loss**
   Make sure the baby is dry+*
   Put on a hat and wrap up the baby+*
   If on the resuscitaire put the sides up~
   Ensure room is free from drafts- windows and doors closed+*
   The 4 ways by which a baby can lose heat are:+*

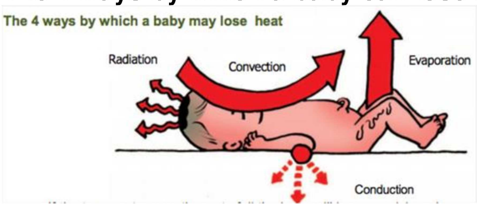

3. **Monitor**
   Measure temperature every 15-30 minutes~
   Watch out for overwarming+*
   Complete NeoTree assessment to assess for signs of infection, apnoea and hypoglycaemia*

Evidence/Source: *COIN, +WHO, ~NeoTree team best judgement

Hypothermia questions:

We have not included an algorithm for hypothermia here, as it is simply the diagnostic criteria stated at the top of the thermoregulation management page.

**Table 11: Hypothermia questions**		
**Questions**	Disagree → Agree	Discuss responses
1. Do you agree with the diagnostic criteria for hypothermia?	1 2 3 4 5	
2. Should we add more detail on ongoing management?	1 2 3 4 5	
3. Any other comments?	1 2 3 4 5	

References

http://www.who.int/maternal_child_adolescent/documents/child_hospital_care/en/WHO. 2013. Pocket book of hospital care for children: Guidelines for the management of common childhood illnesses

cms.medcol.mw/cms_uploaded_resources/41905_12.pdf
Ministry of Health. 2017. Care of the infant and newborn in Malawi The COIN Course Participants Manual.

Round two questionnaire

For items that did not reach a consensus (<80% agreement) the questions have been:

1. Removed
2. Kept because part of WHO or Malawian neonatal (COIN) guidelines
3. Changed and submitted below for a second round

For items with a consensus (>80% agreement) we have kept or changed to WHO wording on expert advice.

Please score the new items using the Likert scale of 1 (strongly disagree) to 5 (strongly agree) and provide comments for those items that you disagree with. Please read expert responses from round 1 for each item to aid your response for the second round.

Section 1. Neonatal Sepsis

Sepsis diagnosis questions

We did not reach a consensus on our major and minor weighting system approach to diagnosing neonatal sepsis, and we cannot come to a decision until we have done our clinical validation study and collected data from Zimbabwe study using blood cultures to identify which risk factors are most strongly predictive of sepsis. However, we would still like to explore the usefulness for 'other' risk factors to be included alongside the WHO and COIN danger signs.

1. Do you agree with the following 'other' risk factors for neonatal sepsis?

A. Born before arrival (BBA)

Expert responses

I would never consider this a risk factor in a high resource setting for sepsis but in a low resource setting where you have no clue where or how the baby was born, what equipment was used and what the circumstances where I can see this as a risk factor.

I don't know this as a risk factor. I hesitate because there is so much controversy with that statement and we had a lot of discussion about this in Rwanda.

I guess there is then uncertainty with all the maternal risk factors. So yes, I agree but I don't think there is any evidence behind it but I can see the logic.

It depends where. Yes, is they were born at home and un-booked mothers who have not had had antenatal care but not if born in another facility.

Depends on where they were born and who delivered/cut the cord

It depends where the baby has been born. Quite a lot of babies are born outside the hospital and that would be mean every baby would need antibiotics.

I disagree. Is this to the hospital? I will have to disagree here because depending on where you live most babies do not even come to the hospital.

Traditionally this is what people have done but I don't get very excited about it and I advise to follow the WHO guidelines.

Original question	% agreement	Action taken	Comments
A. Born before arrival (BBA)	64%	Change for COIN<br><br>Remove for international use	▪ BBA means that the baby was born in a vehicle (car, ambulance etc) or the roadside in Malawi which are considered dirty environments.<br>▪ Not included in WHO or COIN guidelines
**New proposed question**	**Indicate level of agreement**		**Your thoughts/comments**
Babies born en route to the hospital	1 2 3 4 5		

B. Prematurity (32-36/40 weeks gestation)

Expert responses

If the baby was delivered as a clean cold caesarean section for maternal reasons and the mother was not in labour then antibiotics should not be given. However, if the baby was born spontaneously premature then they should be given antibiotics

There was a study done recently in Tanzania, Professor Lindberg. Initially they had everyone on antibiotics and then they excluded the mothers who had caesarean sections for maternal reasons. They spoke about antibiotics being given to the mother and the fetus for 48 hours.

Gestation should also be given as weight.

Usually gestation is reported in months not weeks.

Gestation is often unknown. Every baby was treated as sepsis in Zambia. Every baby had a cannula on the neonatal unit which was a risk of infection alone. Too many babies were getting antibiotics. Every mother who had a GA their babies was admitted to the neonatal unit, a cannula sited and antibiotics were given. There was no thought process at all.

Gestation is not very useful, I only ever see 28 weeks, 32 weeks or term documented. Weight and gestation are more useful < 1.5 kg preterm, < 1kg very preterm. 1.5-2 kg could be growth restriction.

Gestation should be in combination with weight as they often don't know the gestation.

It is nice to have weight and gestation. Often prematurity is not recognised as it should be so weight is useful to highlight prematurity

You are better off using weight for gestation.

The simpler you make things the more effective they are. You are replying on ballard score which has +/- 2 weeks accuracy. So, a 31 weeker could fit into either category. I think you should just have prematurity just as a major risk factor.

Again, weight rather than gestation and low birth weight baby as a risk factor instead of prematurity and make it a major risk factor.

Agree for spontaneous but not for maternal reasons. Need to have weight and gestation/Ballard.

You need to have weight as well but they may be growth restricted and not premature but it is probably not a bad idea to have both premature and IUGR babies on antibiotics.

Add weight because sometimes the gestation is not known

I guess this is ok for one minor risk factor but I would also include weight to guide gestation.

I think my general comment would be the danger signs for WHO and the danger signs for essential care for every baby and essential care for small babies are the same. Those are probably the ones I would use to treat with antibiotics and not to treat with antibiotics.

Again, use weight. The weight is more useful than gestation. Being SGA is probably more important than premature.

**Original question**	**% agreement**	**Action taken**	**Comments**
B. Prematurity	71%	Change to include weight for gestation	▪ The application developers are trying to distinguish whether you are concerned with all premature babies being at risk of infection or just extreme (< 32/40) or slightly (32-26 weeks) preterm<br>▪ Not included in WHO or COIN guidelines
**New proposed question**	**Indicate level of agreement**		**Your thoughts/ comments**
<32/40 weeks gestation or <1500g	1 2 3 4 5		
32-36/40 weeks gestation or 1500-2500g	1 2 3 4 5		

C. Admitted with or history of fever – this question was originally asked as a MAJOR (DANGER) sign/symptom

Expert responses

I disagree. It needs to be repeated

It should be a minor risk factor

I agree. I would take it seriously if a mother reported that the baby had a fever because that is very unusual.

You need to say two temperatures or persistent.

There are so many mothers who over wrap their infants

I would monitor the baby. It should be a minor risk factor

I disagree. This depends on whether the baby is going be admitted for observation or not. Actually, I would read the reading myself because the rate of poor thermometers readings in developed countries is really high let alone in undeveloped countries so they would give every baby antibiotic. I am biased because I disagree with some of the WHOs stance on even the danger signs. I think they are too general, too many kids get antibiotics that do not need to. If you look at work done published in Rwanda, Kosovo and other countries, it's just that the antibiotic resistance rate is so high and kids are getting treatment that is unnecessary. So, I am going to lean probably most towards non-treatment than treatment.

I agree if this is the WHO or local guideline

**Original question**	**% agreement**	**Action taken**	**Comments**
C. Neonate admitted with or history of fever	57% agreement as a MAJOR risk factor	Change to 'other' risk factor and change wording	
**New proposed question**	**Indicate level of agreement**		**Your thoughts/comments**
Mother reports a non-measured fever	1 2 3 4 5		

2. Do you agree that the following is a DANGER/MAJOR sign or symptoms for neonatal sepsis?

Bilious vomiting

It could be a surgical reason and not sepsis.

In low resource settings yes we would start antibiotics

Definitely

Not alone.

It is a problem but I would not jump to sepsis.

It is a minor risk factor and it needs to be persistent to be major.

You are going to be over treating here because it could just be a congenital abnormality. It would be a minor risk factor.

It should be a minor risk factor and not stand alone.

That's not a danger sign but I agree that it's worth antibiotics but very rarely infectious. This kid is going to have one or two things wrong with it. The most likely thing is going to be a general obstruction. These babies in Africa are going to die. In a premature baby it is going to be NEC and giving that baby antibiotics might be helpful. It's controversial.

Not in isolation. It should be a minor risk factor with abdominal distension

Distended abdomen

This is major risk factor in low resource settings as it usually frank fulminant NEC even with bigger babies. Surprised how common it was.

We do have to take it seriously. It is relatively subjective especially with preterm babies whose bellies always look big and round. I feel this needs 3 minors.

You again need to look for other reasons first. NEC is not as common in LIC.

If persistent

Very vague.

I disagree, you can get it from crying

This should be in combination with bilious vomiting and then this would be a major.

**Original question**	**% agreement**	**Action taken**	**Comments**
Bilious vomiting	50%	Change to combine with severe abdominal distension.	▪ Severe abdominal distension is a WHO sign of serious bacterial infection
**New proposed question**	**Indicate level of agreement**	**Please highlight whether it should be an 'other' risk factor or major (danger) sign**	**Your thoughts/comments**
Bilious vomiting with severe abdominal distension	1 2 3 4 5	Other or Major	

3. Do you agree with the following 'other' signs/symptoms of neonatal sepsis?

Irritability [*We acknowledge the comments suggesting the inclusion of "high pitched cry" but this is scored in a separate, meningitis algorithm*]

Expert responses

It is vague. More description is needed i.e. Inconsolable baby with an abnormal high-pitched cry.

It is not a sign of sepsis per say

This is subjective and needs to be defined further. A true irritable inconsolable baby I would agree. It needs videos.

I would probably do irritability and high-pitched cry

It could be something else.

This should be a major risk factor because the child could have meningitis

I disagree. Almost every baby in the 8-12 weeks of age is the most irritable thing in the world so I don't think so.

I would add lethargy and make this a major risk factor. I would try and simplify and not have all this major and minor and just have major. In practise this all gets too complicated and if there is something worrying you put them on antibiotics.

**Original question**	**% agreement**	**Action taken**	**Comments**
Irritability	64%	Change	▪ Not included in WHO or COIN guidelines
**New proposed question**	**Indicate level of agreement**		**Your thoughts/comments**
Irritable/ inconsolable baby	1 2 3 4 5		.

4. Do you think hypothermia < 35.5 °C should be added as a sign of neonatal sepsis in a low-income setting?

Expert responses

It is a major if persistent hypothermia despite warming

It is well recognised as a sign of sepsis. Suggested wording 'Hypothermia on two or more occasions.'

Yes, major. We know sepsis that can present as hypothermia or hyperthermia. It is just as important as a temperature of > 37.5 °C

Yes, minor. Hypothermia not responding to warming measures. Temperature instability is more useful than a specific high or low.

Yes, major but persistent hypothermia because I find many babies especially who are out born to be hypothermic.

Yes, major. Hypothermia despite efforts to warm. Hypothermia is a major problem. We have shown if you do not get your temperature up then your mortality is much higher. I would say this is very significant.

Yes, major. If it is persistent hypothermia despite normal supportive care.

Yes, major. What we have done for our definition of sepsis in the clinical trial that we are running is hypothermia < 35.5 °C after 1 hour of skin to skin. A lot of these babies are environmental, but I think the danger sign for sepsis is when you have a low temperature and you have had the best method of correcting, which is skin to skin contact and it is still low. You need to treat those for possible sepsis.

Yes, major. If it is persistent after addressing the environment of the baby it is a major risk factor

Yes, minor. Temperature instability is common with sepsis and might be a better term to use.

Yes, major. I am very familiar with helping babies to breathe and very familiar with the IMCI. I am not so familiar with COIN and some of this may be COIN specific. But with hypothermia it's more like the transient tachypnoea where if you are hypothermic after birth then that's something related to not drying or warming. However, if you are hypothermic days later that is less likely to be due to the environment. Most of the kids are not septic right away they take 24-40 hours to become septic especially for early onset sepsis. You tend to get that coming through mum's birth canal unless the mum's membrane has been ruptured for a long period of time. Then you may be totally asymptomatic so at first you may have a cold baby because they did not dry the baby that's one thing but there could be another baby that was totally not cold and then sporting a temperature of 35 °C because they became septic. Those are the two scenarios and there are environmental things added to it like if you are living in a cold area. Hypothermia would be more concerning to me later on than right after birth on admission to the neonatal unit. So, having new hypothermia, temperature instability or persistent hypothermia would all be concerning to me.

I personally don't think so but follow WHO.

**Original question**	**% agreement**	**Action taken**	**Comments**
Hypothermia <35.5°C	78%	Change	▪ This is a WHO and COIN danger sign. Experts agreed if hypothermia was persistent despite warming it should be included. However, the app is currently designed for just point of admission so we will only have a one-off temperature reading. Starting antibiotics and considering infection is covered in the hypothermia algorithm if the infant stays hypothermic despite attempts at rewarming.
**New proposed question**	**Indicate level of agreement**		**Your thoughts/comments**
Single measurement of temperature of <35.5°C	1 2 3 4 5		

5. Are there any additional risk factors, signs or symptoms that you think should be included for a low-income setting?

Expert responses

Particularly for preterm babies some of these signs are less specific such as reduced movement of limbs or hypotonia you expect that in preterm babies anyway. There are not

actually any good validated nosocomial scores for preterm babies while they are in hospital so the Rosenberg criteria is probably the best one but it still only has a sensitivity of 77%. It has 5 criteria which are apnoea, pallor, jaundice, hepatomegaly and seizures. What is difficult here is that you need a certain level of clinical skill to pick up the hepatomegaly. I am not saying they should be used here but it may be interesting to look at. Rosenberg RE, Ahmed AS, Saha SK, et al. Nosocomial sepsis risk score for preterm infants in low-resource settings. *J Trop Pediatr*. 2009;56(2):82–89.

Did the mother get any intrapartum antibiotics is probably too difficult to in cooperate as you never know what time etc

Poor capillary refill or perfusion

Hypothermia

Did the mother get any intrapartum antibiotics? Or did the mother get a GA? These factors affect whether the baby will come out hypotonic, tachypnoeic/shallow breathing and these babies are going to get antibiotics.

Eye symptoms. Red eye with pus. It would be a minor.

I don't think so. I would include all the danger signs from the WHO

Does the baby look ill?

**New proposed question**	***Rationale***	**Indicate level of agreement**	**Indicate whether it should be an 'other' risk factor or major (danger) sign**	**Your thoughts/comments**
A. Poor capillary refill or perfusion	*Expert suggestion*	1 2 3 4 5	Other or Major	
B. Unconscious	*WHO danger sign 'not moving when stimulated'*	1 2 3 4 5	Other or Major	
C. Swollen red eyelids with pus	*WHO localising sign of infection*	1 2 3 4 5	Other or Major	
D. Central cyanosis	*WHO danger sign*	1 2 3 4 5	Other or Major	
E. Does the baby look ill?	*Expert suggestion*	1 2 3 4 5	Other or Major	

6. Please comment on the criteria for 'consider meningitis'

Expert responses

I prefer unstable temperature above 38 degrees or less than 36.5 degrees Celsius.

Bulging fontanelle yes, hypotonia yes but would also include hypertonia, floppiness, absent suck.

Remove 37 centimetres because it does not apply to a premature baby and just have obvious enlarging head.

It should be hypertonia or hypotonia. Irritability, lethargy is fine. Hydrocephalus is a very late sign of meningitis so not helpful acutely so should be removed. Convulsions need to be described as abnormal repetitive stereotypical movement.

Yes. That is fine

Hydrocephalus is rubbish. You would not get a head circumference.

I would disagree with the hydrocephalus bit as there could be an anatomical reason for their hydrocephalus.

You don't usually get hydrocephalus very early on in meningitis. This one seems a bit odd to me. I would get rid of it. You can make the diagnosis with bulging fontanelle and everything else.

I have to say if you have hydrocephalus in the acute setting the symptoms or signs of hydrocephalus would be more important than the head circumference.

Add in high pitched cry/convulsive cry and abnormal movements (+/- convulsions). Often baby's seizures are very subtle, it may be abnormal movements of the lips, or slightly abnormal movement of the legs which people don't class as convulsions. Remove lethargy because it is non-specific. A febrile baby who is lethargic does not specifically point to meningitis.

Remove hypotonia it is too complex for nurses to assess for routine care and it should be kept simple.

Remove hydrocephalus it is a post meningitis event and they don't present with it

Overall recommendation: Persistent temperature > 37.5, bulging fontanelle, persistent irritability with a convulsive cry, abnormal movements +/- convulsions

I would not have convulsions alone to diagnose meningitis as it could be birth asphyxia, I would want to see it with a temperature. I would remove hydrocephalus as it could be an anatomical cause.

I would add hypertonia/hypotonia

Agree with all of them

Happy with these.

I am guessing the only reason for diagnosing meningitis is for antibiotic duration.

I would say they need to have a temperature over 38 °C not 37.5 °C. You are going to have a lot of kids here with temperatures of 37.5 °C which seems low. Bulging fontanelle with a temperature of 38 °C seems reasonable. Hypertonia is a tough one to diagnose. A kid who had an in-utero stroke is going to be hypertonic. Do these kids get antibiotics every time they have a fever?

Acute seizures are straight forward. I like the way WHO does it and I do not normally say this because if the baby has convulsions acutely you treat. What if your kid has a seizure

disorder? You would have to give antibiotics constantly which is not going to treat anything because he has a seizure disorder. I would say it has to be acute seizures. Hydrocephalus, new onset? Baby? They pick standard deviations on the head circumference which is probably appropriate for a concern but then you are still treating 2 out of every 100 kids because of they just have a big head. I would say just go with the danger signs of fever, lethargy and convulsions which will give you a secure diagnosis and the rest are not.

I would not include hydrocephalus. Hydrocephalus is a separate issue and a whole different diagnosis

**Original question**	**% agreement**	**Action taken**
Temp >37.5 °C AND any of<br>• Bulging Fontanelle<br>• Hypertonia<br>• Persistent irritability<br>• Lethargy<br>OR<br>• Convulsions/History of convulsions<br>• Hydrocephalus	28% agreement	Change
**New proposed question**	**Indicate level of agreement**	**Your thoughts/comments**
Bulging fontanelle	1 2 3 4 5	
Irritability with a high-pitched cry	1 2 3 4 5	
Drowsy, lethargic or unconscious	1 2 3 4 5	
Abnormal movements/twitching or convulsions with temp >37.5	1 2 3 4 5	
Abnormal tone with temp >37.5	1 2 3 4 5	

Section 2. Hypoxic Ischaemic Encephalopathy

1. Do you agree with the following risk factors for hypoxic ischaemic encephalopathy?

From expert feedback the term 'birth asphyxia,' has been changed to hypoxic ischaemic encephalopathy (HIE). In addition, NeoTree will no longer be using risk factors to aid making a diagnosis of HIE and it will be adjusted to use the already validated Thompson score. However, the risk factors that were agreed upon by experts in the first round will be a prompt on the app for the user to perform the Thompson score.

Birth injury

Expert responses

What do you mean by birth injury it needs to be defined.

As a screening tool it is a valid important thing to think about.

I disagree. Meaning what? This is really ambiguous and needs a description. It could be a facial laceration and that would not give you asphyxia.

I disagree. Need to define further. I would not consider a bruise or a cephalohematoma as an injury. It needs to be specific. However, I would still only consider as there are far more important risk factors which would be more predictive for the infant.

It depends on what the birth injury is. If they come out with a very moulded head, large cephalohematoma, fractured clavicle, erb's palsy then I would assume they have had significant problems extracting. However, something like a laceration I would not consider as a significant birth injury. You need to specify the birth injuries.

I disagree. What are we talking about when we say birth injury here? More detail is needed. Broken bones or significant neurological deficit/nerve injury I would agree with.

You need to be more specific i.e. a fracture or nerve palsy.

Disagree. Too vague. I would not include this one.

This needs to be defined.

Disagree. These need to be defined because it is a very broad term. If there are palsies, cephalohematoma or subgaleal, fractures, extensive bruising then I would agree.

Disagree. For instance, bruising does not normally cause asphyxia.

**Original question**	**% agreement**	**Action taken**	**Comments**
Birth injury as risk factor for HIE	29%	Change	▪ Not included in WHO or COIN guidelines<br>▪ App developer is keen to see if we specify the birth injuries whether this can be included
**How should we describe "significant" birth injury?**			

2. Do you agree with the following signs/symptoms for hypoxic ischaemic encephalopathy?

Absent suck and gestation > 34/40

Expert responses

Disagree. This is not sensitive or specific. It could be because you are septic. I really worry that the approach to this is not scientific and it is not based on existing evidence base. WHO and COIN will have followed a formal process to what is included or excluded. I worry about any of the signs and symptoms that the app developers have suggested that are not evidence based to make a recommendation. Research needs to be done for any suggestions of the app developers and recommendations cannot be made until you have the evidence.

Disagree. I think it should be 35 weeks and this really depends on how old the neonate is. This assessment needs to be at least 1 hour of age as the infant needs some transition time.

Disagree. I would have the gestation at 32/33 weeks and then I would agree. For the rest I agree but my comment around these questions is that if you are measuring these signs and symptoms too early then you can over diagnose. Often babies that have a lactic acidosis very early on but may not have a significant amount of hypoxia may be obtunded but then recover very quickly as oppose to a baby that has had a significant hypoxic hit and you need to wait at least an hour.

Disagree. I think the only reliable clinical signs are seizures, coma, flaccid and very poor tone. I would really use the Sarnat score or the Thompson criteria here. However, it is difficult to assess for HIE using the Sarnat score for preterm babies as it has mainly been designed for term babies. The Thompson score which I think was designed in East Africa has a much more pragmatic approach and I would use that to guide here.

Disagree. Just use the sarnat stages.

**Original question**	**% agreement**	**Action taken**	**Comments**
Absent suck and gestation >34/40 weeks	57%	Change but include as part of the Thompson score	
**New proposed question**	**Indicate level of agreement**		**Your thoughts/comments**
Absent suck and gestation >32/40 weeks	1 2 3 4 5		

Section 3. Respiratory distress of the newborn (RDN)

1. Do you agree with the diagnostic criteria for Respiratory Distress of the Newborn (RDN):

From expert feedback the NeoTree application will no longer generate a respiratory diagnosis (respiratory distress syndrome, meconium aspiration, transient tachypnoea of the newborn) and it will just guide you to treating the symptoms. However, the above possible diagnoses will still be included as teaching points. NeoTree allows for multiple diagnoses i.e the preterm baby can have a diagnosis of RDS and pneumonia.

A. Respiratory distress syndrome

Expert responses

Lower gestation to 34-35 weeks. Older than that they are not at risk.

Agree. It's reasonable. You have to both

Or diabetic mother

Yes agree. Include weight for suggested gestation.

I would say a lower gestation. The risk for a 36 weeker is relatively low. I would change gestation to weight and say < 2 kg.

1 disagree use weight < 2000g

I would say less than 34 weeks

Disagree in making a diagnosis. Just treat the symptoms

**Original question**	**% agreement**	**Action taken**	**Comments**
Respiratory Distress Syndrome	50%	Change	
**New proposed question**	**Indicate level of agreement**		**Your thoughts/comments**
Gestation <34/40 or <2000g	1 2 3 4 5		

B. Congenital pneumonia

Expert responses

I would change temperature to more than 38 °C or less than 36.5.

I would say hypothermia or hyperthermia and if you mention temperature you have to say both.

Not so sure I agree with foul smelling liquor

I would remove unilateral signs as pneumonia can be bilateral so just say coarse crackles.

I think the temperature should be more than 38 °C.

Disagree. Again, in my opinion, I would treat the symptoms and do not worry about diagnosing. For example, for congenital pneumonia, at CDC we had a whole panel of all the experts trying to agree on how to diagnose pneumonia. The decision of the panel was that it is impossible in a developed country to diagnose neonatal pneumonia. So, if you can't do it here how do you expect them to do it there. The temperature should also be more than 38 °C.

Again, I don't think you need risk factors. You are going to diagnose it even if there are no risk factors but it's fine.

**Original question**	**% agreement**	**Action taken**	**Comments**
Congenital Pneumonia	50%	Change	
**New proposed question**	**Indicate level of agreement**		**Your thoughts/comments**
Temp >37.5ºC or <36.5ºC	1 2 3 4 5		
Coarse crackles (instead of unilateral signs)	1 2 3 4 5		
