# Supplementary material for: Refining clinical algorithms for a neonatal digital platform for low-income countries: a modified Delphi technique
Source: BMJ Open. 2021 May 18;11(5):e042124. doi: 10.1136/bmjopen-2020-042124 (PMC8130744; doi:10.1136/bmjopen-2020-042124)

ORIGINAL
Sepsis algorithm

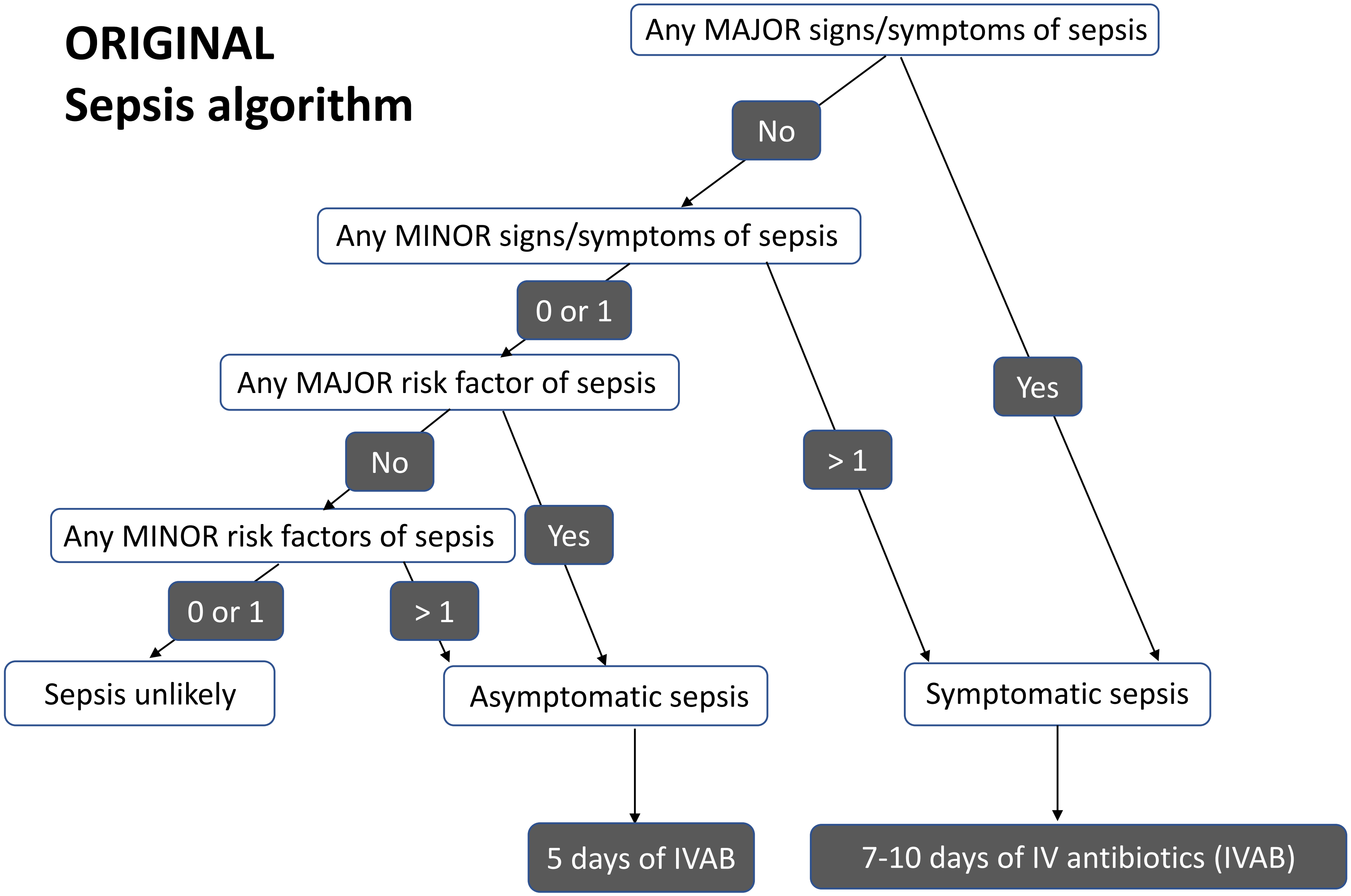

REVISED Sepsis algorithm

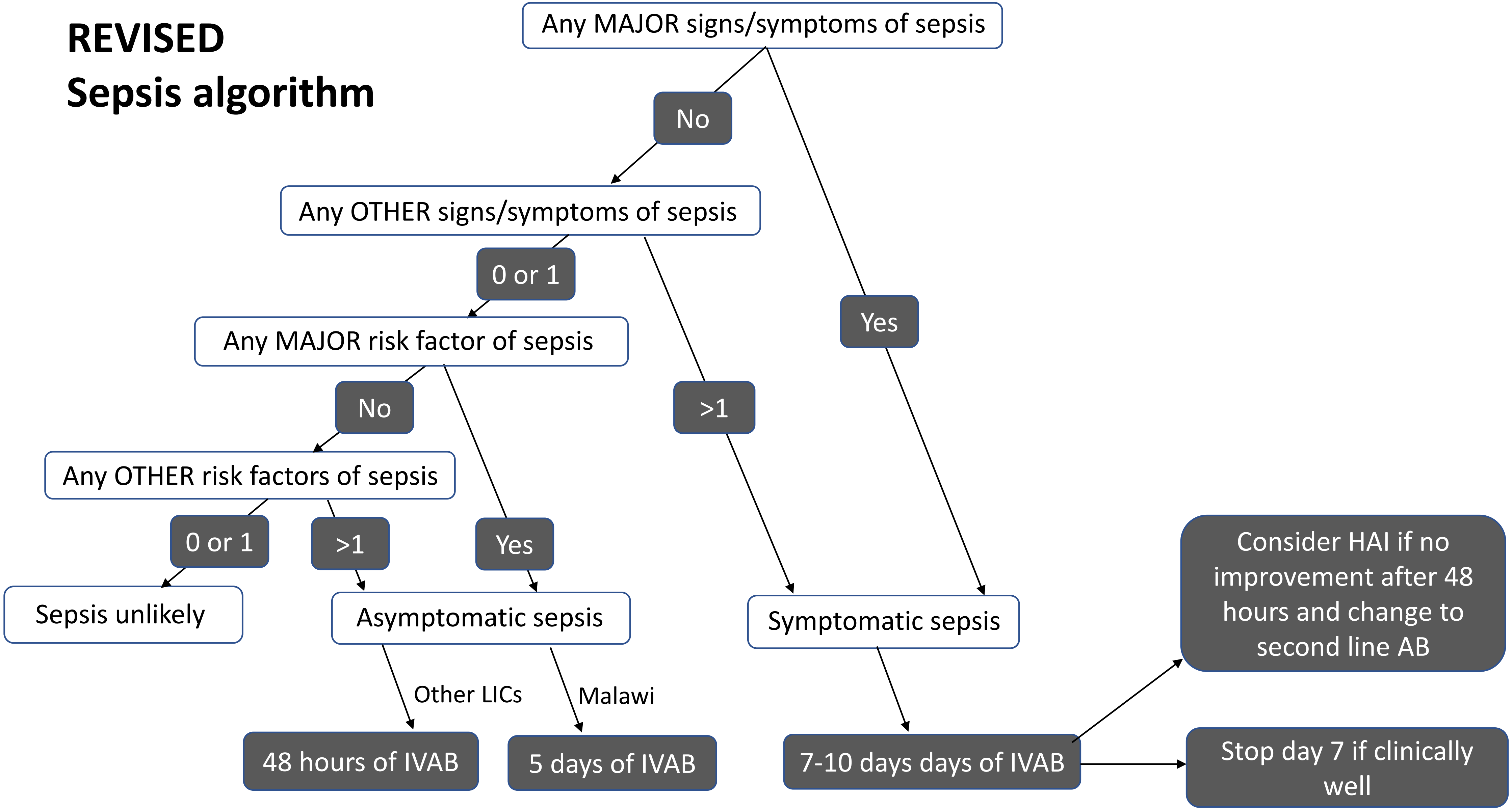

Revised neonatal sepsis risk factors, signs and symptoms

Risk factors

Major
- Maternal fever >38°C in labour
- Prolonged rupture of membranes (PROM) >18 hours
- Foul smelling amniotic fluid

Other risk factors
- <32/40 weeks gestation or <1500g (spontaneous labour only)

Signs/symptoms

Major
- Neonatal temperature >37.5 °C
- Boil/abscess
- Grunting/severe respiratory distress/ mod-severe WOB
- Lethargy
- Umbilical redness extending to the periumbilical skin or umbilicus draining pus
- Deep jaundice: palms and soles of the baby deep yellow
- Tachypnoea > 60 bpm
- Convulsions/twitching or abnormal movements
- Many or severe skin pustules
- Bilious vomiting with severe abdominal distension
- Bulging fontanelle
- New onset of poor feeding
- Not moving when stimulated
- Swollen red eyelids with pus
- Central cyanosis

Other signs/symptoms
- Pallor

BMJ Publishing Group Limited (BMJ) disclaims all liability and responsibility arising from any reliance placed on this supplemental material which has been supplied by the author(s)

Revised neonatal sepsis management

Neonatal sepsis management

Investigations

- *If possible* perform sepsis screen: FBC, CRP, blood culture
- LP only for those who have clinical signs of meningitis (but stable) or late-onset neonatal sepsis

Antibiotic choices

- 1st line: give local recommendations
- IM/IV gentamicin and benzylpenicillin **or** ampicillin
- 2nd line: If no improvement after 48 hours change to a third-generation cephalosporin
- IV cloxacillin and gentamicin if greater risk of staphylococcal (skin infection).

Antibiotic duration (without investigations)

Asymptomatic sepsis: Treat for 48 hours and stop if well (Treat for 5 days in Malawi)
Symptomatic sepsis: Stop at day 7 if clinically well otherwise continue for 10 days

Antibiotic doses

Gentamicin (IM/IV)
<7 days: 3 mg/kg (LBW) and 5 mg/kg (normal BW) per dose once a day
>7 days 7.5 mg/kg once a day

Ampicillin (IM/IV)
<7 days: 50 mg/kg every 12 h
>7 days every 8 h

Benzylpenicillin (penicillin G) (IM)
<7 days: 50 000 U/kg every 12 h
>7 days every 6 h

Supportive care

- Thermoregulation: aim for 36.5-37.5 °C
- Provide respiratory support oxygen or CPAP as needed according to CPAP algorithm
- Check blood glucose and provide feeding support as needed
- Consider 10ml/kg fluid bolus if shocked as per SHOCK algorithm
- Consider 2.5mls/kg 10% dextrose if BS<2.5 mmol or 45 mg/dl – as per HYPOGLYCAEMIA algorithm

ORIGINAL
Birth Asphyxia algorithm

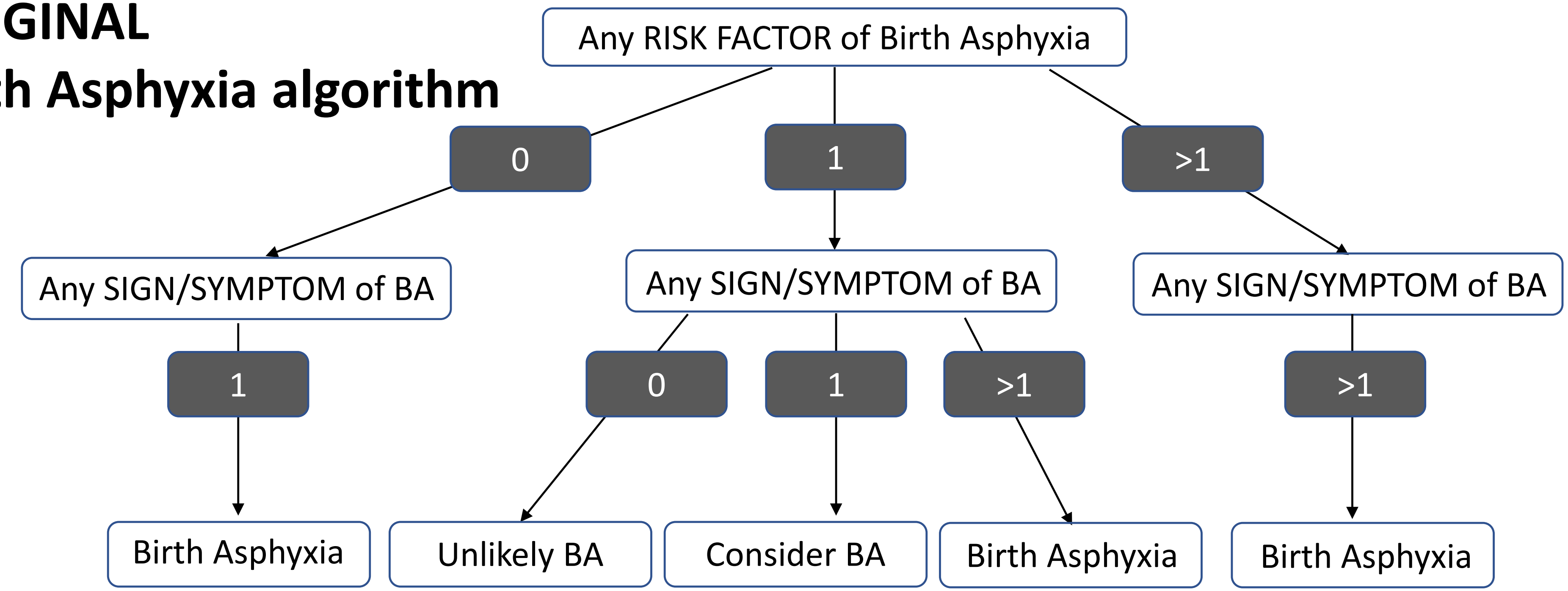

Signs/symptoms

- Coma
- Convulsions
- Lethargy
- Irritable
- Hypotonia AND >34/40 gestation
- Absent suck AND >34/40 gestation

Risk factors

- Foetal distress
- Prolonged second stage
- Delivery = Vacuum/Emergency Caesarian section/Breech
- 5 minute Apgar <7
- Resuscitation: Bag valve mask >5 mins/CPR/resuscitation >10 mins
- Birth Injury

REVISED Hypoxic Ischaemic Encephalopathy algorithm

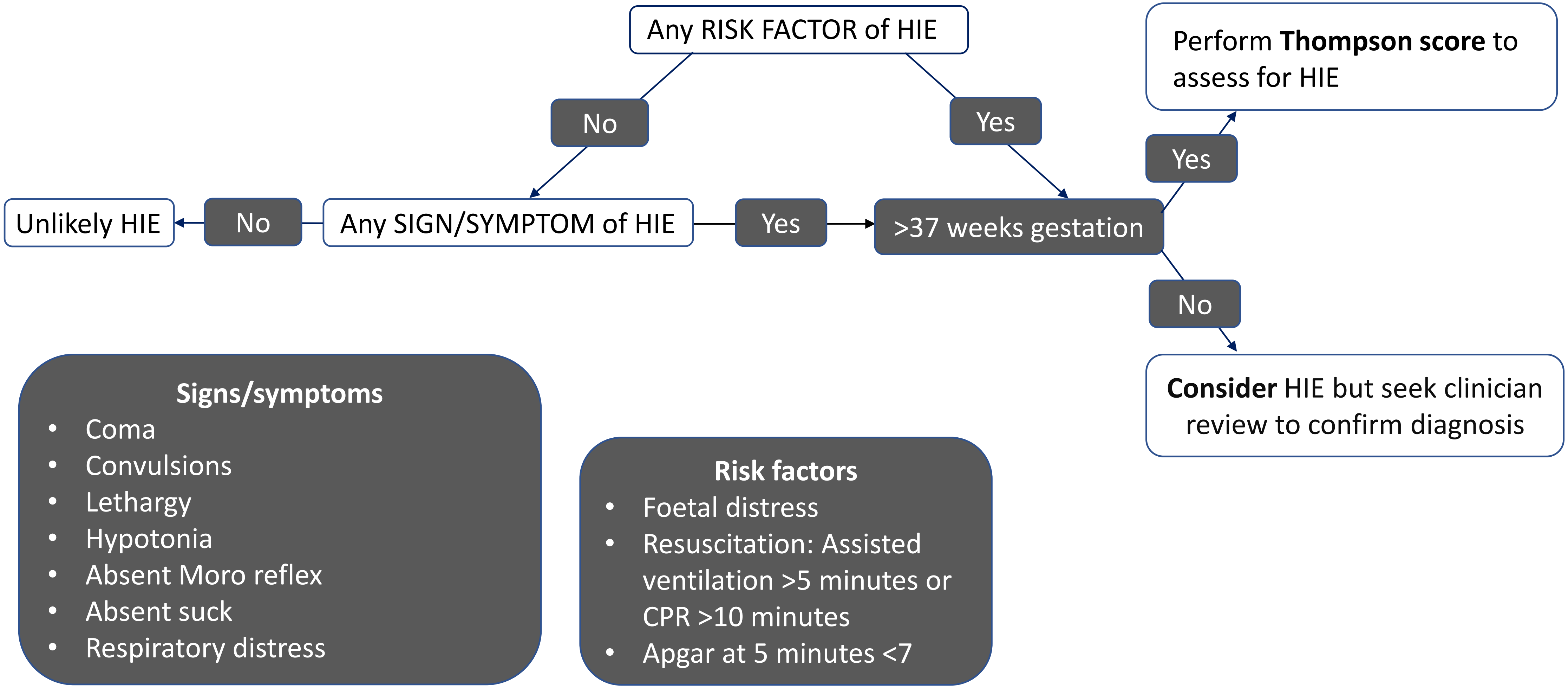

Revised Hypoxic Ischaemic Encephalopathy management

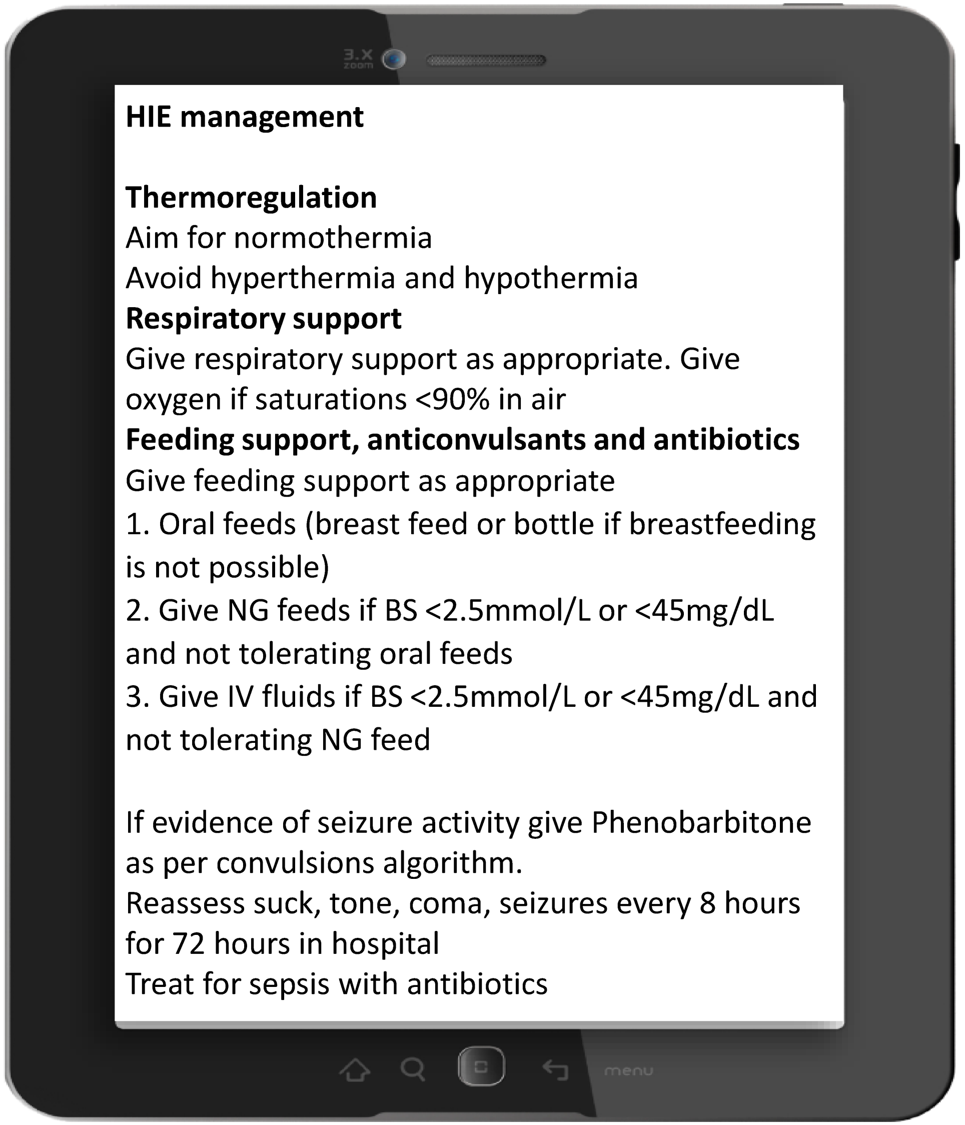

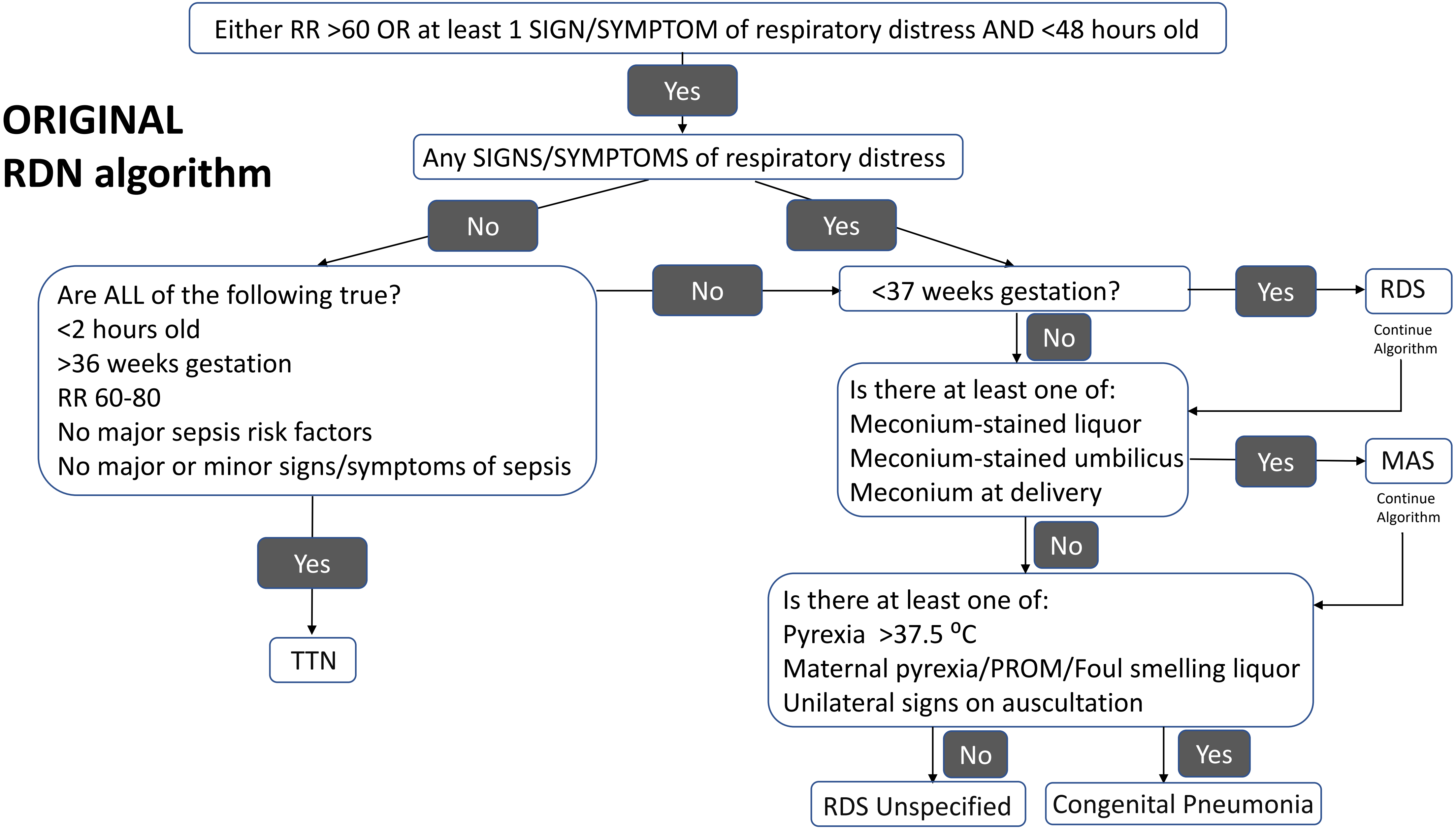
ORIGINAL
RDN algorithm
Either RR >60 OR at least 1 SIGN/SYMPTOM of respiratory distress AND <48 hours old
Yes
Any SIGNS/SYMPTOMS of respiratory distress
No
Yes
Are ALL of the following true?
<2 hours old
>36 weeks gestation
RR 60-80
No major sepsis risk factors
No major or minor signs/symptoms of sepsis
No
<37 weeks gestation?
Yes
RDS
Continue Algorithm
No
Yes
TTN
Is there at least one of:
Meconium-stained liquor
Meconium-stained umbilicus
Meconium at delivery
Yes
MAS
Continue Algorithm
No
Is there at least one of:
Pyrexia >37.5 ºC
Maternal pyrexia/PROM/Foul smelling liquor
Unilateral signs on auscultation
No
Yes
RDS Unspecified
Congenital Pneumonia

BMJ Publishing Group Limited (BMJ) disclaims all liability and responsibility arising from any reliance placed on this supplemental material which has been supplied by the author(s)

REVISED
RDN management

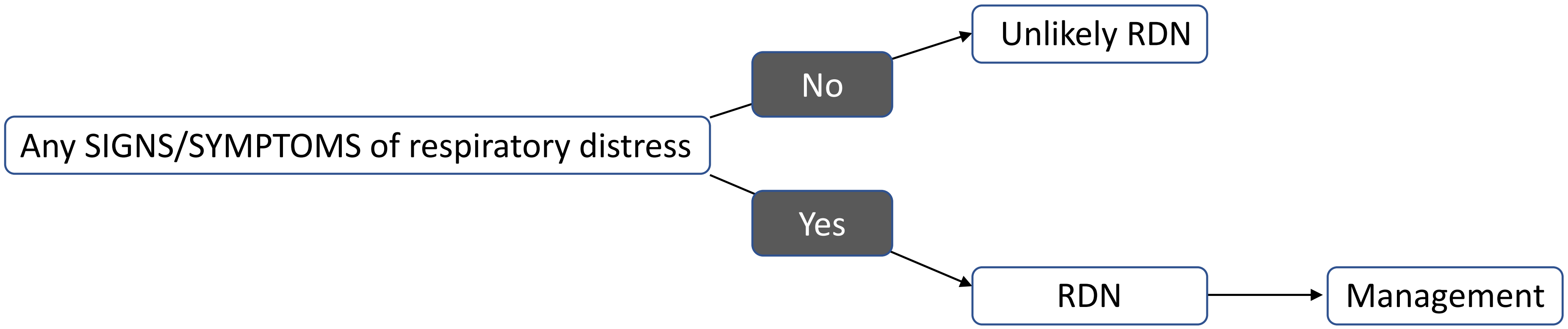

Signs/symptoms of respiratory distress

- Oxygen saturations in air <90%
- Tachypnoea >60 breaths per minute
- Fever >37.5^{o}C or <36°C and crepitations on auscultation
- Grunting
- Recessions
- Any increased work of breathing
- Cyanosis
- Apnoea

Revised Respiratory Distress of the Newborn management

Respiratory distress of the newborn management

Investigations
Perform a chest x-ray if it will change management or if the infant is not improving as expected or deteriorating.

Airway and respiratory support
Position airway
Give oxygen if oxygen saturations < 90% in air
If > 1kg consider CPAP according to CPAP algorithm

Feeding support
If breathing 60-80 bpm use cup/NGT
If needing CPAP use OGT
If breathing > 80bpm consider IV fluids

IV Access for Antibiotics
Give antibiotics including for suspected TTN (unless have chest x-ray and can safely exclude.)

Teaching points

Meconium aspiration
- Consider if meconium stained amniotic fluid
- Just because there was meconium at delivery doesn't mean it was aspirated.
- Most aspiration occurs in utero before the baby is born, so more likely if there was foetal distress in labour or has signs of HIE
- Floppy babies with severe HIE and meconium aspiration are unlikely to benefit from CPAP.

Transient tachypnoea of the newborn (TTN)
- These babies are not unwell
- RR is usually between 60-80 bpm and they don't usually need oxygen
- This should resolve in the first few hours of life.
- TTN is more likely following elective C-section or a rapid vaginal delivery.
- Give antibiotics if unable to exclude other causes i.e no chest xray

Respiratory distress syndrome
- Consider if < 37 weeks or diabetic mother
- Benefit from CPAP see Algorithm

Congenital Pneumonia
- Consider if temperature > 37.5 ^{o} c or < 36 ^{o} c and crepitations on auscultation
- Risk factors for sepsis i.e maternal fever/PROM/foul smelling amniotic fluid

Hypothermia Algorithm and management

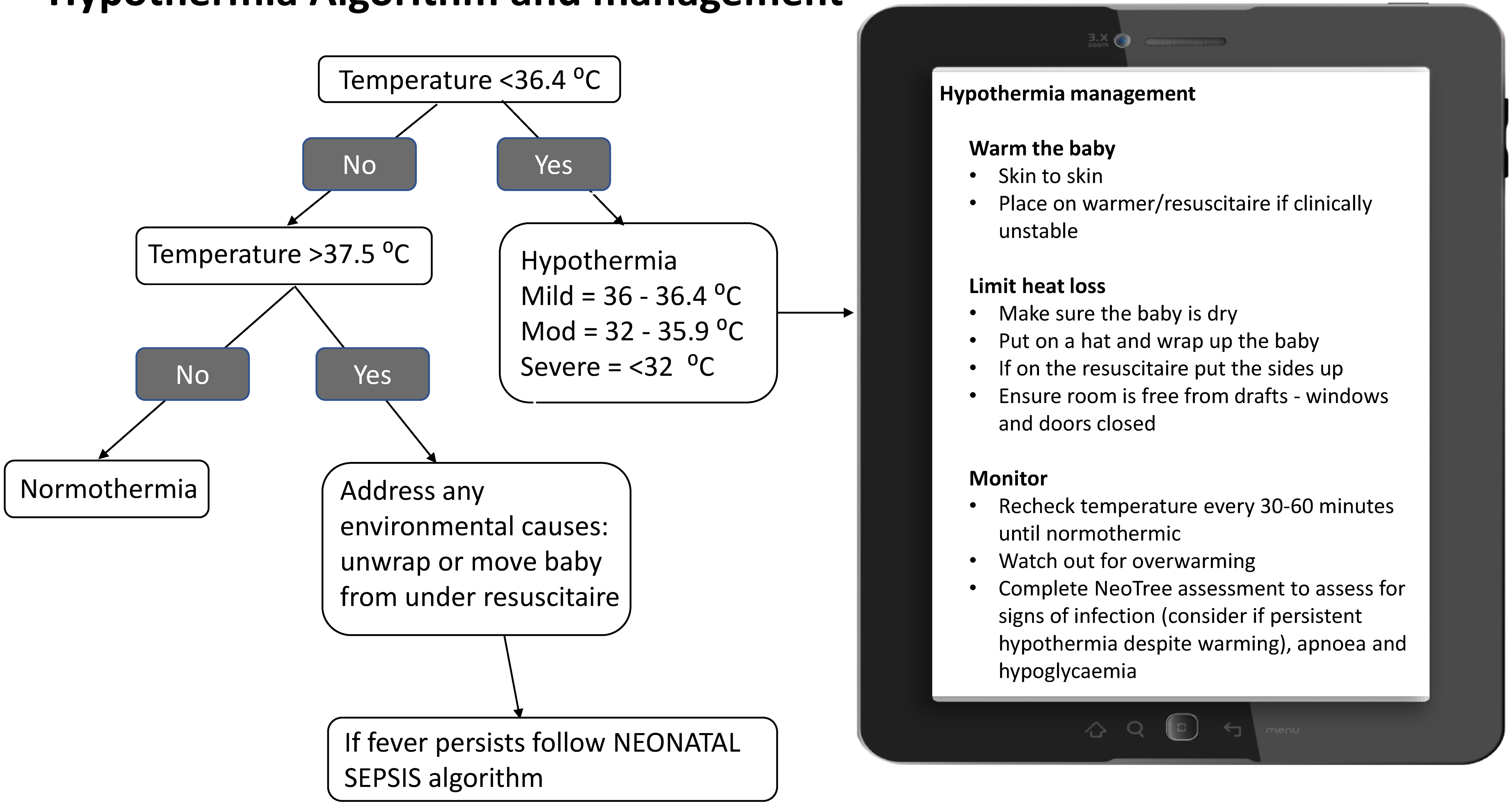

Supplement: Supplementary data [file bmjopen-2020-042124supp002.pdf]
