# Supplementary material for: Refining clinical algorithms for a neonatal digital platform for low-income countries: a modified Delphi technique
Source: BMJ Open. 2021 May 18;11(5):e042124. doi: 10.1136/bmjopen-2020-042124 (PMC8130744; doi:10.1136/bmjopen-2020-042124)
Supplement: Author's manuscript [file bmjopen-2020-042124.draft_revisions.pdf]

BMJ Open

BMJ Open is committed to open peer review. As part of this commitment we make the peer review history of every article we publish publicly available.

When an article is published we post the peer reviewers' comments and the authors' responses online. We also post the versions of the paper that were used during peer review. These are the versions that the peer review comments apply to.

The versions of the paper that follow are the versions that were submitted during the peer review process. They are not the versions of record or the final published versions. They should not be cited or distributed as the published version of this manuscript.

BMJ Open is an open access journal and the full, final, typeset and author-corrected version of record of the manuscript is available on our site with no access controls, subscription charges or pay-per-view fees (http://bmjopen.bmj.com).

If you have any questions on BMJ Open's open peer review process please email info.bmjopen@bmj.com

BMJ Open

Refining clinical algorithms for a neonatal digital platform for low-income countries: A modified Delphi technique

Journal:	*BMJ Open*
Manuscript ID	bmjopen-2020-042124
Article Type:	Original research
Date Submitted by the Author:	26-Jun-2020

Complete List of Authors:	Evans, Mari; Great Ormond Street Hospital for Children, UCL Institute of Child Health<br>Corden, Mark; Children's Hospital of Los Angeles, Division of Hospital Medicine, Department of Pediatrics; University of Southern California Keck School of Medicine, Pediatrics<br>Crehan, Caroline; Great Ormond Street Hospital for Children, UCL Institute of Child Health<br>Fitzgerald, Felicity; Great Ormond Street Hospital for Children, UCL Institute of Child Health<br>Heys, Michelle; Great Ormond Street Hospital for Children, UCL Institute of Child Health

Keywords:	PUBLIC HEALTH, Information technology < BIOTECHNOLOGY & BIOINFORMATICS, Neonatal intensive & critical care < INTENSIVE & CRITICAL CARE, NEONATOLOGY

Note: The following files were submitted by the author for peer review, but cannot be converted to PDF. You must view these files (e.g. movies) online.

Online supp 1 - questionnaire.docx

SCHOLARONE™
Manuscripts

BMJ

I, the Submitting Author has the right to grant and does grant on behalf of all authors of the Work (as defined in the below author licence), an exclusive licence and/or a non-exclusive licence for contributions from authors who are: i) UK Crown employees; ii) where BMJ has agreed a CC-BY licence shall apply, and/or iii) in accordance with the terms applicable for US Federal Government officers or employees acting as part of their official duties; on a worldwide, perpetual, irrevocable, royalty-free basis to BMJ Publishing Group Ltd ("BMJ") its licensees and where the relevant Journal is co-owned by BMJ to the co-owners of the Journal, to publish the Work in this journal and any other BMJ products and to exploit all rights, as set out in our licence.

The Submitting Author accepts and understands that any supply made under these terms is made by BMJ to the Submitting Author unless you are acting as an employee on behalf of your employer or a postgraduate student of an affiliated institution which is paying any applicable article publishing charge ("APC") for Open Access articles. Where the Submitting Author wishes to make the Work available on an Open Access basis (and intends to pay the relevant APC), the terms of reuse of such Open Access shall be governed by a Creative Commons licence – details of these licences and which Creative Commons licence will apply to this Work are set out in our licence referred to above.

Other than as permitted in any relevant BMJ Author's Self Archiving Policies, I confirm this Work has not been accepted for publication elsewhere, is not being considered for publication elsewhere and does not duplicate material already published. I confirm all authors consent to publication of this Work and authorise the granting of this licence.

For peer review only

Refining clinical algorithms for a neonatal digital platform for low-income countries: a modified Delphi technique

Mari Evans,[1*] Mark H. Corden,[2,3*] Caroline Crehan,[1] Felicity Fitzgerald, [1] Michelle Heys[1,4]

[1]Great Ormond Street Institute of Child Health, University College London, London, UK
[2]Division of Hospital Medicine, Department of Pediatrics, Children's Hospital Los Angeles, Los Angeles, CA, USA
[3]Department of Pediatrics, Keck School of Medicine, University of Southern California, Los Angeles, CA, USA
[4]Specialist Children's and Young People's Services, East London NHS Foundation Trust, London, UK

[*]denotes co-first authors

Corresponding Author:
Mari Evans
c/o Dr Michelle Heys
UCL Great Ormond Street Institute of Child Health
30 Guilford Street
London, WC1N 1EH
United Kingdom
mari.evans@doctors.org.uk

Word count: 3825

For peer review only

ABSTRACT

Objectives

To determine whether a panel of neonatal experts could address evidence gaps in neonatal guidelines by reaching a consensus on four clinical decision algorithms for a neonatal digital platform (NeoTree).

Design

Two-round, modified Delphi technique.

Setting and participants

Participants were neonatal experts from high-income and low-income countries (LICs).

Methods

This was a consensus-generating study. In round one, experts rated items for four clinical algorithms (neonatal sepsis, hypoxic ischaemic encephalopathy, respiratory distress of the newborn, hypothermia) and justified their responses. Items meeting consensus (≥80% agreement) were included. Items not meeting consensus were either excluded, included following revisions or included if they contained core elements of evidence-based guidelines. In round two, experts rated items from round one that did not reach consensus.

Results

Fourteen experts participated in round one, ten in round two. Nine were from high-income countries, five from LICs. Experts included physicians and nurse practitioners with an average neonatal experience of 20 years, 12 in LICs. After two rounds, a consensus was reached on 43 of 84 items (52%). Experts consistently stated that items must be in line with local and WHO guidelines (irrespective of the level of supporting evidence or expert opinion). As a result, the final algorithms included 53 items (62%).

For peer review only

Conclusion

Four algorithms in a neonatal digital platform were reviewed and refined by consensus expert opinion. Revisions to the NeoTree application were made in response to these findings and will be clinically validated in an imminent study.

KEYWORDS

Information technology, neonatal intensive & critical care, neonatology, public health

For peer review only

STRENGTHS AND LIMITATIONS OF THIS STUDY

- In this study, a large number of algorithm items were reviewed and evaluated, and half met consensus for inclusion in the management pathways.
- The review was conducted with experts from a broad range of countries and neonatal experience who simultaneously refined the algorithms and highlighted gaps in current evidence, emphasising the need for future research to support international neonatal guidelines.
- Our study method meant that experts were not able to meet in person, which might have promoted dialogue that would have allowed greater clarity in their collective opinion.
- The representation of neonatal experts from LICs was not as robust as from high-income countries, which may have led to an uneven evaluation of the algorithms.

For peer review only

INTRODUCTION

Globally, 2.5 million newborns die each year in the first 28 days of life.[1] Most of these deaths (98.5%) occur in low-income countries (LICs), and 40% occur on the first day of life.[2] The neonatal mortality rate has been halved since 1990,[3] but modelling of global newborn mortality data suggests that a further two thirds of current deaths could be prevented if evidence-based solutions were implemented.[2] One of the World Health Organisation (WHO) Sustainable Development Goals (SDG3) is to end preventable deaths of newborns in all countries and to reduce the neonatal mortality rate from the current rate of 18 per 1000 live births to less than 12 per 1000 by 2030.[4] Targeting newborn care in LICs is thus an urgent priority, especially the three most common causes of mortality – infections (36%), prematurity (28%), and intrapartum complications (23%).[2]

Mobile health (m-health) technology and digital platforms are potential approaches to improve the quality of newborn care. They are tools with broad applicability (three quarters of the world now own mobile phones) that are scientifically rigorous and tailored to local patient and healthcare worker (HCW) preferences and resources.[5] A study in Tanzania improved adherence to the Integrated Management of Childhood Illness protocol and assessments of acutely unwell children with an electronic, compared to paper-based, format.[6] M-health and digital platforms can also provide clinical decision algorithms which translate guidelines into context-specific and user-friendly diagnostic aids and management plans. Community HCWs in rural Bangladesh were trained to use a mobile application for neonatal assessment and improved their clinical evaluations, the accuracy of their diagnoses, and their adherence to neonatal management guidelines.[7] Other attempts have been made to introduce health technologies, but most are not available when and where they are needed.[8]

For peer review only

An international team of researchers, clinicians and software developers in the UK, USA, Malawi, Bangladesh and Zimbabwe co-designed and co-developed with Malawian and Zimbabwean HCWs a neonatal digital platform (NeoTree) for facility-based newborn care. It combines immediate digital data capture (which is shared with HCWs via local dashboards), evidence-based algorithmic clinical decision and management support, newborn education and data linkage to national data systems on one platform.[9] The algorithms in the Malawian version of the NeoTree support decisions according to established international[10] and Malawian neonatal guidelines.[11] Local guidelines were combined with international guidelines to accommodate local conditions and to encourage buy-in from local stakeholders. NeoTree is in the phase of application co-development where appropriate revisions to the algorithms are needed to address gaps in evidence for the guidelines.[12] In the absence of extensive trial or epidemiological data, alternative techniques to consolidate best available lower level evidence can be used, such as expert opinion. This study aims to use the modified Delphi technique to determine whether a panel of experts in newborn care can reach a consensus opinion about key clinical decision algorithms used in a digital platform to assist HCWs caring for facility-based unwell newborns in LICs.

METHODS

Study design

This study used a two-step modified Delphi technique.[13] The Delphi technique was chosen because it is an effective method of gathering expert knowledge from geographically diverse leaders in the field to address complex clinical problems that lack evidence.

Recruitment

For peer review only

Twenty-two neonatal experts were invited to participate in the study. This number represented an adequate sample size[14, 15] and permitted a manageable amount of data collection. Participants were recruited if they were a physician or neonatal nurse practitioner with more than ten years neonatal experience (at least three in LICs), neonatal postgraduate training, fluency in English, internet access and willingness to participate. Neonatal experts known to the researchers for their clinical expertise, research and contributions to guideline development in LICs were identified in equal numbers from both LICs and HICs. No financial incentive was offered, but reimbursement for costs of Skype calls was provided for some experts in LICs.

Algorithms and item generation

The four clinical decision algorithms selected for review were neonatal sepsis, hypoxic ischaemic encephalopathy (HIE), respiratory distress of the newborn and hypothermia. These conditions represent the leading preventable causes of neonatal mortality and are the most difficult to diagnose and manage appropriately in LICs with some of the weakest WHO GRADE recommendations and quality of evidence.[16] For example, the European definition of neonatal sepsis is two or more clinical symptoms and two or more laboratory signs in the presence of, or as a result of, suspected or proven infection.[17] This definition is not possible in LICs where laboratory investigations are not routinely available.

Items were identified by comparing the algorithms side-by-side with the international (WHO) and local neonatal guidelines (Care of the Infant and Newborn in Malawi - COIN) from which they had been derived. This comparison generated a comprehensive list of items where discrepancies in diagnostic parameters and treatment recommendations required expert opinion. Once finalised, the clinical algorithms and list of items (henceforth referred to as questionnaire)

For peer review only

were piloted with two paediatricians with neonatal experience in LICs. Ambiguous items were amended accordingly.

Delphi technique

The questionnaire was circulated by email to the experts with specific instructions at least two weeks before they were interviewed. Each algorithm was verbally and diagrammatically explained with their references specified (i.e., WHO, COIN or NeoTree research team) to aid in decision-making during the interview (online supplementary file 1). Round one interviews were conducted in June and July 2018. Experts were sent up to two reminder emails to schedule their phone or Skype interview. Interviews were conducted privately from a home office. Standardised questions were used to review each item from the questionnaire. Experts were asked to rate their level of agreement for each item using a five-point Likert scale.[18] Each rating was followed by open-ended questions to obtain the experts' rationale for their response and any amendment or additional items they would propose. All interview data were transcribed using both audio-recordings and notes made during the interview by the facilitator. All responses were anonymised (with participant numbers) and reviewed together with the quantitative results. Based on sample size, the level of agreement for consensus opinion from the panel was set at 80%.[14] Items that met consensus (≥80% agreement) were included or were modified with minor changes to wording based on expert advice. Items that did not meet consensus (<80%) were removed or modified according to the feedback from the expert panel and submitted for the second round. Items that did not meet consensus were still included if they were part of WHO and COIN guidelines so that frontline HCWs continued to follow the current standard of care. A second questionnaire was designed with modified items and expert additions from the first round.

For peer review only - http://bmjopen.bmj.com/site/about/guidelines.xhtml

In round two (June and July 2019), this second questionnaire was distributed electronically to the 14 experts from round one (online supplementary file 1). A results summary from round one was sent to the experts, and the full set of anonymised results were made available at their request. Two email reminders were sent to non-responders. Experts again rated items on a Likert scale and explained their ratings. Responses were analysed as described in round one, and items meeting ≥80% consensus were kept for the final NeoTree algorithms.

Patient and public involvement

While key stakeholders were involved in co-developing the NeoTree digital platform, there was no patient or public involvement in this Delphi study.

Consent procedures

The goals and processes of the project were explained to the experts in their email invitation, and consent was obtained by email agreement. Experts were verbally informed at the beginning of the first round that their responses would be kept anonymous.

RESULTS

Twenty-two neonatal experts were invited to participate. Sixteen responded; one declined due to lack of financial incentive, and one declined due to conflict of interest. Demographics of the expert panel are listed in Table 1.

Table 1. Characteristics of the Delphi panel from round one

	**Characteristics**	**N = 14 (%)**
Location	Experts from HICs	9 (64)
	Experts from LICs	5 (36)
Level of expertise	Neonatologist	6 (43)
	Paediatrician	6 (43)

For peer review only

	ANNP	2 (14)
Years of neonatal experience post medical school (mean ± SD)	Overall	20 (± 12)
	In LICs	12 (± 7)
Work experience in low-income countries	Africa	14 (100)
	Asia	7 (50)
	Central America	4 (28)
Country of medical degree	United Kingdom	7
	United States	2
	South Africa	2
	Zimbabwe	1
	Rwanda	1
	Sudan	1

ANNP, advanced neonatal nurse practitioner; HICs, high-income countries; LICs, low-income countries

Round One

Fourteen experts (63% response rate) completed round one. Interviews averaged 73 minutes (40-110 minutes). Thirty-four items (45%) reached consensus (Figure 1). These items were either: (1) included, unmodified (32%); (2) included, modified (11%); or (3) changed for clarification in the second round (2%). Items that did not reach consensus (55%) were either: (1) excluded from the revised algorithm (30%); (2) included because they were part of WHO/COIN guidelines or the Thompson score (11%);[19] or (3) changed and submitted for a second round (14%) (Table 2).

The expert panel consistently stated that algorithm items must comply with WHO danger signs and COIN guidelines for neonatal sepsis, irrespective of whether the panel agreed with them. For example, experts thought that 'bulging fontanelle' was "*subjective; there are non-infectious causes … many babies' fontanelles bulge when they just cry.*" Another item that did not meet

For peer review only

consensus was 'poor feeding,' which experts found vague for multiple reasons, including*: "it is too subjective; it depends on how long for … many newborns do not feed well on the first day of life.*" However, experts agreed that poor feeding was a sign of possible sepsis if it was *"a new onset of poor feeding when the infant had previously been feeding well."* This item was changed to 'new onset of poor feeding' for the final algorithm.

Two items that were included because they are part of the COIN guidelines highlighted inconsistencies with WHO guidelines. For example, COIN uses a temperature of more than 37.5°C as a fever for a newborn, whilst WHO and most experts use more than 38°C. Therefore, 37.5°C was included for the Malawian digital platform, but 38°C will be used for other countries. Other items where a difference between the recommendations and the guidelines occurred were antibiotic choice and duration for neonatal sepsis.

Modifications usually involved adopting the language utilized by WHO or COIN, but there were items that experts felt needed clarifying. For example, experts felt that *"twitching or abnormal movements"* needed to be added to the WHO term 'convulsions' because seizures in a neonate can be very subtle. Certain items that that could not be revised easily were submitted for the second round according to feedback from the expert panel. For example, experts disagreed that 'very/extremely premature (<32 weeks gestation)' was a major risk factor for sepsis if *"the baby was delivered as a clean cold caesarean section for maternal reasons and the mother was not in labour."* Eighty percent of experts highlighted that the algorithm should include weight to guide gestation because "*gestation is often unknown*" and "*you are relying on [the] Ballard score which has plus or minus two weeks accuracy."* Similar opinions regarding method of delivery and the importance of birth weight were expressed for 'slightly premature (32-36 weeks

gestation).' Both gestational age brackets were submitted into the second round as risk factors for sepsis after modifying the items to include WHO weight parameters to guide gestation. Other items that did not gain consensus and were submitted for the second round included items that experts felt needed further clarification. 'Born before arrival' as a minor risk factor for sepsis was clarified to the experts that this meant the baby was born en-route to the hospital (either in a vehicle or on the roadside, both being considered dirty environments in Malawi). A 'neonate admitted with or history of a fever' as a minor risk factor for sepsis was changed to 'mother reports a non-measured fever' in the second round. Lastly, because experts considered the term 'birth injury' unclear, we asked them in round two to define what they considered a 'significant birth injury.'

Table 2. Round one heat chart to show which items met consensus and their outcomes.

Subject	Items	Agree	Outcome
**Sepsis Diagnosis** Major RF	Maternal fever >38°C in labour	91%	Include
	PROM > 18 hrs	74%	Include (WHO RF)
	Offensive smelling liquor	74%	Include (WHO RF); MCTW
	Very / extremely premature (<32/40 weeks)	74%	Second round
	Prolonged second stage (>3 hrs)	53%	Exclude
Minor RF	Prematurity (32-37 weeks gestation)	81%	Second round
	Born before arrival	70%	Second round
Major signs/ symptoms	Boil/abscess	93%	Include
	Grunting or severe respiratory distress or moderate-severe work of breathing	97%	Include
	Lethargy	93%	Include
	Red skin all around umbilicus	81%	Include, MCTW
	Jaundice <24 hrs old	83%	Include, MCTW
	Tachypnoea >60 bpm (>2 hrs old)	83%	Include, MCTW
	Convulsions	89%	Include, MCTW
	Pustules all over body	80%	Include, MCTW
	Bulging fontanelle	77%	Include (WHO danger sign)
	Temperature >37.5°C	73%	Include for Malawi version
	Admitted with or history of fever	68%	Second round
	History of apnoea	67%	Exclude
	Bilious vomiting	61%	Second round
Minor signs/ symptoms	Tachypnoea 60-80 bpm & <2 hrs old	85%	Include
	Pallor	81%	Include
	Weak or absent suck (AND >34/40 weeks)	73%	Exclude

	Poor feeding	73%	Include (WHO danger sign), MCTW
	Irritability	70%	Second round
	Distended abdomen	67%	Second round
	Heart rate >160 that cannot be explained by fever/crying	64%	Exclude
	Mild work of breathing	55%	Exclude
Additional RF?	Cut-off at 72 hrs for early vs late neonatal sepsis?	100%	Include
	Definition of maternal fever >38°C?	93%	Include
	Should PROM be >18 hrs or >24 hrs in LICs?	93%	Include >18 hrs
	Hypothermia < 35.5 °C	83%	Second round
	Fever in a newborn should be classified as >37.5°C in this setting?	83%	Include
	Please comment on our weighting system of Major = 100% / Minor = 50%	77%	Exclude
	Cut-off at >34/40 weeks for absent suck as a sign of sepsis?	60%	Exclude
	Reduced movement of limbs	43%	Exclude
	Joint swelling	42%	Exclude
	Criteria for 'consider meningitis'	42%	Second round
Management	Do you agree with the antibiotic doses?	93%	Include
	Do you agree with the specified sepsis investigations if possible?	83%	Include
	Antibiotic duration for symptomatic sepsis = 7-10 days?	83%	Include (change to stop at day 7 if clinically well)
	Do you agree with the antibiotic choices if no local recommendation?	80%	Include (add WHO choices)
	Antibiotic duration for asymptomatic sepsis = 5 days?	66%	Include for Malawi, exclude for international
**Birth Asphyxia Diagnosis**	Resuscitation: BVM >5 mins / CPR >10 mins	94%	Include
	Foetal distress	86%	Include, MCTW
	Apgar at 5 mins <7	78%	Include as per COIN
	Vaginal breech	76%	Exclude
	Prolonged second stage >3 hrs duration	73%	Exclude
	Vacuum delivery	69%	Exclude
	Emergency caesarean section	61%	Exclude
	Birth injury	44%	Second round
Signs/ symptoms	Convulsions	93%	Include
	Coma	89%	Include
	Lethargy	84%	Include
	Hypotonia and gestation >34/40 weeks	80%	Include
	Irritable	77%	Exclude
	Absent suck and gestation >34/40 weeks	64%	Second round
	Should birth asphyxia be classified as mild, moderate or severe?	54%	Exclude
Additional RF?	Exclude the Moro reflex in LICs due to the difficulties of training HCW in checking safely?	80%	Include as part of Thompson score
	Poor feeding	49%	Exclude
	Respiratory distress	43%	Include as part of Thompson score
	Weight >4kg	43%	Exclude
Management	Give intravenous fluids if not tolerating oral or nasogastric feeds?	97%	Include

For peer review only

	No passive cooling for infants in LICs?	94%	Include
**RDN Diagnosis**	Do you agree with tachypnoea of >60 bpm for the other categories of RDN?	100%	Include
	Tachypnoea of 60-80 bpm <2 hrs old without signs or symptoms of sepsis should be treated as TTN and no antibiotics given?	76%	Exclude
	History of fast/laboured/noisy breathing is relevant as a sign or symptomatic of RDN when not present on admission?	44%	Exclude
RDN diagnostic criteria	Meconium aspiration syndrome	82%	Include
	Transient tachypnoea of the newborn	66%	Exclude
	Respiratory distress syndrome	63%	Second round
	Congenital pneumonia	63%	Second round
Management	Cut-off of 90% O_2 saturations before giving oxygen?	91%	Include
	Give antibiotics in all cases except TTN?	77%	Exclude
	Time cut-off of 2 hours for TTN? Would you have a higher or lower threshold?	54%	Exclude
**Hypothermia**	Diagnostic criteria for hypothermia?	100%	Include

BPM, breaths per minute; BVM, bag valve mask; CPR, cardiopulmonary resuscitation; HCW, heath care worker; LICs, low-income countries; MCTW, minor changes to wording; PROM, prolonged rupture of membranes; RDN, respiratory distress of the newborn; RF, risk factor; TTN, transient tachypnoea of the newborn; WHO, World Health Organisation

Key findings by algorithm

The first important finding was that the ‘major’ or ‘minor’ algorithmic weighting system (where one major risk factor for sepsis is equivalent to two minors) used to diagnose neonatal sepsis was near consensus (77%) but did not meet the 80% threshold. Experts called for further evidence before adopting this system: *“It is a difficult thing to do…you need to work out how specific and sensitive the app is by looking at blood cultures.”* Two experts suggested using a risk score calculator, and another two experts highlighted that WHO only uses danger signs. This weighting system was subsequently removed from the algorithm (Figure 2).

The second significant algorithmic finding was on HIE. An academic expert in neonatal encephalopathy discouraged the use of the term ‘birth asphyxia,’ a term used by Malawian HCWs and therefore incorporated into the original algorithm.

> *You really must not call it birth asphyxia because birth asphyxia means failing to breathe at birth and what you are talking about is encephalopathy.*

For peer review only

Additional feedback on the algorithm focused on the combination of risk factors or clinical signs and symptoms to consider or diagnose HIE. Experts cited a lack of evidence for using risk factors to diagnose birth asphyxia and that the application should only be using clinical signs and symptoms.

> *Birth asphyxia is not about risk factors. If you have encephalopathy it is a clinical diagnosis, and it is irrelevant what your risk factors are.*

Experts recommended utilizing a validated encephalopathy score,[19, 20] which was incorporated into the HIE algorithm. The risk factors that met consensus will be used as prompts to perform the Thompson score, which uses clinical signs and symptoms exclusively to diagnose HIE.

Third, for the respiratory algorithm, experts highlighted that "*it is hard to make an accurate diagnosis of a respiratory condition without investigations*." Therefore, the algorithm should focus instead on the management of respiratory distress. All respiratory conditions (respiratory distress syndrome, meconium aspiration, congenital pneumonia and transient tachypnoea of the newborn (TTN)) now fall under the umbrella diagnosis of respiratory distress of the newborn within the algorithm. For teaching purposes, the four respiratory conditions will be included as 'diagnoses to consider' in the management.

Finally, for the hypothermia algorithm, experts commented that first-line treatment for all newborns be skin-to-skin care including those who were severely hypothermic (<32°C) unless they showed any signs or symptoms of being unstable. Additionally, experts did not think it was realistic to review a newborn every 15-30 minutes when hypothermic.

Round Two

For peer review only - http://bmjopen.bmj.com/site/about/guidelines.xhtml

Ten (71%) experts completed round two, seven electronically and three by telephone interview. Four experts dropped out (three from HICs, one from LIC); three did not respond to email reminders and one expert was unable to meet the completion deadline. Nine items (41%) reached consensus (Figure 1). These items were either (1) included, unmodified (36%) or (2) included, modified in the revised algorithm (5%). Items that did not reach consensus (59%) were either (1) excluded (41%) or (2) included, modified according to WHO guidelines or expert suggestion (18%).

In round one experts indicated that hypothermia was a major sign of sepsis and should be included in the sepsis algorithm if persistent. In round two we clarified that the application is to be used at the time of admission onto the neonatal unit, at which point the HCW will only have one temperature reading. Experts in round two disagreed that a single temperature reading of $<35.5^{0}$C was a sign of sepsis and felt that it would more likely be due to environmental hypothermia, a common problem in LICs. Additionally, in round two it was established that experts were much more concerned with extremely premature <1500g neonates (88% consensus) compared to slightly premature 1500-2500g neonates (62% consensus) being at risk for neonatal sepsis. Central cyanosis was an addition to the second round as an expert suggestion to include all WHO danger signs; despite missing consensus (with 78% agreement), it was ultimately included in the final sepsis algorithm to comply with WHO guidelines for danger signs.

All of the respiratory items marginally missed consensus. All three items were still included in the final teaching algorithms with revisions in line with WHO diagnostic criteria. Modifications to the algorithms can be found in online supplementary file 2.

DISCUSSION

For peer review only

We report the use of a modified Delphi technique to review digital clinical pathway algorithms for four neonatal conditions managed by HCWs in LICs. Approximately two thirds (62%) of the original algorithm items were ultimately included for use in the NeoTree digital platform based on consensus expert opinion and national/international guidelines. The NeoTree team revised the algorithms based on this feedback. Expert discussion emphasised gaps in evidence in neonatal care in LICs, highlighting areas for future research.

Each algorithm had components that triggered debate amongst the experts. For neonatal sepsis, three points were discussed. First, experts called for further evidence before adopting a 'major' and 'minor' algorithmic weighting system to diagnose neonatal sepsis. In response the NeoTree research team are conducting a study in Zimbabwe and Malawi looking at which clinical indicators are predictors of positive blood cultures. Second, there was disparity in opinion regarding whether to give prophylactic antibiotics and the duration of antibiotics for newborns with risk factors for sepsis who remain clinically well without any supporting investigations (NeoTree's equivalence to asymptomatic sepsis). The WHO recommendation to administer prophylactic antibiotics for a neonate with maternal risk factors for sepsis only is considered weak with very low-quality evidence.[21] Despite reaching a consensus on particular risk factors (prolonged rupture of membranes, maternal fever), experts also highlighted the evidence base as weak. In terms of duration of treatment for asymptomatic sepsis, while expert opinion varied, the Malawian guidelines recommend a five-day course[11] whilst WHO recommends two days.[16] The NeoTree algorithms will therefore keep to local and international recommendations, but the NeoTree team will feed back to the Malawian COIN expert panel that consensus suggested five days is too long to treat newborns with sepsis risk factors only. Third, experts disputed the

For peer review only

treatment choice and duration for symptomatic neonatal sepsis; incidentally, WHO recommendations lack strong evidence or efficacy.[22]

For the HIE algorithm, the Thompson score was preferred because it is simpler to perform, less time consuming and better at predicting poor outcomes in moderate and severe HIE during the first hours of life compared to the Sarnat score at 24 hours.[19] The NeoTree research team suspected that measures such as examining for posturing and Moro reflex would be relatively complicated for frontline HCWs with minimal training to assess. However, neonatal experts' experience and previous studies in LICs[23] assured the team that the score is relatively straightforward to teach.

Several points of discussion also centred on the respiratory algorithms. First, experts noted that even with investigations in HICs respiratory conditions may be difficult to diagnose.[24] Second, despite experts' concerns about antibiotic overprescribing in LICs and the need to differentiate TTN from other respiratory conditions, they did not think this was currently feasible in LICs due to limitations in HCW capacity, resources and knowledge. Thus, experts agreed that all neonates with signs of respiratory distress should have respiratory support and antibiotics. A recent study justified the use of antibiotics for tachypnoea alone in a neonate in a resource-limited setting.[25] Third, experts recommended performing chest x-rays (if available) only if imaging would change management (e.g. a longer course of antibiotics for congenital pneumonia) or if the neonate was deteriorating.

With the proliferation of clinical digital platforms in HICs and LICs, there is growing concern with the quality and safety standards of their clinical guidance. Countries and organisations (including WHO) are now taking measures to ensure application developers fulfil a strict set of criteria to protect patients.[26] While the Delphi technique can establish expert consensus, it may

For peer review only

also strengthen the safety and quality standards of clinical algorithms. This technique has been widely used in developing paper-based neonatal clinical guidelines in HICs and LICs.[27-29] There are also studies that have used the Delphi technique to develop items used in m-health tools.[30-32] Our study is unique in the application of this technique to develop algorithms on a digital platform specific to neonatal care in low-resource settings.

This study has several limitations. The choice of using a modified two-step Delphi process meant that a final face-to-face meeting was not possible, which may have prevented some exchange of important information to clarify differences in expert opinion. However, this method allowed for the contributions of geographically dispersed experts, maintained their anonymity and prevented them from conforming to other experts. The recruitment of more experts from HICs (64%) compared to LICs (36%), despite originally inviting equal numbers to participate, could have contributed to expert panel bias. Dropouts from the first to the second round could have affected the consensus level and contributed to attrition bias.

Some factors may have contributed to selection bias. The Delphi process is time intensive, which could have meant that those clinicians who are busier with perhaps even more clinical expertise or those with limited internet access (mainly LICs) could not participate. Additionally, offering a financial incentive might have obtained a more equal representation of experts. Another drawback of the Delphi being a labour-intensive process was that a year elapsed between the two rounds. Experts may have forgotten the algorithms and items from the first round in the second round if they did not read the summary of results or refresh their knowledge of the algorithms. Experts reported that they found the layout of the second questionnaire confusing; a re-design contributed to delays.

For peer review only

This study used the Delphi technique to refine four clinical decision algorithms in a neonatal digital platform designed for HCWs in LICs to standardise and improve the quality of newborn care. The key to implementing the NeoTree algorithms in other LICs will be to demonstrate that clinical algorithms in a digital application versus paper-based guidelines can aid HCWs in making faster, more accurate diagnoses and provide better, more cost-effective treatment that will ultimately improve the quality of newborn care and reduce mortality. This will require a large-scale clinical-trial evaluation. Ultimately, with consensus opinion shaping the algorithms of this digital platform, accurate data capture, immediate clinical assessment and optimal medical care may be achieved to improve neonatal outcomes.

ACKNOWLEDGMENTS

We thank all the participants of the two rounds of Delphi surveys for their invaluable contributions to revise the clinical algorithms and highlight open questions. We would also like to thank Dr. Brian Corden and Dr. Alice Myers for their reviews of the manuscript.

CONTRIBUTORS

ME, CC and MH conceived the study, generated the methodology and designed the questionnaires. ME conducted the interviews and analysed the data. ME and MC produced the first draft and contributed equally to this manuscript. CC, FF and MH provided edits and comments to the draft. All authors reviewed and approved the final version of the manuscript.

FUNDING

This work was supported in part by the Wellcome Trust Digital Innovation Award (215742/z19/z). FF is supported by the Academy of Medical Sciences, the funders of the Starter Grant for Clinical Lecturers scheme and the UCL Great Ormond Street NIHR Biomedical

For peer review only

Research Centre. The funders had no role in the study design, data collection and analysis, or preparation of the manuscript.

COMPETING INTERESTS

None declared.

ETHICS APPROVAL

Ethics approval was not required for this study according to the University College of London Research Ethics Committee.

PROVENANCE AND PEER REVIEW

Not commissioned; externally peer reviewed.

DATA SHARING STATEMENT

All data relevant to the study are included in the article, uploaded as supplementary information or available upon request from the corresponding author.

PATIENT CONSENT FOR PUBLICATION

Not required.

ORCID IDs

Mari Evans https://orcid.org/0000-0001-5113-3629

Mark Corden https://orcid.org/0000-0001-6146-1634

Caroline Crehan https://orcid.org/0000-0002-3655-6954

Felicity Fitzgerald https://orcid.org/0000-0001-9594-3228

Michelle Heys https://orcid.org/0000-0002-1458-505X

For peer review only

REFERENCES

1 WHO. 2019. Newborns: reducing mortality. https://www.who.int/news-room/fact-sheets/detail/newborns-reducing-mortality [Accessed May 2020].

2 Lawn JE, Blencowe H, Oza S, et al. Every Newborn: progress, priorities, and potential beyond survival. *Lancet* 2014;384(9938):189–205.

3 WHO. 2019. Levels & trends in child mortality: report 2019. Estimates developed by the UN Inter-agency Group for Child Mortality Estimation. https://www.unicef.org/reports/levels-and-trends-child-mortality-report-2019 [Accessed May 2020].

4 WHO. 2015. WHO Guidelines Approved by the Guidelines Review Committee. https://www.who.int/publications/guidelines/guidelines_review_committee/en/ [Accessed June 2019].

5 WHO. 2011. MHealth: New horizons for health through mobile technologies. https://www.who.int/goe/publications/goe_mhealth_web.pdf [Accessed June 2019].

6 Mitchell M, Hedt-Gauthier BL, Msellemu D, et al. Using electronic technology to improve clinical care results from a before-after cluster trial to evaluate assessment and classification of sick children according to Integrated Management of Childhood Illness (IMCI) protocol in Tanzania. *BMC Med Inform Decis Mak* 2013;13:95.

7 Schaeffer LE, Ahmed S, Rahman M, et al. Development and evaluation of a mobile application for case management of small and sick newborns in Bangladesh. *BMC Med Inform Decis Mak* 2019;19:116.

8 Thairu L, Wirth M, Lunze K, et al. Innovative newborn health technology for resource-limited environments. *Trop Med Int Health* 2013;18:117–28.

For peer review only

9 Crehan C, Kesler E, Nambiar B, et al. The NeoTree application: Developing an integrated mHealth solution to improve quality of newborn care and survival in a district hospital in Malawi. *BMJ Glob Health* 2019;4:e000860.

10 WHO. 2013. Pocket book of hospital care for children: guidelines for the management of common childhood illnesses, 2nd ed. https://apps.who.int/iris/handle/10665/81170 [Accessed June 2018].

11 COIN Course, 2017. Care of the infant and newborn in Malawi. The COIN Course Participants Manual. Available from: http://cms.medcol.mw/cms_uploaded_resources/41905_12.pdf [Accessed June 2018].

12 Becker JU, Theodosis C, Jacob ST, et al. Surviving sepsis in low-income and middle-income countries: new directions for care and research. *Lancet Infect Dis* 2009;9(9):577–82.

13 Sinha IP, Smyth RL, Williamson PR. Using the Delphi technique to determine which outcomes to measure in clinical trials: recommendations for the future based on a systematic review of existing studies. *PLoS Med* 2011;8(1):e1000393.

14 Hsu CC, Sandford BA. The Delphi Technique: Making Sense of Consensus. *Practical Assessment, Research and Evaluation* 2007;12(10). https://scholarworks.umass.edu/pare/vol12/iss1/10 [Accessed July 2018].

15 Akins RB, Tolson H, Cole BR, et al. Stability of response characteristics of a Delphi panel: application of bootstrap data expansion. *BMC Med Res Methodol* 2005;5:37.

16 WHO. 2012. Recommendations for management of common childhood conditions: evidence for technical update of pocket book recommendations: newborn conditions, dysentery, pneumonia, oxygen use and delivery, common causes of fever, severe acute malnutrition and supportive care.

For peer review only

https://apps.who.int/iris/bitstream/handle/10665/44774/9789241502825_eng.pdf?sequence=1 [Accessed June 2019].

17 European Medicines Agency. 2010. Report on the Expert Meeting on Neonatal and Paediatric Sepsis. https://www.ema.europa.eu/en/documents/report/report-expert-meeting-neonatal-paediatric-sepsis_en.pdf [Accessed July 2019].

18 Babakus E, Mangold WG. Adapting the SERVQUAL scale to hospital services: an empirical investigation. *Health Serv Res* 1992;26(6):767–786.

19 Thompson CM, Puterman AS, Linley LL, et al. The value of a scoring system for hypoxic ischaemic encephalopathy in predicting neurodevelopmental outcome. *Acta Paediatr* 1997;86(7):757–761.

20 Sarnat HB, Sarnat MS. Neonatal encephalopathy following fetal distress: a clinical and electroencephalographic study. *Arch Neurol* 1976;33(10):696–705.

21 Ungerer RL, Lincetto O, McGuire W, et al. Prophylactic versus selective antibiotics for term newborn infants of mothers with risk factors for neonatal infection. *Cochrane Database Syst Rev* 2004;4:CD003957.

22 Obiero CW, Seale AC, Berkley JA. Empiric treatment of neonatal sepsis in developing countries. *Pediatr Infect Dis J* 2015;34(6):659–661.

23 Bhagwani DK, Sharma M, Dolker S, et al. To study the correlation of Thompson scoring in predicting early neonatal outcome in post asphyxiated term neonates. *J Clin Diagn Res* 2016;10(11):SC16-SC19.

24 Costa S, Rocha G, Leitao A, et al. Transient tachypnea of the newborn and congenital pneumonia: a comparative study. *J Matern Fetal Neonatal Med* 2012;25(7):992–4.

For peer review only

25 Tikmani SS, Muhammad AA, Shafiq Y, et al. Ambulatory treatment of fast breathing in young infants aged <60 days: A double-blind, randomized, placebo-controlled equivalence trial in low-income settlements of Karachi. *Clin Infect Dis* 2017;64(2):184–189.

26 van Haasteren A, Gille F, Fadda M, et al. Development of the mHealth App Trustworthiness checklist. *Digit Health* 2019;5:1–21

27 Kamath-Rayne BD, Thukral A, Visick MK, et al. Helping Babies Breathe, Second Edition: A Model for Strengthening Educational Programs to Increase Global Newborn Survival. *Glob Health Sci Pract* 2018;6:538–551.

28 Balikuddembe MS, Tumwesigye NN, Wakholi PK, et al. Expert perspectives on essential parameters to monitor during childbirth in low resource settings: a Delphi study in sub-Saharan Africa. *Reprod Health* 2019;16(1):119.

29 Housseine N, Punt MC, Browne JL, et al. Delphi consensus statement on intrapartum fetal monitoring in low-resource settings. *Int J Gynaecol Obstet* 2018;146(1):8–16.

30 Bakibinga P, Kamande E, Omuya M, et al. The role of a decision-support smartphone application in enhancing community health volunteers' effectiveness to improve maternal and newborn outcomes in Nairobi, Kenya: quasi-experimental research protocol. *BMJ Open* 2017;207(7):e014896.

31 Fung JST, Akech S, Kissoon N, et al. Determining predictors of sepsis at triage among children under 5 years of age in resource-limited settings: A modified Delphi process. *PLoS One* 2019;14(1):e0211274.

32 Pellé KG, Rambaud-Althaus C, D'Acremont V, et al. Electronic clinical decision support algorithms incorporating point-of-care diagnostic tests in low-resource settings: a target product profile. *BMJ Glob Health* 2020;5:e002067.

For peer review only

Figure 1. Outcome of algorithm items after round one and round two of Delphi technique.

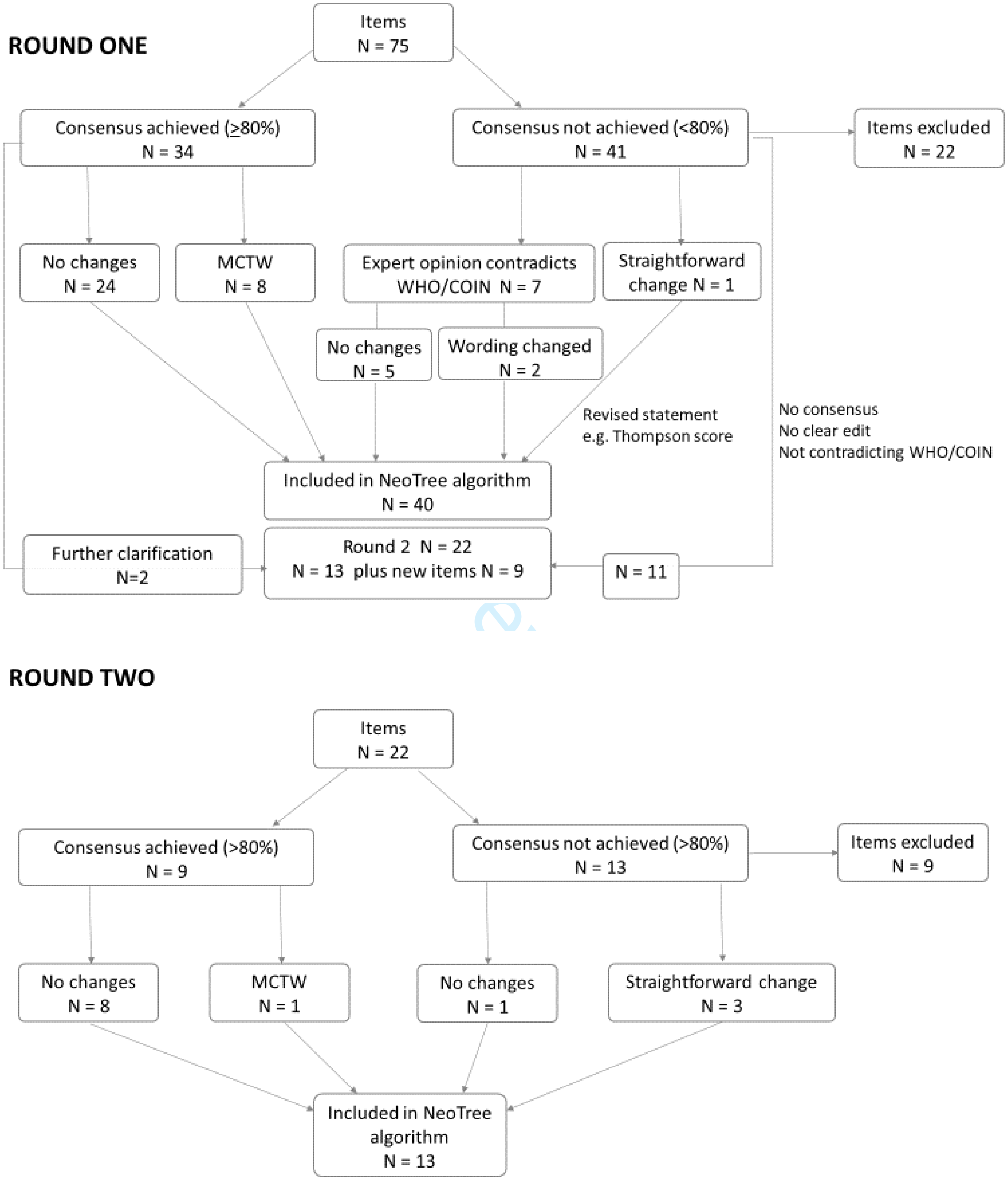

Abbreviations: COIN = Care of the Infant and Newborn in Malawi; MCTW = minor changes to wording; WHO = World Health Organisation

Figure 2. Modification of neonatal sepsis algorithm as a result of Delphi technique.

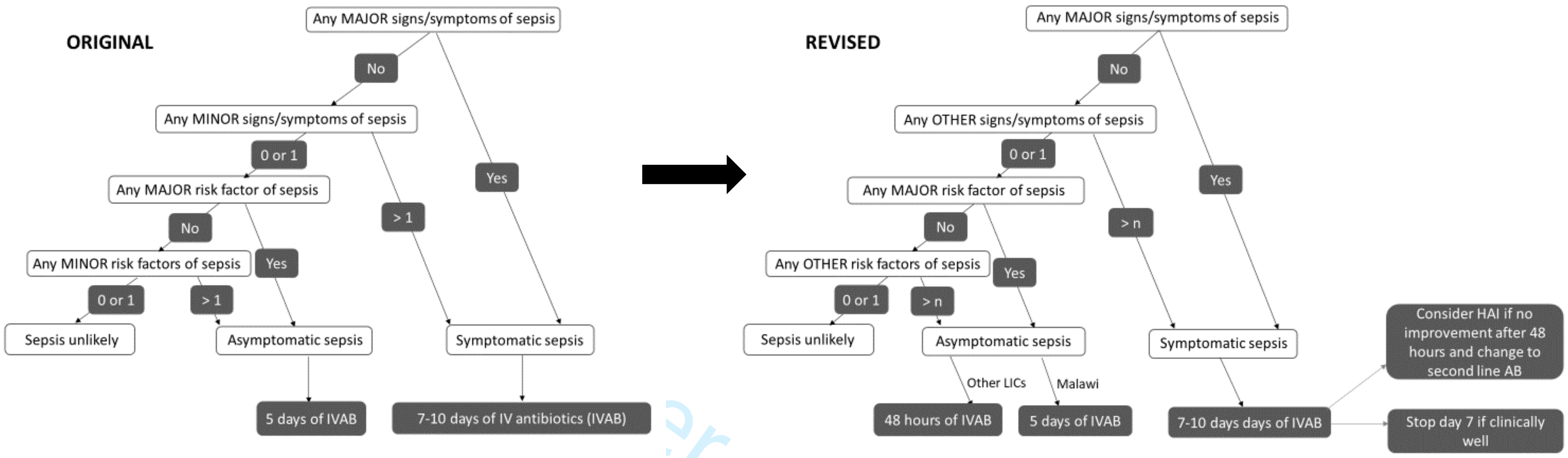

Abbreviations: AB = antibiotics; HAI = hospital-acquired infection; IVAB = intravenous antibiotics; LICs = low-income countries; > n = number of risk factors to be determined

Neonatal Sepsis Algorithm

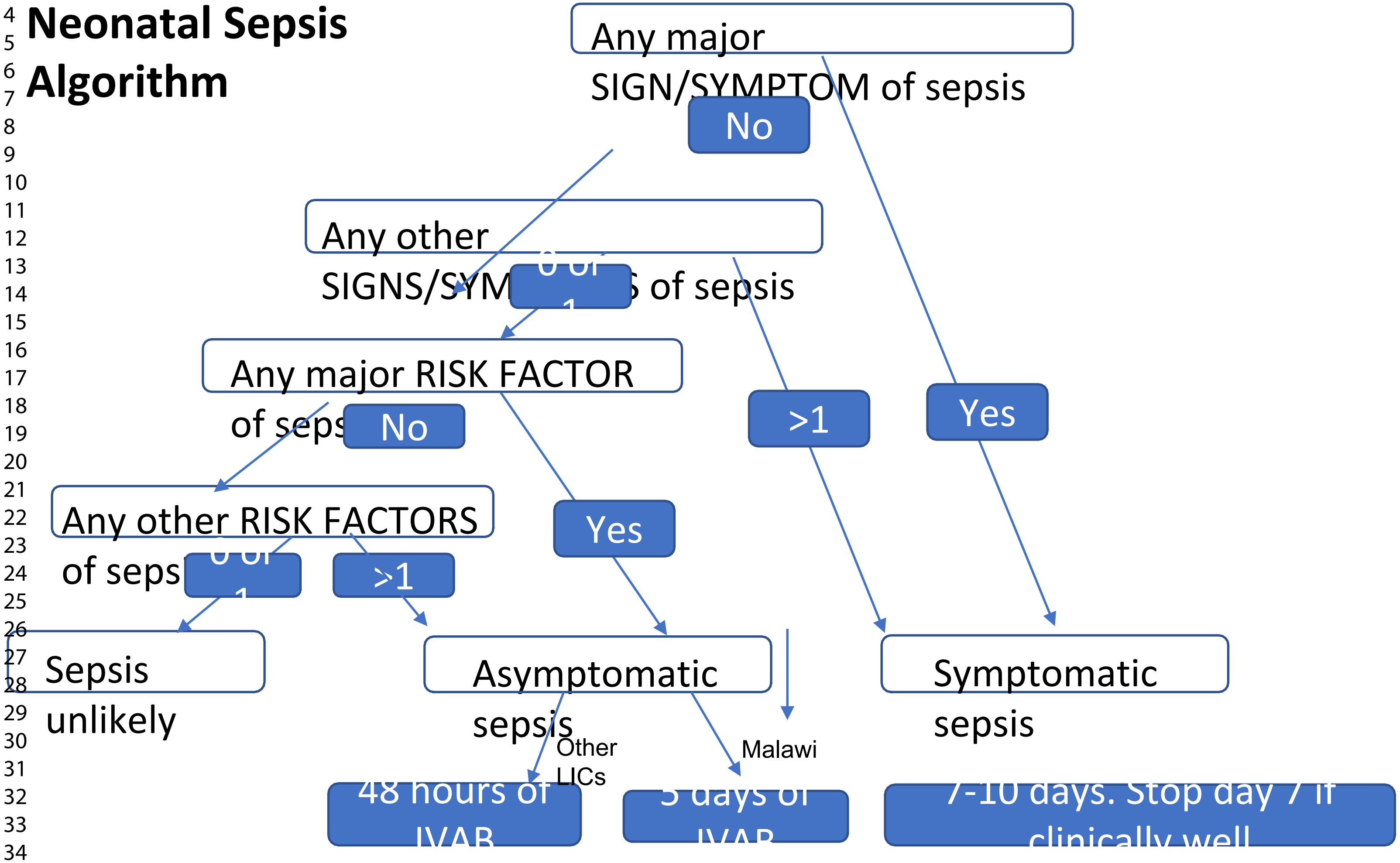

Neonatal Sepsis Algorithm

Risk factors

Major

- Maternal fever >38°C in labour
- Prolonged rupture of membranes (PROM) >18 hours
- Foul smelling amniotic fluid

Other risk factors

- <32/40 weeks gestation or <1500g (spontaneous labour only)

Signs/symptoms

Major

- Neonatal temperature >37.5 °C
- Boil/abscess
- Grunting/severe respiratory distress/ mod-severe WOB
- Lethargy
- Umbilical redness extending to the periumbilical skin or umbilicus draining pus
- Deep jaundice: palms and soles of the baby deep yellow
- Tachypnoea > 60 bpm
- Convulsions/twitching or abnormal movements
- Many or severe skin pustules
- Bilious vomiting with severe abdominal distension
- Bulging fontanelle
- New onset of poor feeding
- Not moving when stimulated
- Swollen red eyelids with pus
- Central cyanosis

Other signs/symptoms

- Pallor

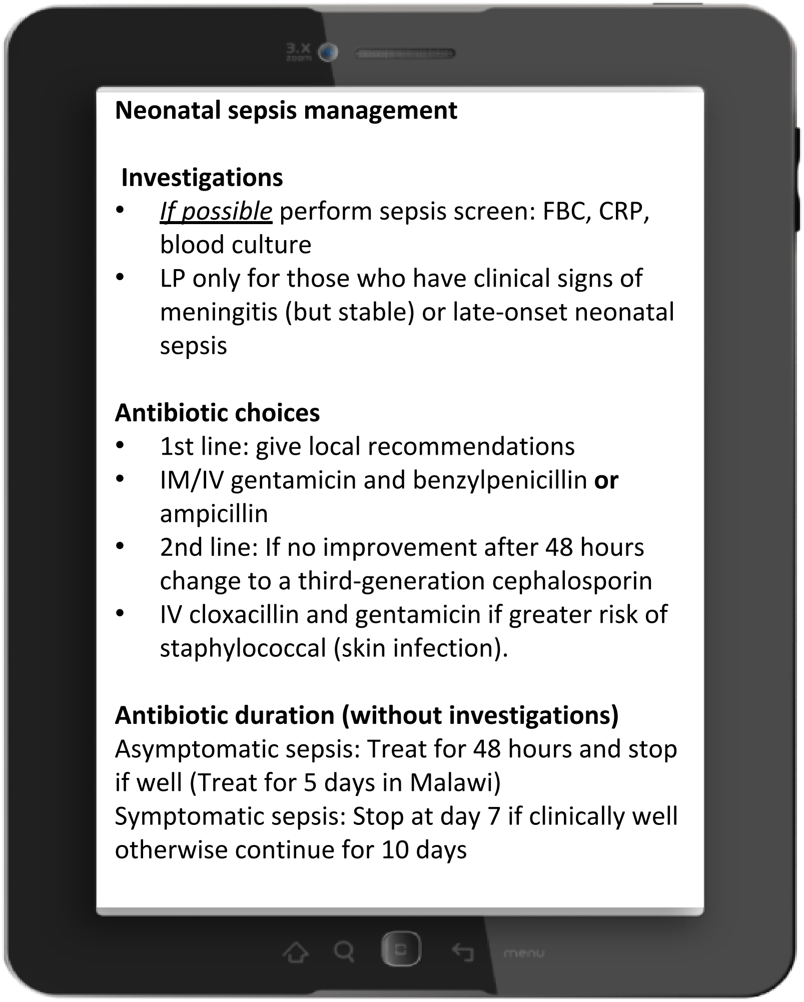
Neonatal sepsis management
Investigations
• If possible perform sepsis screen: FBC, CRP, blood culture
• LP only for those who have clinical signs of meningitis (but stable) or late-onset neonatal sepsis
Antibiotic choices
• 1st line: give local recommendations
• IM/IV gentamicin and benzylpenicillin or ampicillin
• 2nd line: If no improvement after 48 hours change to a third-generation cephalosporin
• IV cloxacillin and gentamicin if greater risk of staphylococcal (skin infection).
Antibiotic duration (without investigations)
Asymptomatic sepsis: Treat for 48 hours and stop if well (Treat for 5 days in Malawi)
Symptomatic sepsis: Stop at day 7 if clinically well otherwise continue for 10 days

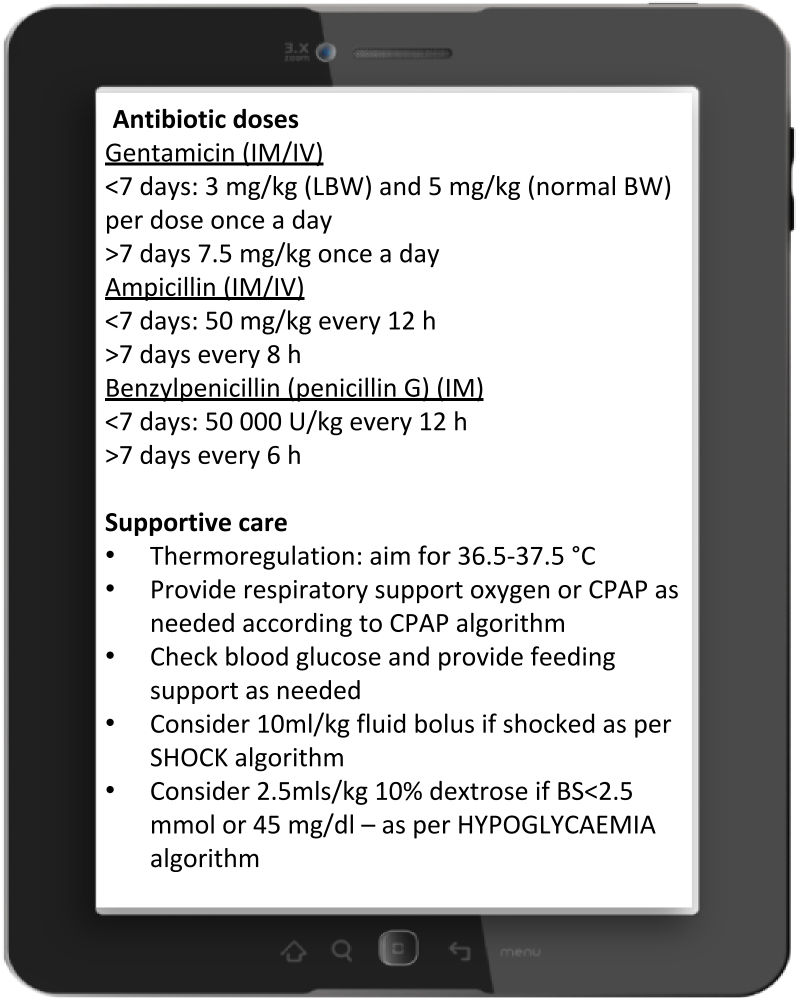
Antibiotic doses
Gentamicin (IM/IV)
<7 days: 3 mg/kg (LBW) and 5 mg/kg (normal BW) per dose once a day
>7 days 7.5 mg/kg once a day
Ampicillin (IM/IV)
<7 days: 50 mg/kg every 12 h
>7 days every 8 h
Benzylpenicillin (penicillin G) (IM)
<7 days: 50 000 U/kg every 12 h
>7 days every 6 h
Supportive care
• Thermoregulation: aim for 36.5-37.5 °C
• Provide respiratory support oxygen or CPAP as needed according to CPAP algorithm
• Check blood glucose and provide feeding support as needed
• Consider 10ml/kg fluid bolus if shocked as per SHOCK algorithm
• Consider 2.5mls/kg 10% dextrose if BS<2.5 mmol or 45 mg/dl – as per HYPOGLYCAEMIA algorithm

Hypoxic Ischaemic Encephalopathy Algorithm

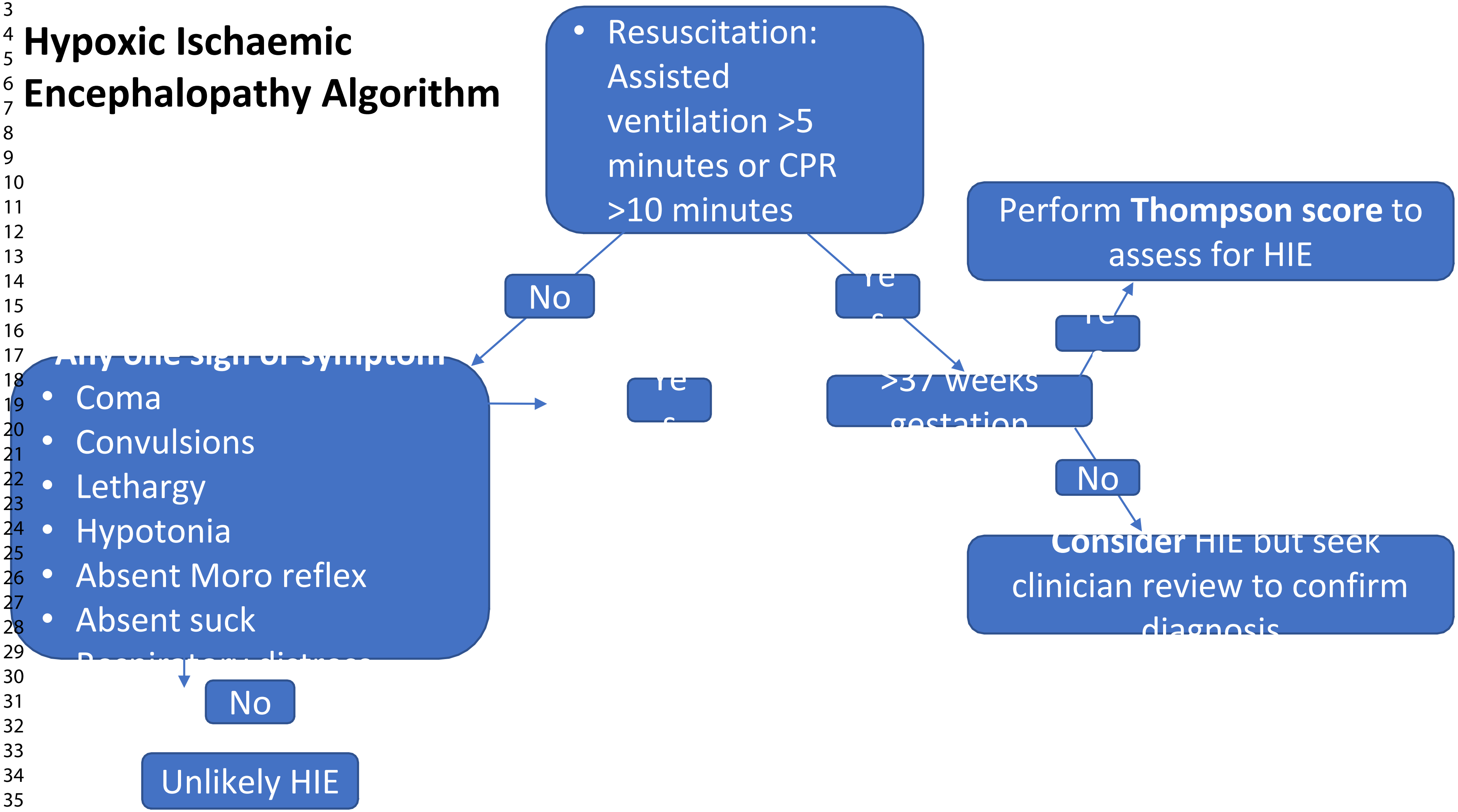

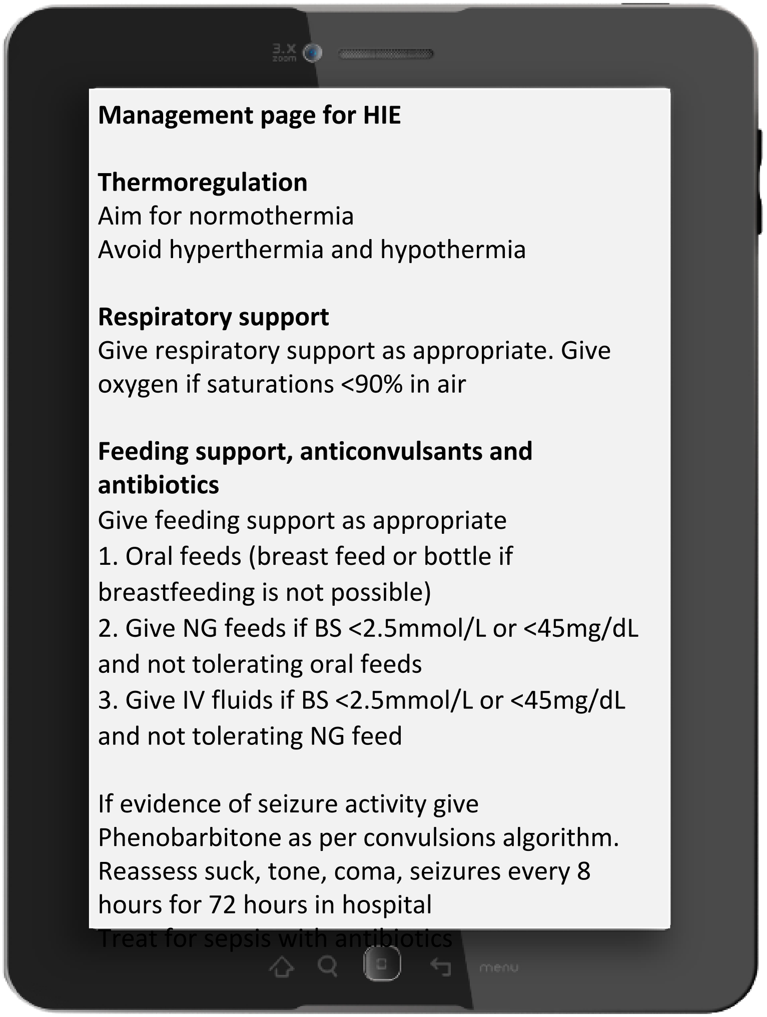

Management page for HIE

Thermoregulation
Aim for normothermia
Avoid hyperthermia and hypothermia

Respiratory support
Give respiratory support as appropriate. Give oxygen if saturations <90% in air

Feeding support, anticonvulsants and antibiotics
Give feeding support as appropriate
1. Oral feeds (breast feed or bottle if breastfeeding is not possible)
2. Give NG feeds if BS <2.5mmol/L or <45mg/dL and not tolerating oral feeds
3. Give IV fluids if BS <2.5mmol/L or <45mg/dL and not tolerating NG feed

If evidence of seizure activity give Phenobarbitone as per convulsions algorithm.
Reassess suck, tone, coma, seizures every 8 hours for 72 hours in hospital
Treat for sepsis with antibiotics

Respiratory Distress Algorithm

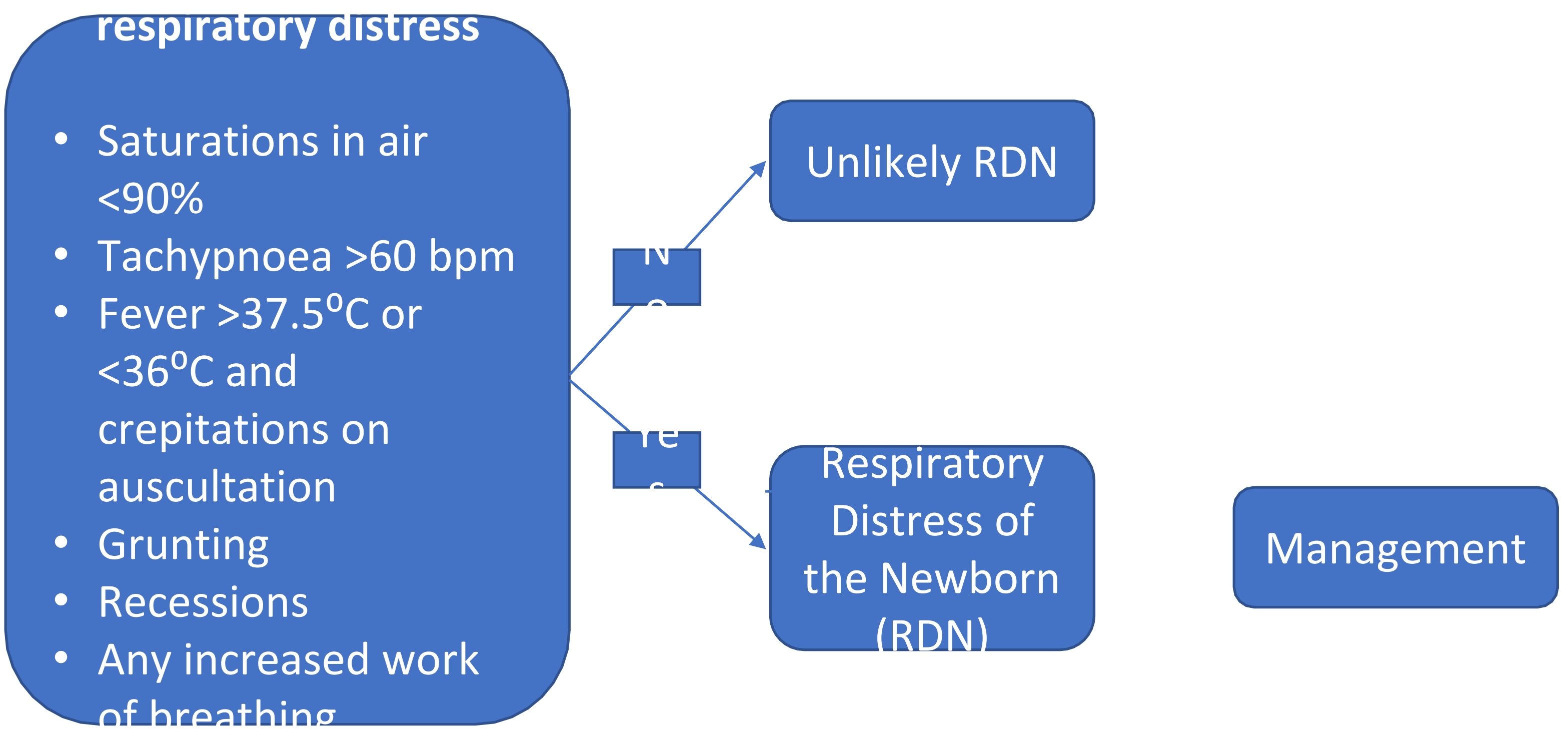

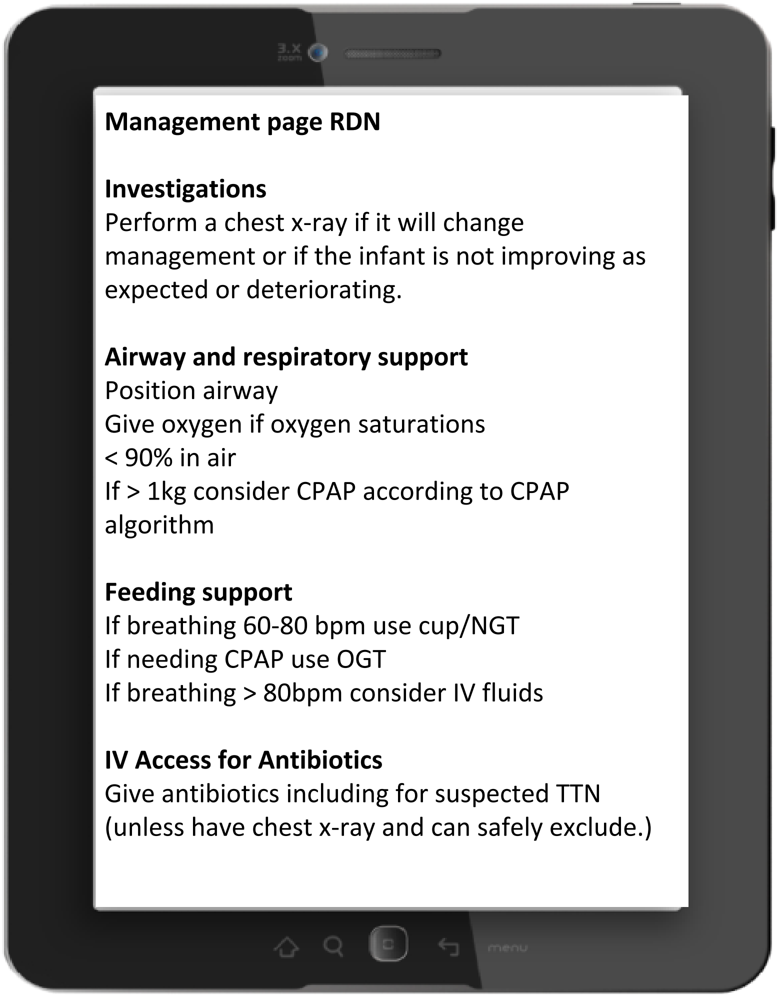
Management page RDN
Investigations
Perform a chest x-ray if it will change management or if the infant is not improving as expected or deteriorating.
Airway and respiratory support
Position airway
Give oxygen if oxygen saturations
< 90% in air
If > 1kg consider CPAP according to CPAP algorithm
Feeding support
If breathing 60-80 bpm use cup/NGT
If needing CPAP use OGT
If breathing > 80bpm consider IV fluids
IV Access for Antibiotics
Give antibiotics including for suspected TTN
(unless have chest x-ray and can safely exclude.)

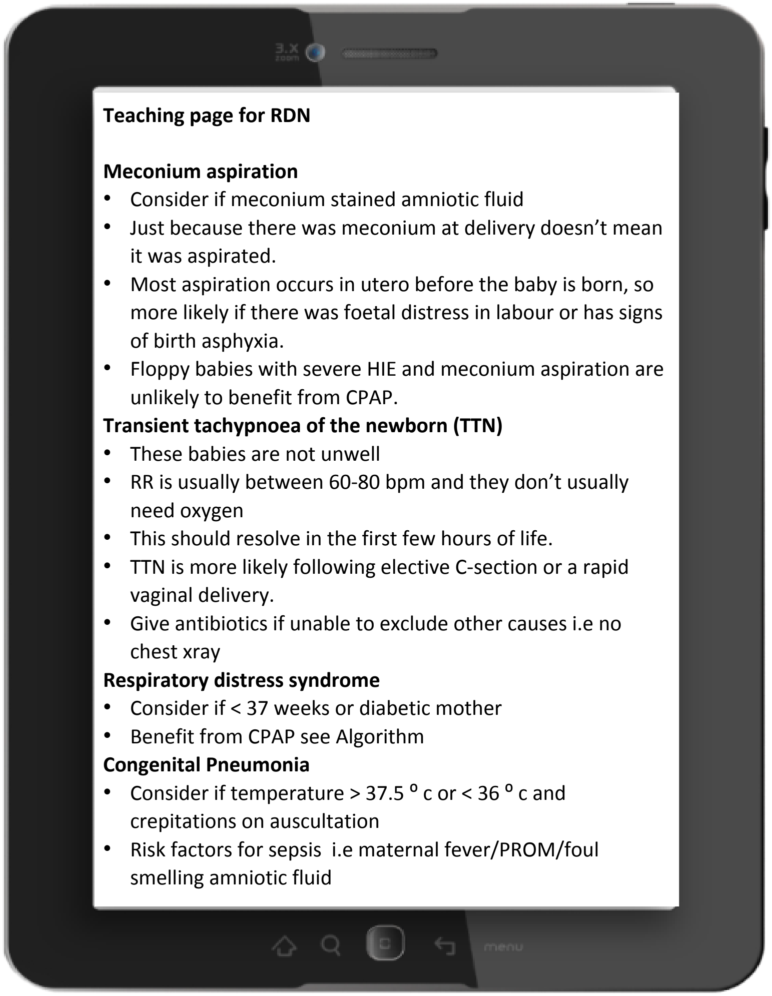
Teaching page for RDN
Meconium aspiration
Consider if meconium stained amniotic fluid
Just because there was meconium at delivery doesn't mean it was aspirated.
Most aspiration occurs in utero before the baby is born, so more likely if there was foetal distress in labour or has signs of birth asphyxia.
Floppy babies with severe HIE and meconium aspiration are unlikely to benefit from CPAP.
Transient tachypnoea of the newborn (TTN)
These babies are not unwell
RR is usually between 60-80 bpm and they don't usually need oxygen
This should resolve in the first few hours of life.
TTN is more likely following elective C-section or a rapid vaginal delivery.
Give antibiotics if unable to exclude other causes i.e no chest xray
Respiratory distress syndrome
Consider if < 37 weeks or diabetic mother
Benefit from CPAP see Algorithm
Congenital Pneumonia
Consider if temperature > 37.5 ° c or < 36 ° c and crepitations on auscultation
Risk factors for sepsis i.e maternal fever/PROM/foul smelling amniotic fluid

Hypothermia Algorithm

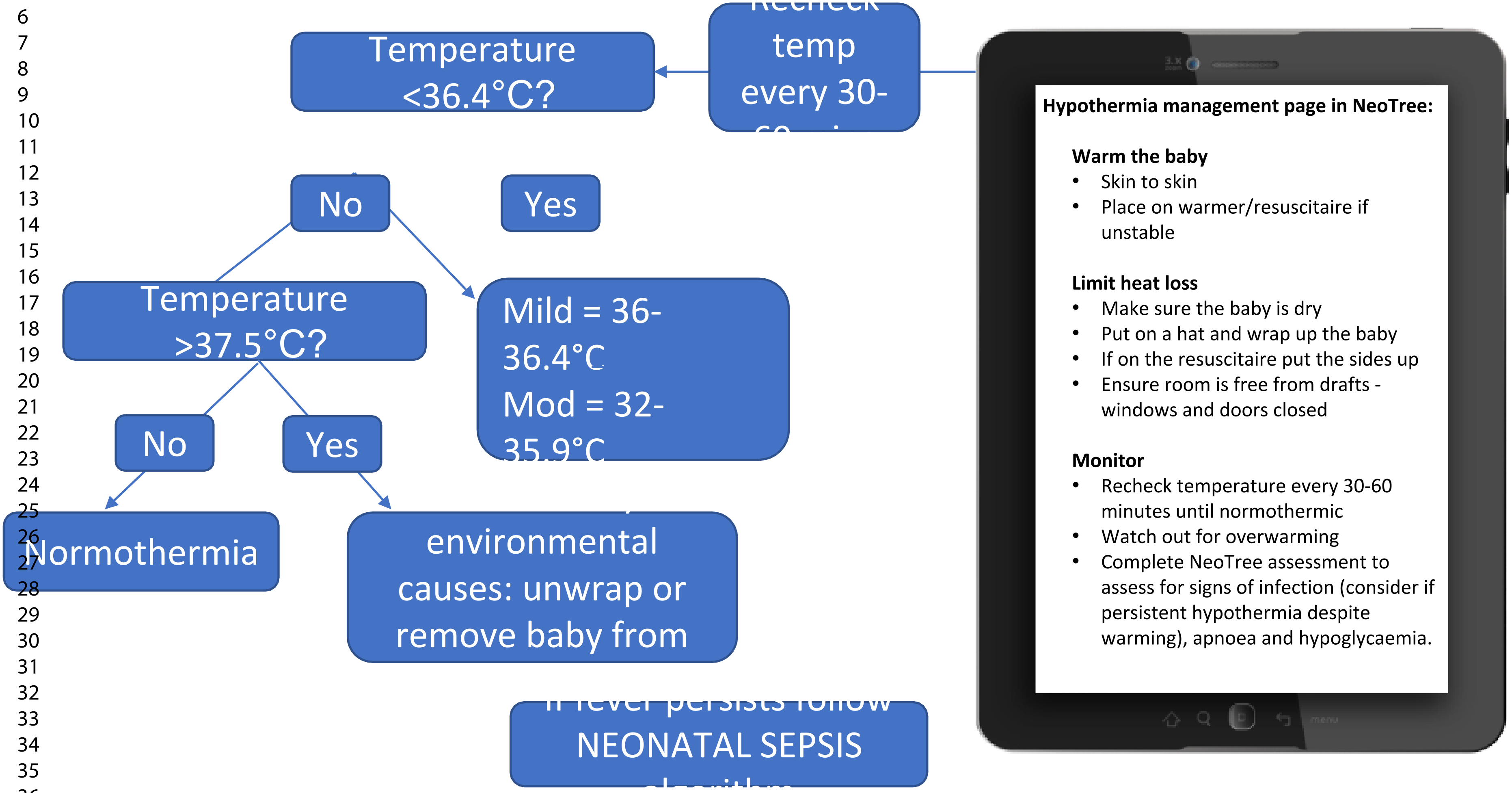

BMJ Open

Refining clinical algorithms for a neonatal digital platform for low-income countries: A modified Delphi technique

Journal:	*BMJ Open*
Manuscript ID	bmjopen-2020-042124.R1
Article Type:	Original research
Date Submitted by the Author:	28-Feb-2021
Complete List of Authors:	Evans, Mari; Great Ormond Street Hospital for Children, UCL Institute of Child Health<br>Corden, Mark; Children's Hospital of Los Angeles, Division of Hospital Medicine, Department of Pediatrics; University of Southern California Keck School of Medicine, Pediatrics<br>Crehan, Caroline; Great Ormond Street Hospital for Children, UCL Institute of Child Health<br>Fitzgerald, Felicity; Great Ormond Street Hospital for Children, UCL Institute of Child Health<br>Heys, Michelle; Great Ormond Street Hospital for Children, UCL Institute of Child Health
**Primary Subject Heading**:	Global health
Secondary Subject Heading:	Paediatrics, Health informatics
Keywords:	PUBLIC HEALTH, Information technology < BIOTECHNOLOGY & BIOINFORMATICS, Neonatal intensive & critical care < INTENSIVE & CRITICAL CARE, NEONATOLOGY

Note: The following files were submitted by the author for peer review, but cannot be converted to PDF. You must view these files (e.g. movies) online.
Online supp 1 - questionnaire.docx

SCHOLARONE™
Manuscripts

I, the Submitting Author has the right to grant and does grant on behalf of all authors of the Work (as defined in the below author licence), an exclusive licence and/or a non-exclusive licence for contributions from authors who are: i) UK Crown employees; ii) where BMJ has agreed a CC-BY licence shall apply, and/or iii) in accordance with the terms applicable for US Federal Government officers or employees acting as part of their official duties; on a worldwide, perpetual, irrevocable, royalty-free basis to BMJ Publishing Group Ltd ("BMJ") its licensees and where the relevant Journal is co-owned by BMJ to the co-owners of the Journal, to publish the Work in this journal and any other BMJ products and to exploit all rights, as set out in our licence.

The Submitting Author accepts and understands that any supply made under these terms is made by BMJ to the Submitting Author unless you are acting as an employee on behalf of your employer or a postgraduate student of an affiliated institution which is paying any applicable article publishing charge ("APC") for Open Access articles. Where the Submitting Author wishes to make the Work available on an Open Access basis (and intends to pay the relevant APC), the terms of reuse of such Open Access shall be governed by a Creative Commons licence – details of these licences and which Creative Commons licence will apply to this Work are set out in our licence referred to above.

Other than as permitted in any relevant BMJ Author's Self Archiving Policies, I confirm this Work has not been accepted for publication elsewhere, is not being considered for publication elsewhere and does not duplicate material already published. I confirm all authors consent to publication of this Work and authorise the granting of this licence.

For peer review only

Refining clinical algorithms for a neonatal digital platform for low-income countries: a modified Delphi technique

Mari Evans,[1*] Mark H. Corden,[2,3*] Caroline Crehan,[1] Felicity Fitzgerald, [1] Michelle Heys[1,4]

[1]Great Ormond Street Institute of Child Health, University College London, London, UK
[2]Division of Hospital Medicine, Department of Pediatrics, Children's Hospital Los Angeles, Los Angeles, CA, USA
[3]Department of Pediatrics, Keck School of Medicine, University of Southern California, Los Angeles, CA, USA
[4]Specialist Children's and Young People's Services, East London NHS Foundation Trust, London, UK

[*]denotes co-first authors

Corresponding Author:
Mari Evans
c/o Dr Michelle Heys
UCL Great Ormond Street Institute of Child Health
30 Guilford Street
London, WC1N 1EH
United Kingdom
evnsmari@hotmail.com

Word count: 4001

For peer review only

ABSTRACT

Objectives

To determine whether a panel of neonatal experts could address evidence gaps in local and international neonatal guidelines by reaching a consensus on four clinical decision algorithms for a neonatal digital platform (NeoTree).

Design

Two-round, modified Delphi technique.

Setting and participants

Participants were neonatal experts from high-income and low-income countries (LICs).

Methods

This was a consensus-generating study. In round one, experts rated items for four clinical algorithms (neonatal sepsis, hypoxic ischaemic encephalopathy, respiratory distress of the newborn, hypothermia) and justified their responses. Items meeting consensus (≥80% agreement) were included. Items not meeting consensus were either excluded, included following revisions or included if they contained core elements of evidence-based guidelines. In round two, experts rated items from round one that did not reach consensus.

Results

Fourteen experts participated in round one, ten in round two. Nine were from high-income countries, five from LICs. Experts included physicians and nurse practitioners with an average neonatal experience of 20 years, 12 in LICs. After two rounds, a consensus was reached on 43 of 84 items (52%). Despite not meeting consensus, additional items were included per experts' recommendations so that the practice of frontline healthcare workers continued to be in line with local and WHO guidelines. As a result, the final algorithms included 53 items (62%).

For peer review only

Conclusion

Four algorithms in a neonatal digital platform were reviewed and refined by consensus expert opinion. Revisions to NeoTree will be made in response to these findings. Next steps include clinical validation of the algorithms.

KEYWORDS

Information technology, neonatal intensive & critical care, neonatology, public health

For peer review only

STRENGTHS AND LIMITATIONS OF THIS STUDY

- In this study, a large number of algorithm items were reviewed and evaluated, and half met consensus for inclusion in the management pathways.
- The review was conducted with experts from a broad range of countries and neonatal experience who simultaneously refined the algorithms and highlighted gaps in current evidence, emphasising the need for future research to support international neonatal guidelines.
- Our study method meant that experts were not able to meet in person, which might have promoted dialogue that would have allowed greater clarity in their collective opinion.
- The representation of neonatal experts from LICs was not as robust as from high-income countries, which may have led to an uneven evaluation of the algorithms.

For peer review only

INTRODUCTION

Globally, 2.5 million newborns die each year in the first 28 days of life.[1] Most of these deaths (98.5%) occur in low-income countries (LICs), and 40% occur on the first day of life.[2] The neonatal mortality rate has been halved since 1990,[3] but modelling of global newborn mortality data suggests that a further two thirds of current deaths could be prevented if evidence-based solutions were implemented.[2] One of the World Health Organisation (WHO) Sustainable Development Goals (SDG3) is to end preventable deaths of newborns in all countries and to reduce the neonatal mortality rate from the current rate of 18 per 1000 live births to less than 12 per 1000 by 2030.[4] Targeting newborn care in LICs is thus an urgent priority, especially the three most common causes of mortality – infections (36%), prematurity (28%), and intrapartum complications (23%).[2]

The WHO neonatal guidelines are internationally recognised as the leading and most respected source of guidance.[5] However, one of their limitations is that they are primarily based on data from high-income countries as there is often a lack of evidence in LICs due to limited diagnostic aids, data and research.[6] The WHO aims to address the challenge of developing setting-appropriate neonatal guidelines by improving stakeholder involvement (design guidelines for specific audiences), clarity of presentation (often guidelines are too long and technical), and attention to dissemination.[7] Mobile health (m-health) technology and digital platforms are potential approaches to implementing these measures and improving the quality of newborn care.[8]

An international team of researchers, clinicians and software developers in the UK, USA, Malawi, Bangladesh and Zimbabwe co-designed and co-developed with Malawian and Zimbabwean HCWs a neonatal digital platform (NeoTree) for facility-based newborn care in

For peer review only

LICs. It combines immediate digital data capture (which is shared with HCWs via local dashboards), evidence-based algorithmic clinical decision and management support, newborn education and data linkage to national data systems on one platform.[9] The algorithms in the Malawian version of the NeoTree support decisions according to established international[10] and Malawian neonatal guidelines.[11] In situations where guidelines were not applicable, the NeoTree clinical team used clinical judgment to complete the algorithm development. In the absence of extensive trial or epidemiological data in LICs, alternative techniques to consolidate best available low-quality evidence can be used, such as expert opinion. This study aims to use the modified Delphi technique to determine whether a panel of experts in newborn care can reach a consensus opinion about key clinical decision algorithms used in a digital platform to assist HCWs caring for facility-based unwell newborns in LICs.

METHODS

Study design

This study used a two-step modified Delphi technique.[12] The Delphi technique was chosen because it is an effective method of gathering expert knowledge from geographically diverse leaders in the field to address complex clinical problems that lack evidence.

Recruitment

Twenty-two neonatal experts were invited to participate in the study. This number represented an adequate sample size[13, 14] and permitted a manageable amount of data collection. Participants were recruited if they were a physician or neonatal nurse practitioner with more than ten years neonatal experience (at least three in LICs), neonatal postgraduate training, fluency in English, internet access and willingness to participate. Neonatal experts known to the researchers for their clinical expertise, research and contributions to guideline development in LICs were identified in

For peer review only

equal numbers from both LICs and HICs. No financial incentive was offered, but reimbursement for costs of Skype calls was provided for some experts in LICs.

Algorithms and item generation

The four clinical decision algorithms selected for review were neonatal sepsis, hypoxic ischaemic encephalopathy (HIE), respiratory distress of the newborn and hypothermia. These conditions represent the leading preventable causes of neonatal mortality and are the most difficult to diagnose and manage appropriately in LICs with some of the weakest WHO GRADE recommendations and quality of evidence.[15] For example, the European definition of neonatal sepsis is two or more clinical symptoms and two or more laboratory signs in the presence of, or as a result of, suspected or proven infection.[16] This definition is not possible in LICs where laboratory investigations are not routinely available.[17]

Items were identified by comparing the algorithms side-by-side with the international (WHO) and local neonatal guidelines (Care of the Infant and Newborn in Malawi - COIN) from which they had been derived. This comparison generated a comprehensive list of items where discrepancies in diagnostic parameters and treatment recommendations required expert opinion. Once finalised, the clinical algorithms and list of items (henceforth referred to as questionnaire) were piloted with two paediatricians with neonatal experience in LICs. Ambiguous items were amended accordingly.

Delphi technique

The questionnaire was circulated by email to the experts with specific instructions at least two weeks before they were interviewed. Each algorithm was verbally and diagrammatically explained with their references specified (i.e., WHO, COIN or NeoTree research team) to aid in decision-making during the interview (online supplemental file 1). Round one interviews were

For peer review only

conducted in June and July 2018. Experts were sent up to two reminder emails to schedule their phone or Skype interview. Interviews were conducted privately from a home office. Standardised questions were used to review each item from the questionnaire. Experts were asked to rate their level of agreement for including an item in an algorithm using a five-point Likert scale. A five-point scale was chosen because evidence suggests that a five-point scale appears to be less confusing and to increase expert response rate.[18] Each rating was followed by open-ended questions to obtain the experts' rationale for their response and any amendment or additional items they would propose. All interview data were transcribed using both audio-recordings and notes made during the interview by the facilitator. All responses were anonymised (with participant numbers) and reviewed together with the quantitative results.

The upper limit of agreement amongst experts has been recommended to be set at 80% (4 or higher on the Likert scale) for Delphi studies.[13] Due to our sample size, this upper limit was used to apply greater rigor to item inclusion. Items that met consensus (≥80% agreement) were included or were modified with minor changes to wording based on expert advice. Items that did not meet consensus (<80%) were removed or modified according to the feedback from the expert panel and submitted for the second round. Items that did not meet consensus were still included if they were part of WHO and COIN guidelines so that frontline HCWs continued to follow the current standard of care. A second questionnaire was designed with modified items and expert additions from the first round (e.g., inclusion of the Thompson encephalopathy score[19]).

In round two (June and July 2019), this second questionnaire was distributed electronically to the 14 experts from round one (online supplemental file 1). A results summary from round one was sent to the experts, and the full set of anonymised results were made available at their request. Two email reminders were sent to non-responders. Experts again rated items on a Likert scale

For peer review only

and explained their ratings. Responses were analysed as described in round one, and items meeting ≧80% consensus were kept for the final NeoTree algorithms.

Patient and public involvement

While key stakeholders were involved in co-developing the NeoTree digital platform, there was no patient or public involvement in this Delphi study.

Consent procedures

The goals and processes of the project were explained to the experts in their email invitation, and consent was obtained by email agreement. Experts were verbally informed at the beginning of the first round that their responses would be kept anonymous.

RESULTS

Twenty-two neonatal experts were invited to participate. Sixteen responded; one declined due to lack of financial incentive, and one declined due to conflict of interest. All respondents had work experience in Africa; one respondent had over 20 years of clinical experience in Malawi and contributed to the development of the COIN guidelines. Demographics of the expert panel are listed in Table 1.

Table 1. Characteristics of the Delphi panel from round one.

	**Characteristics**	**N = 14 (%)**
Location	Experts from HICs	9 (64)
	Experts from LICs	5 (36)
Level of expertise	Neonatologist	6 (43)
	Paediatrician	6 (43)
	ANNP	2 (14)
Years of neonatal experience post medical school (mean ± SD)	Overall	20 (± 12)
	In LICs	12 (± 7)
Work experience in LICs	Africa	14 (100)
	Asia	7 (50)
	Central America	4 (28)
Country of medical degree	United Kingdom	7

For peer review only

United States	2
South Africa	2
Rwanda	1
Sudan	1
Zimbabwe	1

ANNP, advanced neonatal nurse practitioner; HICs, high-income countries; LICs, low-income countries

Round One

Fourteen experts (63% response rate) completed round one. Interviews averaged 73 minutes (40-110 minutes). Thirty-four items (45%) reached consensus (Figure 1). These items were either: (1) included, unmodified (32%); (2) included, modified (11%); or (3) changed for clarification in the second round (2%). Items that did not reach consensus (55%) were either: (1) excluded from the revised algorithm (30%); (2) included because they were part of WHO/COIN guidelines or the Thompson score (11%); or (3) changed and submitted for a second round (14%) (Table 2). The expert panel consistently stated that algorithm items must comply with WHO danger signs and COIN guidelines for neonatal sepsis, irrespective of whether the panel agreed with them. For example, experts thought that 'bulging fontanelle' was "*subjective; there are non-infectious causes … many babies' fontanelles bulge when they just cry.*" Another item that did not meet consensus was 'poor feeding,' which experts found vague for multiple reasons, including*: "it is too subjective; it depends on how long for … many newborns do not feed well on the first day of life.*" However, experts agreed that poor feeding was a sign of possible sepsis if it was *"a new onset of poor feeding when the infant had previously been feeding well."* This item was changed to 'new onset of poor feeding' for the final algorithm.

Two items that were included because they are part of the COIN guidelines highlighted inconsistencies with WHO guidelines. For example, COIN uses a temperature of more than 37.5°C as a fever for a newborn, whilst WHO and most experts use more than 38°C. Therefore,

37.5°C was included for the Malawian digital platform, but 38°C will be used for other countries. Other items where a difference between the recommendations and the guidelines occurred were antibiotic choice and duration for neonatal sepsis.

Modifications usually involved adopting the language utilized by WHO or COIN, but there were items that experts felt needed clarifying. For example, experts felt that *"twitching or abnormal movements"* needed to be added to the WHO term 'convulsions' because seizures in a neonate can be very subtle. Certain items that that could not be revised easily were submitted for the second round according to feedback from the expert panel. For example, experts disagreed that 'very/extremely premature (<32 weeks gestation)' was a major risk factor for sepsis if *"the baby was delivered as a clean cold caesarean section for maternal reasons and the mother was not in labour."* Eighty percent of experts highlighted that the algorithm should include weight to guide gestation because "*gestation is often unknown*" and "*you are relying on [the] Ballard score[20] which has plus or minus two weeks accuracy."* Similar opinions regarding method of delivery and the importance of birth weight were expressed for 'slightly premature (32-36 weeks gestation).' Both gestational age brackets were submitted into the second round as risk factors for sepsis after modifying the items to include WHO weight parameters to guide gestation.

Other items that did not gain consensus and were submitted for the second round included items that experts felt needed further clarification. 'Born before arrival' as a minor risk factor for sepsis was clarified to the experts that this meant the baby was born en-route to the hospital (either in a vehicle or on the roadside, both being considered dirty environments in Malawi). A 'neonate admitted with or history of a fever' as a minor risk factor for sepsis was changed to 'mother reports a non-measured fever' in the second round. Lastly, because experts considered the term

For peer review only

'birth injury' unclear, we asked them in round two to define what they considered a 'significant birth injury.'

Table 2. Round one heat chart to show which items met consensus and their outcomes.

Subject	Items	Agree	Outcome
**Sepsis Diagnosis** Major RF	Maternal fever >38°C in labour	91%	Include
	PROM > 18 hrs	74%	Include (WHO RF)
	Offensive smelling liquor	74%	Include (WHO RF); MCTW
	Very / extremely premature (<32/40 weeks)	74%	Second round
	Prolonged second stage (>3 hrs)	53%	Exclude
Minor RF	Prematurity (32-37 weeks gestation)	81%	Second round
	Born before arrival	70%	Second round
Major signs/ symptoms	Boil/abscess	93%	Include
	Grunting or severe respiratory distress or moderate-severe work of breathing	97%	Include
	Lethargy	93%	Include
	Red skin all around umbilicus	81%	Include, MCTW
	Jaundice <24 hrs old	83%	Include, MCTW
	Tachypnoea >60 bpm (>2 hrs old)	83%	Include, MCTW
	Convulsions	89%	Include, MCTW
	Pustules all over body	80%	Include, MCTW
	Bulging fontanelle	77%	Include (WHO danger sign)
	Temperature >37.5°C	73%	Include for Malawi version
	Admitted with or history of fever	68%	Second round
	History of apnoea	67%	Exclude
	Bilious vomiting	61%	Second round
Minor signs/ symptoms	Tachypnoea 60-80 bpm & <2 hrs old	85%	Include
	Pallor	81%	Include
	Weak or absent suck (AND >34/40 weeks)	73%	Exclude
	Poor feeding	73%	Include (WHO danger sign), MCTW
	Irritability	70%	Second round
	Distended abdomen	67%	Second round
	Heart rate >160 that cannot be explained by fever/crying	64%	Exclude
	Mild work of breathing	55%	Exclude
Additional RF?	Cut-off at 72 hrs for early vs late neonatal sepsis?	100%	Include
	Definition of maternal fever >38°C?	93%	Include
	Should PROM be >18 hrs or >24 hrs in LICs?	93%	Include >18 hrs
	Hypothermia < 35.5 °C	83%	Second round
	Fever in a newborn should be classified as >37.5°C in this setting?	83%	Include
	Please comment on our weighting system of Major = 100% / Minor = 50%	77%	Exclude
	Cut-off at >34/40 weeks for absent suck as a sign of sepsis?	60%	Exclude
	Reduced movement of limbs	43%	Exclude
	Joint swelling	42%	Exclude
	Criteria for 'consider meningitis'	42%	Second round
Management	Do you agree with the antibiotic doses?	93%	Include

	Do you agree with the specified sepsis investigations if possible?	83%	Include
	Antibiotic duration for symptomatic sepsis = 7-10 days?	83%	Include (change to stop at day 7 if clinically well)
	Do you agree with the antibiotic choices if no local recommendation?	80%	Include (add WHO choices)
	Antibiotic duration for asymptomatic sepsis = 5 days?	66%	Include for Malawi, exclude for international
**Birth Asphyxia Diagnosis**	Resuscitation: BVM >5 mins / CPR >10 mins	94%	Include
	Foetal distress	86%	Include, MCTW
	Apgar at 5 mins <7	78%	Include as per COIN
	Vaginal breech	76%	Exclude
	Prolonged second stage >3 hrs duration	73%	Exclude
	Vacuum delivery	69%	Exclude
	Emergency caesarean section	61%	Exclude
	Birth injury	44%	Second round
Signs/ symptoms	Convulsions	93%	Include
	Coma	89%	Include
	Lethargy	84%	Include
	Hypotonia and gestation >34/40 weeks	80%	Include
	Irritable	77%	Exclude
	Absent suck and gestation >34/40 weeks	64%	Second round
	Should birth asphyxia be classified as mild, moderate or severe?	54%	Exclude
Additional RF?	Exclude the Moro reflex in LICs due to the difficulties of training HCW in checking safely?	80%	Include as part of Thompson score
	Poor feeding	49%	Exclude
	Respiratory distress	43%	Include as part of Thompson score
	Weight >4kg	43%	Exclude
Management	Give intravenous fluids if not tolerating oral or nasogastric feeds?	97%	Include
	No passive cooling for infants in LICs?	94%	Include
**RDN Diagnosis**	Do you agree with tachypnoea of >60 bpm for the other categories of RDN?	100%	Include
	Tachypnoea of 60-80 bpm <2 hrs old without signs or symptoms of sepsis should be treated as TTN and no antibiotics given?	76%	Exclude
	History of fast/laboured/noisy breathing is relevant as a sign or symptomatic of RDN when not present on admission?	44%	Exclude
RDN diagnostic criteria	Meconium aspiration syndrome	82%	Include
	Transient tachypnoea of the newborn	66%	Exclude
	Respiratory distress syndrome	63%	Second round
	Congenital pneumonia	63%	Second round
Management	Cut-off of 90% O_2 saturations before giving oxygen?	91%	Include
	Give antibiotics in all cases except TTN?	77%	Exclude
	Time cut-off of 2 hours for TTN? Would you have a higher or lower threshold?	54%	Exclude
**Hypothermia**	Diagnostic criteria for hypothermia?	100%	Include

BPM, breaths per minute; BVM, bag valve mask; CPR, cardiopulmonary resuscitation; HCW, heath care worker; LICs, low-income countries; MCTW, minor changes to wording; PROM, prolonged rupture of membranes; RDN,

For peer review only

respiratory distress of the newborn; RF, risk factor; TTN, transient tachypnoea of the newborn; WHO, World Health Organisation

Key findings by algorithm

The first important finding was that the 'major' or 'minor' algorithmic weighting system (where one major risk factor for sepsis is equivalent to two minors) used to diagnose neonatal sepsis was near consensus (77%) but did not meet the 80% threshold. Experts called for further evidence before adopting this system: *"It is a difficult thing to do…you need to work out how specific and sensitive the app is by looking at blood cultures."* Two experts suggested using a sepsis risk score calculator, and another two experts highlighted that WHO only uses danger signs. This weighting system was subsequently removed from the algorithm.

The second significant algorithmic finding was on HIE. An academic expert in neonatal encephalopathy discouraged the use of the term 'birth asphyxia,' a term used by Malawian HCWs and therefore incorporated into the original algorithm.

> *You really must not call it birth asphyxia because birth asphyxia means failing to breathe at birth and what you are talking about is encephalopathy.*

Additional feedback on the algorithm focused on the combination of risk factors or clinical signs and symptoms to consider or diagnose HIE. Experts cited a lack of evidence for using risk factors to diagnose birth asphyxia and that the digital platform should only be using clinical signs and symptoms.

> *Birth asphyxia is not about risk factors. If you have encephalopathy, it is a clinical diagnosis, and it is irrelevant what your risk factors are.*

Experts recommended utilizing a validated encephalopathy score,[19, 21] which was incorporated into the HIE algorithm. The risk factors that met consensus may be used as prompts

For peer review only - http://bmjopen.bmj.com/site/about/guidelines.xhtml

to perform the Thompson score, which uses clinical signs and symptoms exclusively to diagnose HIE.

Third, for the respiratory algorithm, experts highlighted that "*It is hard to make an accurate diagnosis of a respiratory condition without investigations*." Therefore, the algorithm should focus instead on the management of respiratory distress. All respiratory conditions (respiratory distress syndrome, meconium aspiration, congenital pneumonia and transient tachypnoea of the newborn (TTN)) now fall under the umbrella diagnosis of respiratory distress of the newborn within the algorithm. For teaching purposes, the four respiratory conditions will be included as 'diagnoses to consider' in the management.

Finally, for the hypothermia algorithm, experts commented that first-line treatment for all newborns be skin-to-skin care including those who were severely hypothermic (<32°C) unless they showed any signs or symptoms of being unstable. Additionally, experts did not think it was realistic to review a newborn every 15-30 minutes when hypothermic. No major revisions were made to the hypothermia algorithm.

Round Two

Ten (71%) experts completed round two, seven electronically and three by telephone interview. Four experts dropped out (three from HICs, one from LIC); three did not respond to email reminders and one expert was unable to meet the completion deadline. Nine items (41%) reached consensus (Figure 1). These items were either (1) included, unmodified (36%) or (2) included, modified in the revised algorithm (5%). Items that did not reach consensus (59%) were either (1) excluded (41%) or (2) included, modified according to WHO guidelines or expert suggestion (18%) (Table 3).

For peer review only

In round one, experts indicated that hypothermia was a major sign of sepsis and should be included in the sepsis algorithm if persistent. In round two, we clarified that the digital platform is to be used at the time of admission onto the neonatal unit, at which point the HCW will only have one temperature reading. Experts in round two disagreed that a single temperature reading of <35.5^{0}C was a sign of sepsis and felt that it would more likely be due to environmental hypothermia, a common problem in LICs. Additionally, in round two, it was established that experts were much more concerned with extremely premature and/or <1500g neonates (88% consensus) compared to slightly premature and/or 1500-2500g neonates (62% consensus) being at risk for neonatal sepsis. Central cyanosis was an addition to the second round as an expert suggestion to include all WHO danger signs; despite missing consensus (with 78% agreement), it was ultimately included in the final sepsis algorithm to comply with WHO guidelines for danger signs.

All of the respiratory items marginally missed consensus. All three items were still included in the education sections of the digital platform with revisions in line with WHO diagnostic criteria. Modifications to the algorithms can be found in Figure 2 and online supplemental file 2.

Table 3. Round two heat chart to show which items met consensus and their outcomes.

Subject	Items	Agree	Outcome
**Sepsis Diagnosis** 'Other' RF	<32/40 weeks gestation and/or <1500g	82%	Include
	32-36/40 weeks gestation and/or 1500-2500g	62%	Exclude
	Babies born en-route to the hospital	54%	Exclude
	Mother reports a non-measured fever	52%	Exclude
Major sign	Bilious vomiting with severe abdominal distension	88%	Include
'Other' sign/ symptoms	Irritable/inconsolable baby	64%	Exclude
	Do you think a one-off T <35.5 °C should be added as a sign of neonatal sepsis in a LIC?	44%	Exclude
Additional RF?	Swollen red eyelids with pus	88%	Include
	Unconscious	86%	Include, MCTW
	Central cyanosis	78%	Include (WHO danger sign)
	Poor capillary refill or perfusion	68%	Exclude
	Does the baby look ill?	60%	Exclude
**Consider Meningitis**	Drowsy, lethargic or unconscious with T >37.5^{0}C	96%	Include
	Bulging fontanelle with T >37.5^{0}C	94%	Include
	Irritability with a high-pitched cry with T >37.5^{0}C	92%	Include

For peer review only

	Abnormal movements/twitching or convulsions with T >37.5^{0}C	90%	Include
	Abnormal tone with T >37.5^{0}C	80%	Include
**HIE diagnosis**	Do you agree with absent suck and gestation <32/40 weeks as a sign of HIE?	42%	Exclude
	How should we describe "significant" birth injury as a risk factor for HIE?	20%	Exclude
**RDN diagnosis**	Do you agree with gestation <34/40 for part of the diagnostic criteria for Respiratory Distress Syndrome?	74%	Include, change to WHO definition
	Do you agree with coarse crackles (instead of unilateral crackles) for part of the diagnostic criteria for congenital pneumonia?	74%	Include, change to WHO wording
	Do you agree with T >37.5^{0}C or <36.5^{0}C for part of the diagnostic criteria for congenital pneumonia?	70%	Include, change to expert suggestion

HIE, Hypoxic Ischaemic Encephalopathy; LICs, low-income countries; MCTW, minor changes to wording; RDN, respiratory distress of the newborn; RF, risk factor; T, temperature; WHO, World Health Organisation

DISCUSSION

We report the use of a modified Delphi technique to review digital clinical pathway algorithms for four neonatal conditions managed by HCWs in LICs. Approximately two thirds (62%) of the original algorithm items were ultimately included for use in the NeoTree digital platform based on consensus expert opinion and national/international guidelines. The NeoTree team revised the algorithms based on this feedback. Expert discussion emphasised gaps in evidence in neonatal care in LICs, highlighting areas for future research.

Each algorithm had components that triggered debate amongst the experts. For neonatal sepsis, three points were discussed. First, experts called for further evidence before adopting a 'major' and 'minor' algorithmic weighting system to diagnose neonatal sepsis. In response, the NeoTree research team are conducting a study in Zimbabwe and Malawi looking at which clinical indicators are predictors of positive blood cultures. Second, there was disparity in opinion regarding whether to give prophylactic antibiotics and the duration of antibiotics for newborns with risk factors for sepsis who remain clinically well without any supporting investigations (NeoTree's equivalence to asymptomatic sepsis). The WHO recommendation to administer prophylactic antibiotics for a neonate with maternal risk factors for sepsis is considered weak

For peer review only

with very low-quality evidence.[22] Despite reaching a consensus on particular risk factors (prolonged rupture of membranes, maternal fever), experts also highlighted the evidence base as weak. In terms of duration of treatment for asymptomatic sepsis, while expert opinion varied, the Malawian guidelines recommend a five-day course[11] whilst WHO recommends two days.[15] The NeoTree algorithms will therefore keep to local and international recommendations, but the NeoTree team will feed back to the Malawian COIN expert panel that consensus suggested five days is too long to treat newborns with sepsis risk factors only. Third, experts disputed the treatment choice and duration for symptomatic neonatal sepsis; incidentally, WHO recommendations lack strong evidence or efficacy.[17]

For the HIE algorithm, the Thompson score was preferred because it is simpler to perform, less time consuming and better at predicting poor outcomes in moderate and severe HIE during the first hours of life compared to the Sarnat score at 24 hours.[19] The NeoTree research team suspected that measures such as examining for posturing and Moro reflex would be relatively complicated for frontline HCWs with minimal training to assess. However, neonatal experts' experience and previous studies in LICs[23] assured the team that the score is relatively straightforward to teach.

Several points of discussion also centred on the respiratory algorithms. First, experts noted that even with investigations in HICs respiratory conditions may be difficult to diagnose.[24] Second, despite experts' concerns about antibiotic overprescribing in LICs and the need to differentiate TTN from other respiratory conditions, they did not think this was currently feasible in LICs due to limitations in HCW capacity, resources and knowledge. Thus, experts agreed that all neonates with signs of respiratory distress should have respiratory support and antibiotics. A recent study justified the use of antibiotics for tachypnoea alone in a neonate in a resource-limited setting.[25]

For peer review only

Third, experts recommended performing chest x-rays (if available) only if imaging would change management (e.g. a longer course of antibiotics for congenital pneumonia) or if the neonate was deteriorating.

With the proliferation of clinical digital platforms in HICs and LICs, there is growing concern with the quality and safety standards of their clinical guidance. Countries and organisations (including WHO) are now taking measures to ensure application developers fulfil a strict set of criteria to protect patients.[26] While the Delphi technique can establish expert consensus, it may also strengthen the safety and quality standards of clinical algorithms. This technique has been widely used in developing paper-based neonatal clinical guidelines in HICs and LICs.[27-29] There are also studies that have used the Delphi technique to develop items used in m-health tools.[30-32] Our study is unique in the application of this technique to develop algorithms on a digital platform specific to neonatal care in low-resource settings.

This study has several limitations. The choice of using a modified two-step Delphi process meant that a final face-to-face meeting was not possible, which may have prevented some exchange of important information to clarify differences in expert opinion. However, this method allowed for the contributions of geographically dispersed experts, maintained their anonymity and prevented them from conforming to other experts. The recruitment of more experts from HICs (64%) compared to LICs (36%), despite originally inviting equal numbers to participate, could have contributed to expert panel bias. We invited three Malawian clinicians as experts; the absence of their input could be another limitation, since the algorithms have initially been contextualized to the Malawian setting. However, the end goal of NeoTree development is to be applicable in a wide range of resource-limited settings; therefore, experts with a broad geographical range of

For peer review only

clinical experience were recruited. Dropouts from the first to the second round could have affected the consensus level and contributed to attrition bias.

Some factors may have contributed to selection bias. The Delphi process is time intensive, which could have meant that those clinicians who are busier with perhaps even more clinical expertise or those with limited internet access (mainly LICs) could not participate. Additionally, offering a financial incentive might have obtained a more equal representation of experts. Another drawback of the Delphi being a labour-intensive process was that a year elapsed between the two rounds. Experts may have forgotten the algorithms and items from the first round in the second round if they did not read the summary of results or refresh their knowledge of the algorithms. Experts reported that they found the layout of the second questionnaire confusing; a re-design contributed to delays.

This study used the Delphi technique to refine four clinical decision algorithms in a neonatal digital platform designed for HCWs in LICs to standardise and improve the quality of newborn care. The key to implementing the NeoTree algorithms in other LICs will be to demonstrate that clinical algorithms in a digital platform versus paper-based guidelines can aid HCWs in making faster, more accurate diagnoses and provide better, more cost-effective treatment that will ultimately improve the quality of newborn care and reduce mortality. This will require a large-scale clinical-trial evaluation. Ultimately, with consensus opinion shaping the algorithms of this digital platform, accurate data capture, immediate clinical assessment and optimal medical care may be achieved to improve neonatal outcomes.

ACKNOWLEDGEMENTS

We thank all the participants of the Delphi surveys for their invaluable contributions to revise the clinical algorithms and highlight open questions, including Dr. Helen Brotherton, Dr.

For peer review only

Simbarashe Chimhuya, Dr. Cath Harrison, Dr. Tyler Hartmann, Dr. Hammad Khan, Dr. Camilla Kindgon, Dr. Tom Lissauer, Dr. Azza Mashumba, Kathy Mellor (MBE), Professor Elizabeth Molyneux, Dr. Cally Tann, Dr. Cliff O'Callahan, Susan Quinton, and Dr. Raissa Teteli. We would also like to thank Dr. Brian Corden and Dr. Alice Myers for their reviews of the manuscript.

CONTRIBUTORS

ME, CC and MH conceived the study, generated the methodology and designed the questionnaires. ME conducted the interviews and analysed the data. ME and MC produced the first draft and contributed equally to this manuscript. CC, FF and MH provided edits and comments to the draft. All authors reviewed and approved the final version of the manuscript.

FUNDING

This work was supported in part by the Wellcome Trust Digital Innovation Award (215742/z19/z). FF is supported by the Academy of Medical Sciences, the funders of the Starter Grant for Clinical Lecturers scheme and the UCL Great Ormond Street NIHR Biomedical Research Centre. The funders had no role in the study design, data collection and analysis, or preparation of the manuscript.

COMPETING INTERESTS

CC and MH are co-founders of the NeoTree platform and continue to conduct research related to its development. FH and MH are trustees of the NeoTree Foundation. The NeoTree platform is a not-for-profit product; none of the co-authors benefit financially.

ETHICS APPROVAL

Ethics approval was not required for this study according to the University College of London Research Ethics Committee.

For peer review only

PROVENANCE AND PEER REVIEW

Not commissioned; externally peer reviewed.

DATA SHARING STATEMENT

All data relevant to the study are included in the article, uploaded as supplementary information or available upon request from the corresponding author.

PATIENT CONSENT FOR PUBLICATION

Not required.

ORCID IDs

Mari Evans https://orcid.org/0000-0001-5113-3629

Mark Corden https://orcid.org/0000-0001-6146-1634

Caroline Crehan https://orcid.org/0000-0002-3655-6954

Felicity Fitzgerald https://orcid.org/0000-0001-9594-3228

Michelle Heys https://orcid.org/0000-0002-1458-505X

For peer review only

REFERENCES

1 WHO. 2019. Newborns: reducing mortality. https://www.who.int/news-room/fact-sheets/detail/newborns-reducing-mortality [Accessed May 2020].

2 Lawn JE, Blencowe H, Oza S, et al. Every Newborn: progress, priorities, and potential beyond survival. *Lancet* 2014;384(9938):189–205.

3 WHO. 2019. Levels & trends in child mortality: report 2019. Estimates developed by the UN Inter-agency Group for Child Mortality Estimation. https://www.unicef.org/reports/levels-and-trends-child-mortality-report-2019 [Accessed May 2020].

4 WHO. 2015. WHO Guidelines Approved by the Guidelines Review Committee. https://www.who.int/publications/guidelines/guidelines_review_committee/en/ [Accessed June 2019].

5 WHO. 2017. WHO recommendations on newborn health: guidelines approved by the WHO Guidelines Review Committee. https://www.who.int/publications/i/item/WHO-MCA-17.07 [Accessed February 2021].

6 Becker JU, Theodosis C, Jacob ST, et al. Surviving sepsis in low-income and middle-income countries: new directions for care and research. *Lancet Infect Dis* 2009;9(9):577–82.

7 TDV Global. 2016. Evaluation of the Impact of WHO Publications. https://www.who.int/docs/default-source/documents/evaluation/report-evaluation-impact-who-publications.pdf?sfvrsn=577627c5_2 [Accessed February 2021].

8 Hall CS, Fottrell E, Wilkinson S, et al. Assessing the impact of mHealth interventions in low- and middle-income countries–what has been shown to work? *Glob Health Action* 2014;7:25606.

For peer review only

9 Crehan C, Kesler E, Nambiar B, et al. The NeoTree application: Developing an integrated mHealth solution to improve quality of newborn care and survival in a district hospital in Malawi. *BMJ Glob Health* 2019;4:e000860.

10 WHO. 2013. Pocket book of hospital care for children: guidelines for the management of common childhood illnesses, 2nd ed. https://apps.who.int/iris/handle/10665/81170 [Accessed June 2018].

11 COIN Course, 2017. Care of the infant and newborn in Malawi. The COIN Course Participants Manual. Available from: http://cms.medcol.mw/cms_uploaded_resources/41905_12.pdf [Accessed June 2018].

12

12 Sinha IP, Smyth RL, Williamson PR. Using the Delphi technique to determine which outcomes to measure in clinical trials: recommendations for the future based on a systematic review of existing studies. *PLoS Med* 2011;8(1):e1000393.

13 Hsu CC, Sandford BA. The Delphi Technique: Making Sense of Consensus. *Practical Assessment, Research and Evaluation* 2007;12(10). https://scholarworks.umass.edu/pare/vol12/iss1/10 [Accessed July 2018].

14 Akins RB, Tolson H, Cole BR, et al. Stability of response characteristics of a Delphi panel: application of bootstrap data expansion. *BMC Med Res Methodol* 2005;5:37.

15 WHO. 2012. Recommendations for management of common childhood conditions: evidence for technical update of pocket book recommendations: newborn conditions, dysentery, pneumonia, oxygen use and delivery, common causes of fever, severe acute malnutrition and supportive care.

For peer review only

https://apps.who.int/iris/bitstream/handle/10665/44774/9789241502825_eng.pdf?sequence=1 [Accessed June 2019].

16 European Medicines Agency. 2010. Report on the Expert Meeting on Neonatal and Paediatric Sepsis. https://www.ema.europa.eu/en/documents/report/report-expert-meeting-neonatal-paediatric-sepsis_en.pdf [Accessed July 2019].

17 Obiero CW, Seale AC, Berkley JA. Empiric treatment of neonatal sepsis in developing countries. *Pediatr Infect Dis J* 2015;34(6):659–661.

18 Babakus E, Mangold WG. Adapting the SERVQUAL scale to hospital services: an empirical investigation. *Health Serv Res* 1992;26(6):767–786.

19 Thompson CM, Puterman AS, Linley LL, et al. The value of a scoring system for hypoxic ischaemic encephalopathy in predicting neurodevelopmental outcome. *Acta Paediatr* 1997;86(7):757–761.

20 Ballard JL, Khoury JC, Wedig K, et al. New Ballard Score, expanded to include extremely premature infants. *J Pediatr* 1991;119(3):417–23.

21 Sarnat HB, Sarnat MS. Neonatal encephalopathy following fetal distress: a clinical and electroencephalographic study. *Arch Neurol* 1976;33(10):696–705.

22 Ungerer RL, Lincetto O, McGuire W, et al. Prophylactic versus selective antibiotics for term newborn infants of mothers with risk factors for neonatal infection. *Cochrane Database Syst Rev* 2004;4:CD003957.

22

23 Bhagwani DK, Sharma M, Dolker S, et al. To study the correlation of Thompson scoring in predicting early neonatal outcome in post asphyxiated term neonates. *J Clin Diagn Res* 2016;10(11):SC16-SC19.

For peer review only

24 Costa S, Rocha G, Leitao A, et al. Transient tachypnea of the newborn and congenital pneumonia: a comparative study. *J Matern Fetal Neonatal Med* 2012;25(7):992–4.

25 Tikmani SS, Muhammad AA, Shafiq Y, et al. Ambulatory treatment of fast breathing in young infants aged <60 days: A double-blind, randomized, placebo-controlled equivalence trial in low-income settlements of Karachi. *Clin Infect Dis* 2017;64(2):184–189.

26 van Haasteren A, Gille F, Fadda M, et al. Development of the mHealth App Trustworthiness checklist. *Digit Health* 2019;5:1–21

27 Kamath-Rayne BD, Thukral A, Visick MK, et al. Helping Babies Breathe, Second Edition: A Model for Strengthening Educational Programs to Increase Global Newborn Survival. *Glob Health Sci Pract* 2018;6:538–551.

28 Balikuddembe MS, Tumwesigye NN, Wakholi PK, et al. Expert perspectives on essential parameters to monitor during childbirth in low resource settings: a Delphi study in sub-Saharan Africa. *Reprod Health* 2019;16(1):119.

29 Housseine N, Punt MC, Browne JL, et al. Delphi consensus statement on intrapartum fetal monitoring in low-resource settings. *Int J Gynaecol Obstet* 2018;146(1):8–16.

30 Bakibinga P, Kamande E, Omuya M, et al. The role of a decision-support smartphone application in enhancing community health volunteers' effectiveness to improve maternal and newborn outcomes in Nairobi, Kenya: quasi-experimental research protocol. *BMJ Open* 2017;207(7):e014896.

31 Fung JST, Akech S, Kissoon N, et al. Determining predictors of sepsis at triage among children under 5 years of age in resource-limited settings: A modified Delphi process. *PLoS One* 2019;14(1):e0211274.

For peer review only

32 Pellé KG, Rambaud-Althaus C, D'Acremont V, et al. Electronic clinical decision support algorithms incorporating point-of-care diagnostic tests in low-resource settings: a target product profile. *BMJ Glob Health* 2020;5:e002067.

For peer review only

FIGURE CAPTIONS

Figure 1. Outcome of algorithm items after round one and round two of the Delphi technique.

Figure 2. Modification of the algorithms as a result of the Delphi technique.

For peer review only

Figure 1. Outcome of algorithm items after round one and round two of Delphi technique.

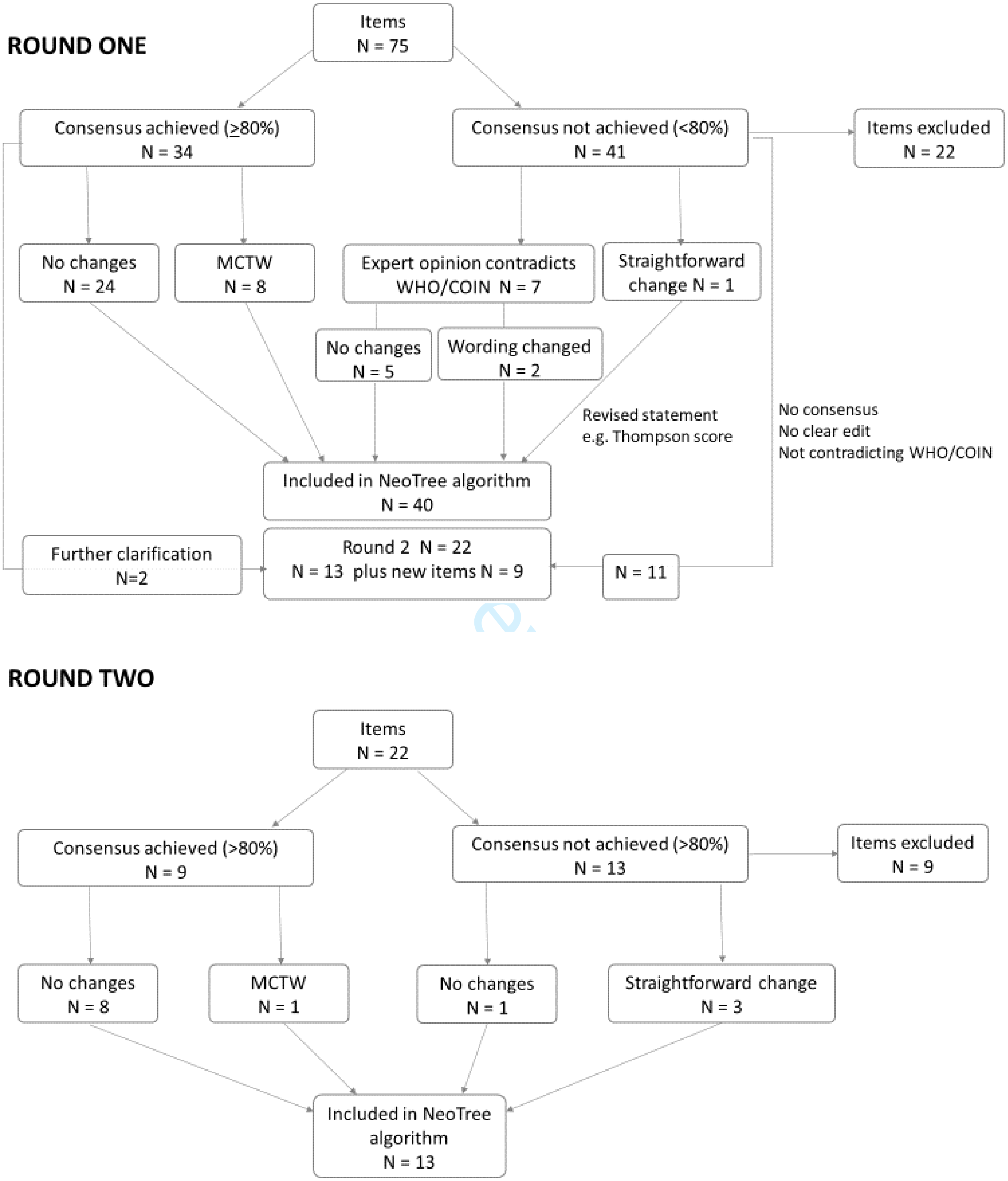

Abbreviations: COIN = Care of the Infant and Newborn in Malawi; MCTW = minor changes to wording; WHO = World Health Organisation

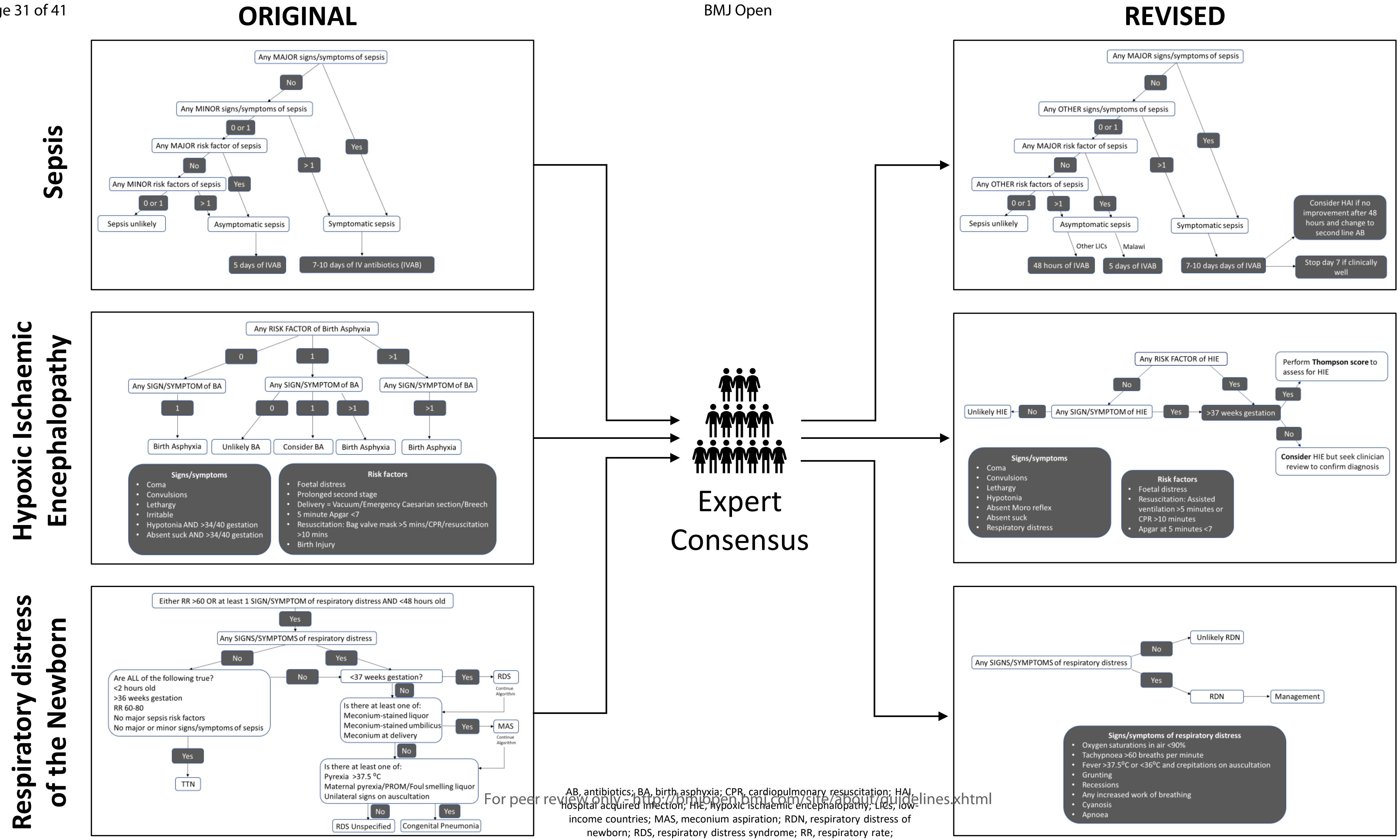

AB, antibiotics; BA, birth asphyxia; CPR, cardiopulmonary resuscitation; HAI, hospital acquired infection; HIE, hypoxic ischaemic encephalopathy; LICs, low-income countries; MAS, meconium aspiration; RDN, respiratory distress of newborn; RDS, respiratory distress syndrome; RR, respiratory rate; TTN, transient tachypnoea of newborn

For peer review only - http://bmjopen.bmj.com/site/about/guidelines.xhtml

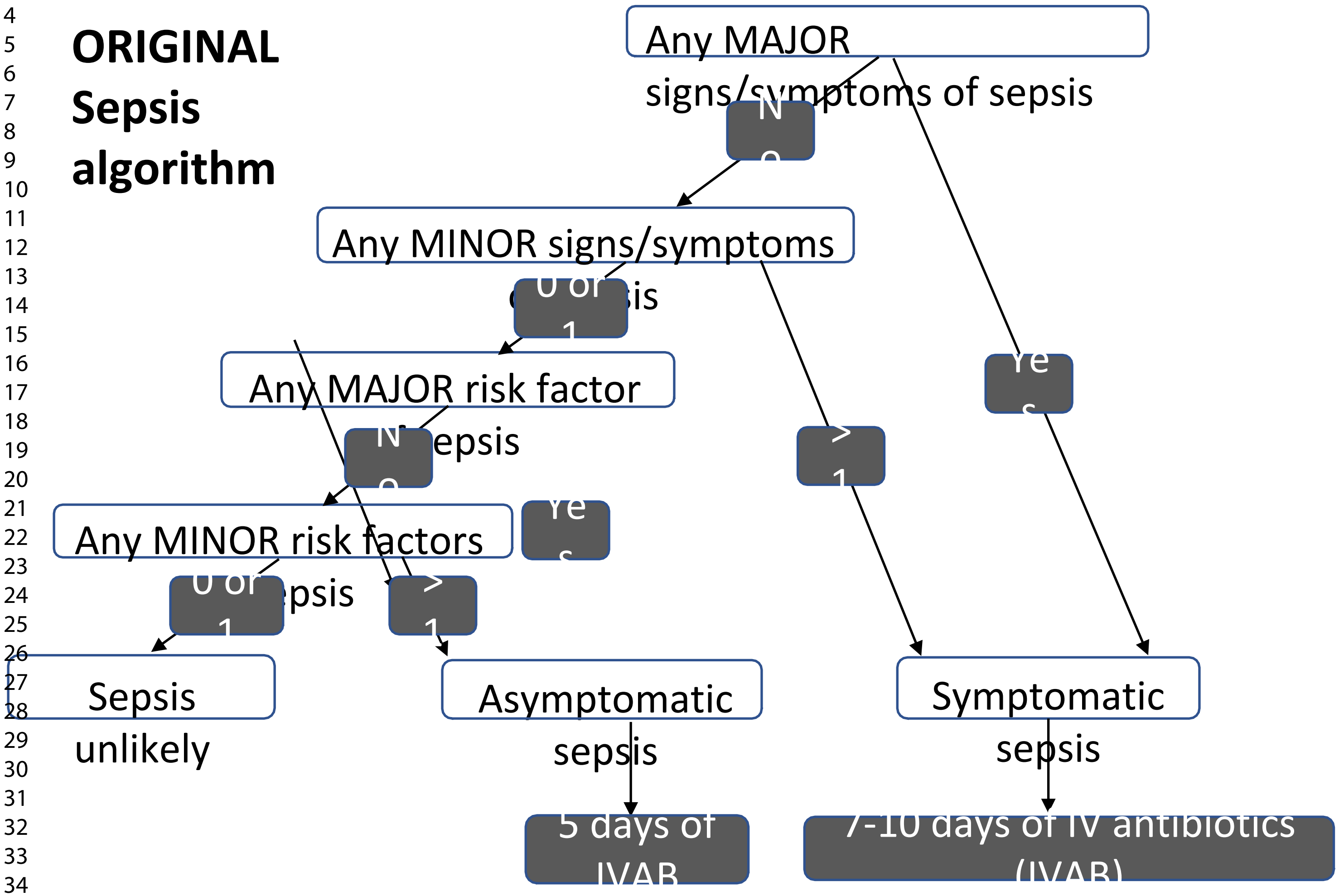
ORIGINAL Sepsis algorithm
Any MAJOR signs/symptoms of sepsis
Any MINOR signs/symptoms
0 or 1
Any MAJOR risk factor
Any MINOR risk factors
0 or 1
> 1
> 1
Sepsis unlikely
Asymptomatic sepsis
Symptomatic sepsis
5 days of IVAB
7-10 days of IV antibiotics (IVAB)

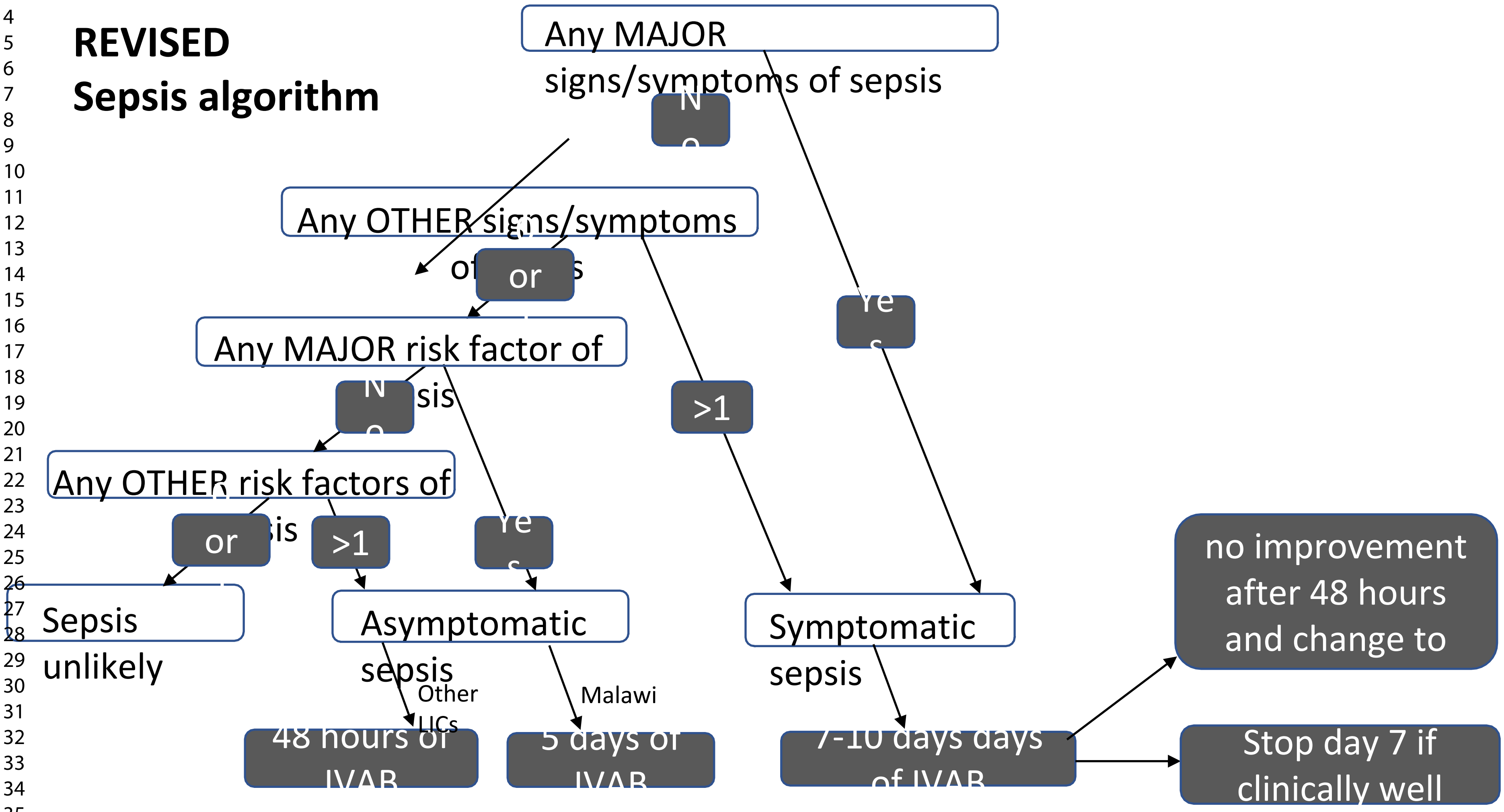
REVISED
Sepsis algorithm
Any MAJOR signs/symptoms of sepsis
Any OTHER signs/symptoms
or
Any MAJOR risk factor of
>1
Any OTHER risk factors of
or
>1
Sepsis unlikely
Asymptomatic sepsis
Symptomatic sepsis
no improvement after 48 hours and change to
Other LICs
Malawi
Stop day 7 if clinically well

Revised neonatal sepsis risk factors, signs and symptoms

Risk factors

Major
- Maternal fever >38°C in labour
- Prolonged rupture of membranes (PROM) >18 hours
- Foul smelling amniotic fluid

Other risk factors
- <32/40 weeks gestation or <1500g (spontaneous labour only)

Signs/symptoms

Major
- Neonatal temperature >37.5 °C
- Boil/abscess
- Grunting/severe respiratory distress/ mod-severe WOB
- Lethargy
- Umbilical redness extending to the periumbilical skin or umbilicus draining pus
- Deep jaundice: palms and soles of the baby deep yellow
- Tachypnoea > 60 bpm
- Convulsions/twitching or abnormal movements
- Many or severe skin pustules
- Bilious vomiting with severe abdominal distension
- Bulging fontanelle
- New onset of poor feeding
- Not moving when stimulated
- Swollen red eyelids with pus
- Central cyanosis

Other signs/symptoms
- Pallor

Revised neonatal sepsis management

Neonatal sepsis management

Investigations

- *<u>If possible</u>* perform sepsis screen: FBC, CRP, blood culture
- LP only for those who have clinical signs of meningitis (but stable) or late-onset neonatal sepsis

Antibiotic choices

- 1st line: give local recommendations
- IM/IV gentamicin and benzylpenicillin **or** ampicillin
- 2nd line: If no improvement after 48 hours change to a third-generation cephalosporin
- IV cloxacillin and gentamicin if greater risk of staphylococcal (skin infection).

Antibiotic duration (without investigations)

Asymptomatic sepsis: Treat for 48 hours and stop if well (Treat for 5 days in Malawi)

Symptomatic sepsis: Stop at day 7 if clinically well otherwise continue for 10 days

Antibiotic doses

<u>Gentamicin (IM/IV)</u>

<7 days: 3 mg/kg (LBW) and 5 mg/kg (normal BW) per dose once a day

>7 days 7.5 mg/kg once a day

<u>Ampicillin (IM/IV)</u>

<7 days: 50 mg/kg every 12 h

>7 days every 8 h

<u>Benzylpenicillin (penicillin G) (IM)</u>

<7 days: 50 000 U/kg every 12 h

>7 days every 6 h

Supportive care

- Thermoregulation: aim for 36.5-37.5 °C
- Provide respiratory support oxygen or CPAP as needed according to CPAP algorithm
- Check blood glucose and provide feeding support as needed
- Consider 10ml/kg fluid bolus if shocked as per SHOCK algorithm
- Consider 2.5mls/kg 10% dextrose if BS<2.5 mmol or 45 mg/dl – as per HYPOGLYCAEMIA algorithm

ORIGINAL
Birth Asphyxia algorithm

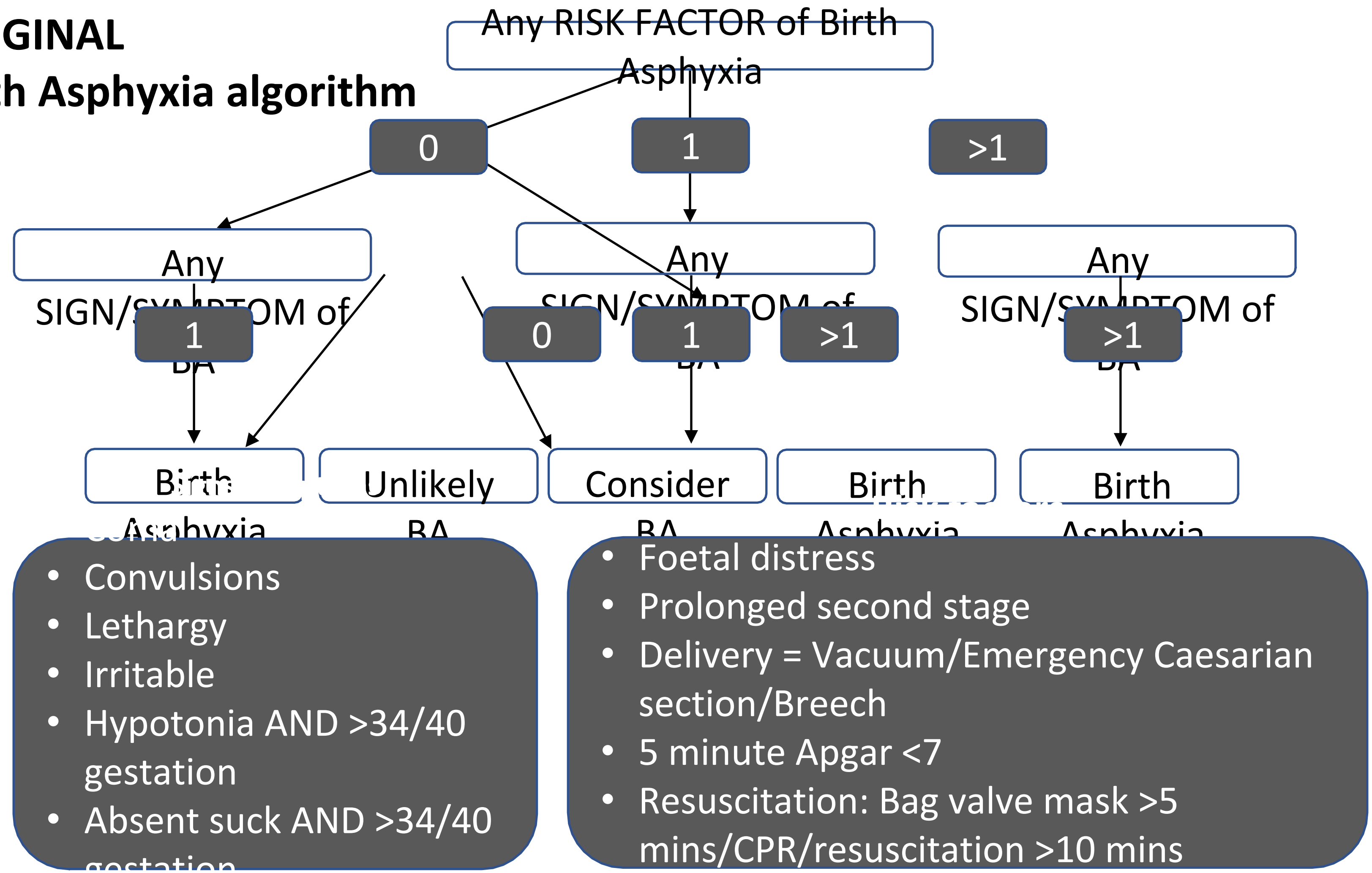

REVISED Hypoxic Ischaemic Encephalopathy algorithm

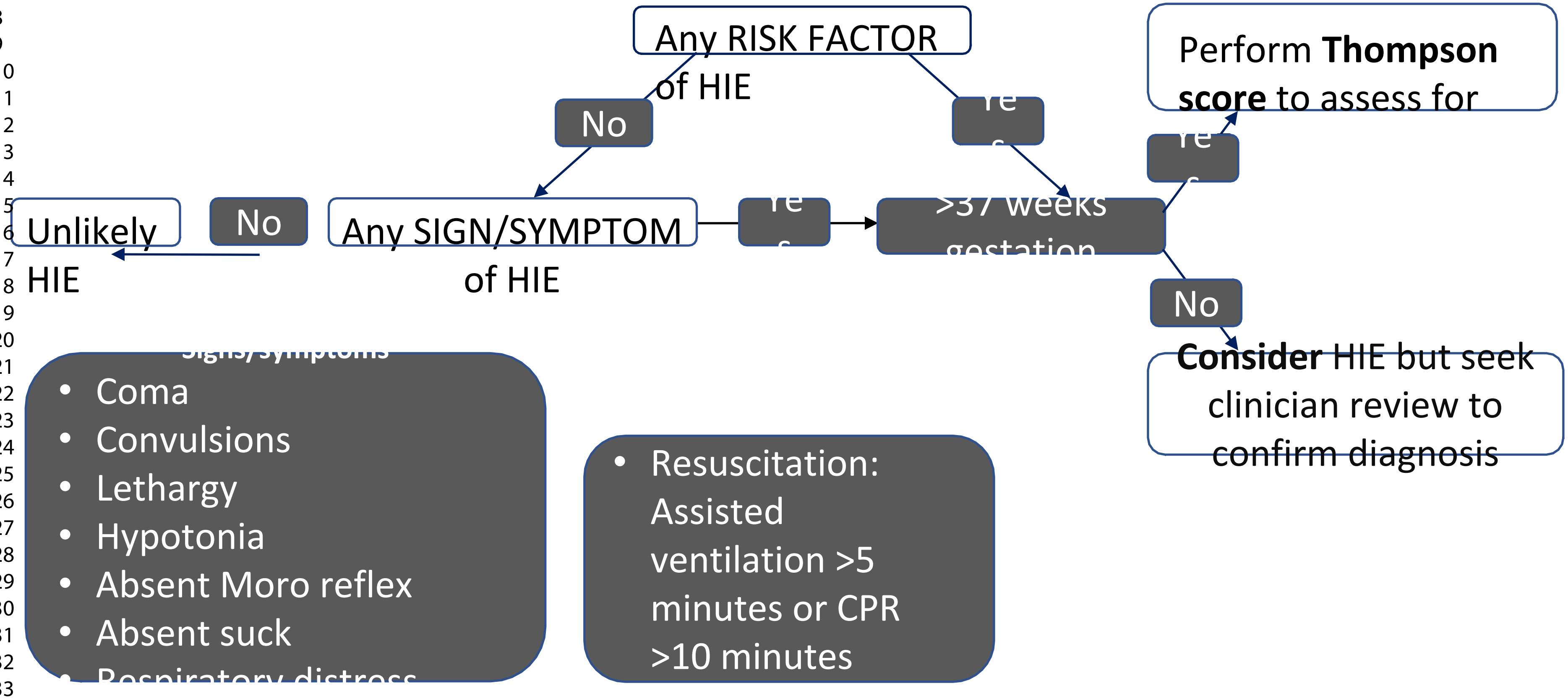

Revised Hypoxic Ischaemic Encephalopathy management

HIE management

Thermoregulation
Aim for normothermia
Avoid hyperthermia and hypothermia

Respiratory support
Give respiratory support as appropriate. Give oxygen if saturations <90% in air

Feeding support, anticonvulsants and antibiotics
Give feeding support as appropriate

1. Oral feeds (breast feed or bottle if breastfeeding is not possible)
2. Give NG feeds if BS <2.5mmol/L or <45mg/dL and not tolerating oral feeds
3. Give IV fluids if BS <2.5mmol/L or <45mg/dL and not tolerating NG feed

If evidence of seizure activity give Phenobarbitone as per convulsions algorithm.
Reassess suck, tone, coma, seizures every 8 hours for 72 hours in hospital
Treat for sepsis with antibiotics

ORIGINAL RDN algorithm

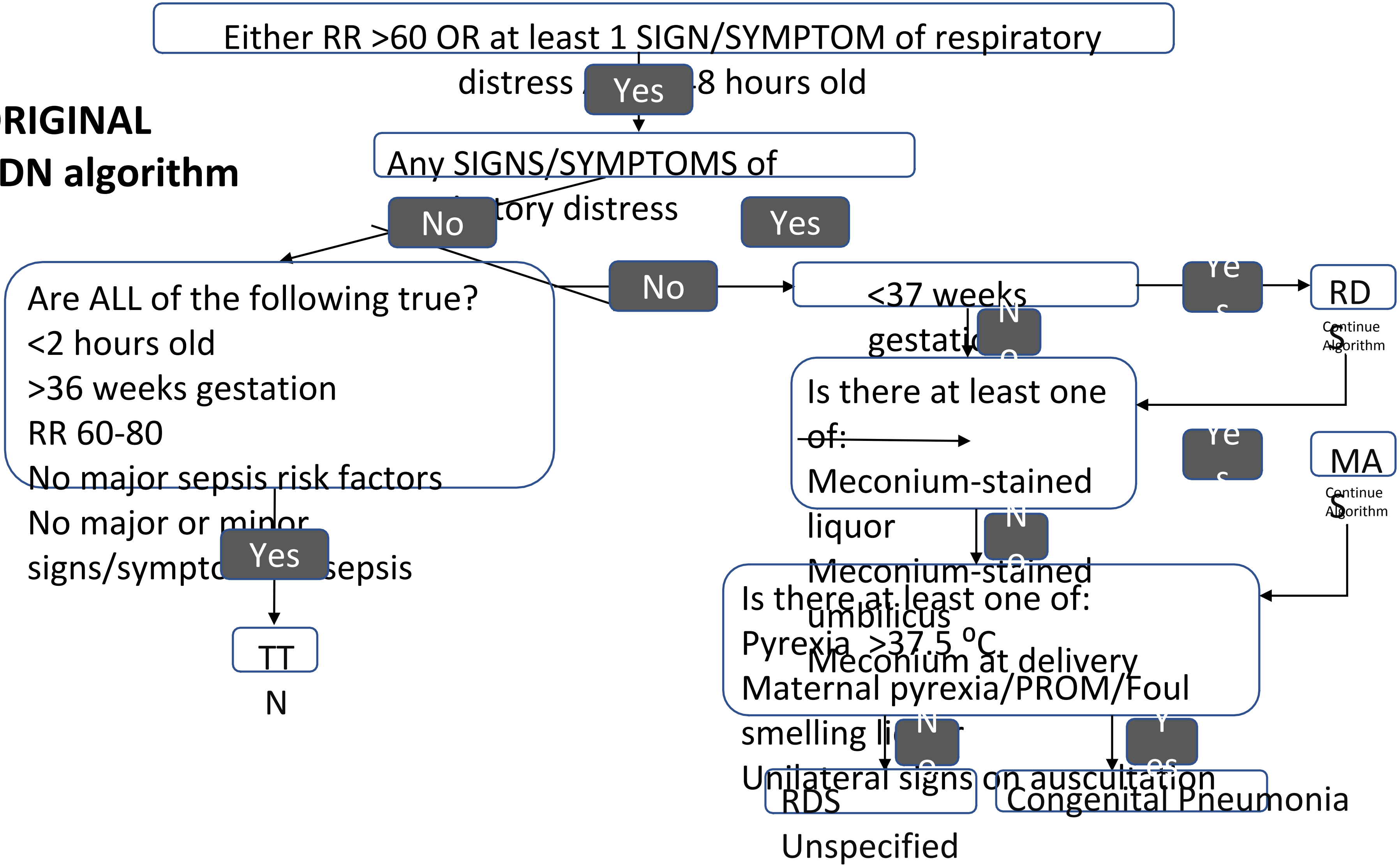

REVISED RDN management

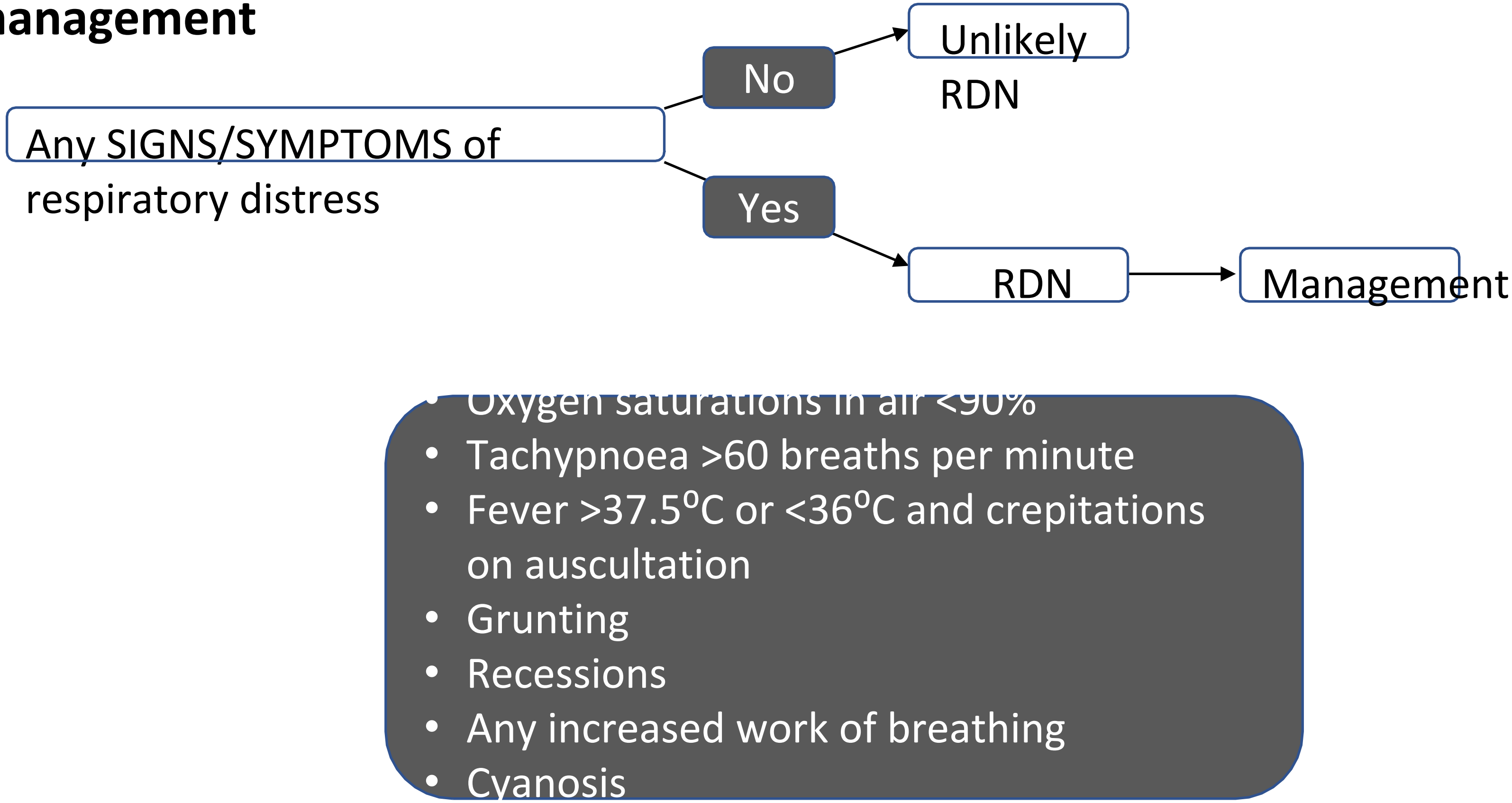

Revised Respiratory Distress of the Newborn management

Respiratory distress of the newborn management

Investigations
Perform a chest x-ray if it will change management or if the infant is not improving as expected or deteriorating.

Airway and respiratory support
Position airway
Give oxygen if oxygen saturations < 90% in air
If > 1kg consider CPAP according to CPAP algorithm

Feeding support
If breathing 60-80 bpm use cup/NGT
If needing CPAP use OGT
If breathing > 80bpm consider IV fluids

IV Access for Antibiotics
Give antibiotics including for suspected TTN (unless have chest x-ray and can safely exclude.)

Teaching points

Meconium aspiration
- Consider if meconium stained amniotic fluid
- Just because there was meconium at delivery doesn't mean it was aspirated.
- Most aspiration occurs in utero before the baby is born, so more likely if there was foetal distress in labour or has signs of HIE
- Floppy babies with severe HIE and meconium aspiration are unlikely to benefit from CPAP.

Transient tachypnoea of the newborn (TTN)
- These babies are not unwell
- RR is usually between 60-80 bpm and they don't usually need oxygen
- This should resolve in the first few hours of life.
- TTN is more likely following elective C-section or a rapid vaginal delivery.
- Give antibiotics if unable to exclude other causes i.e no chest xray

Respiratory distress syndrome
- Consider if < 37 weeks or diabetic mother
- Benefit from CPAP see Algorithm

Congenital Pneumonia
- Consider if temperature > 37.5 ^{o} c or < 36 ^{o} c and crepitations on auscultation
- Risk factors for sepsis i.e maternal fever/PROM/foul smelling amniotic fluid

Hypothermia Algorithm and management

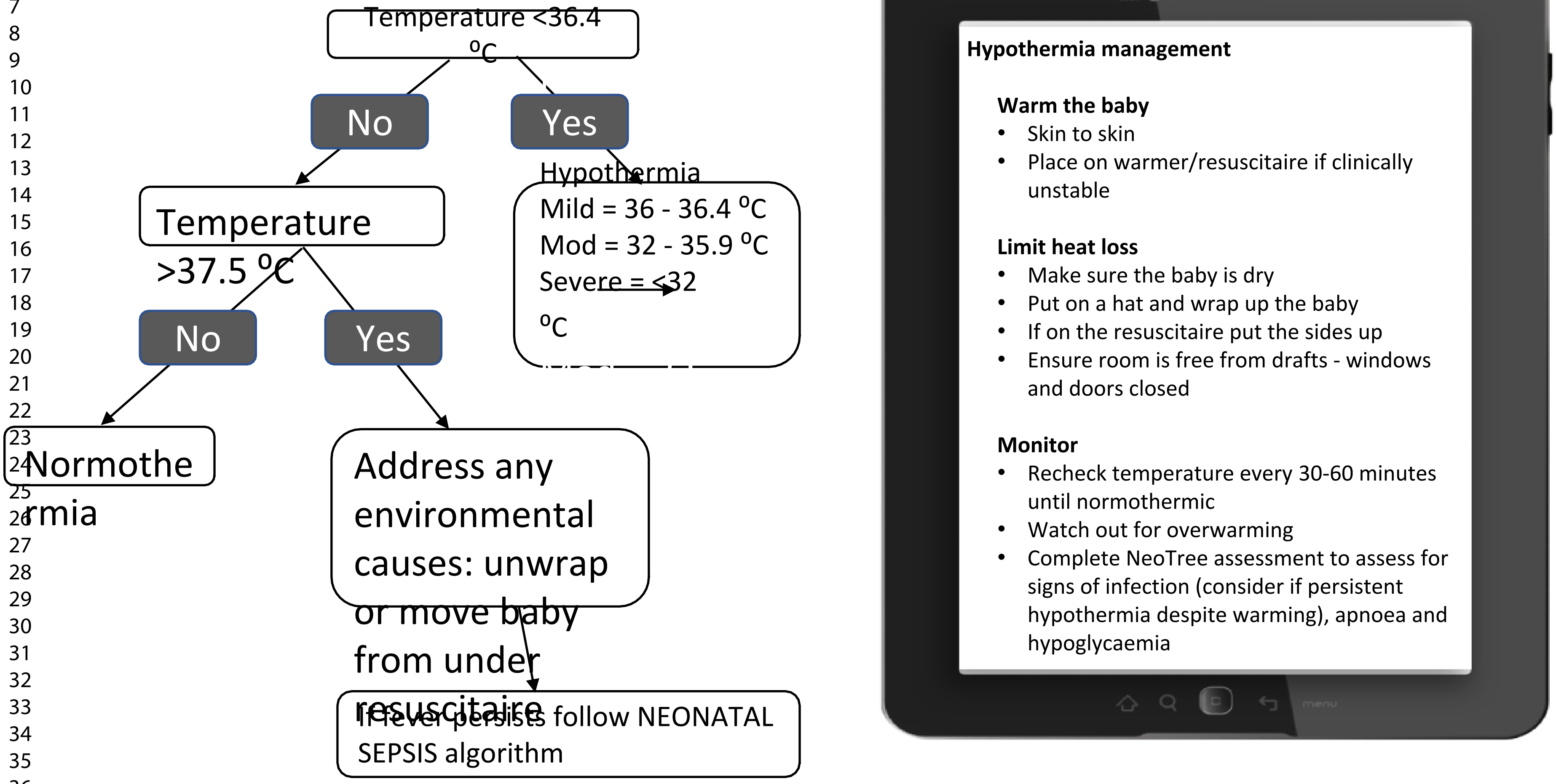

BMJ Open

Refining clinical algorithms for a neonatal digital platform for low-income countries: A modified Delphi technique

Journal:	*BMJ Open*
Manuscript ID	bmjopen-2020-042124.R2
Article Type:	Original research
Date Submitted by the Author:	26-Mar-2021
Complete List of Authors:	Evans, Mari; Great Ormond Street Hospital for Children, UCL Institute of Child Health<br>Corden, Mark; Children's Hospital of Los Angeles, Division of Hospital Medicine, Department of Pediatrics; University of Southern California Keck School of Medicine, Pediatrics<br>Crehan, Caroline; Great Ormond Street Hospital for Children, UCL Institute of Child Health<br>Fitzgerald, Felicity; Great Ormond Street Hospital for Children, UCL Institute of Child Health<br>Heys, Michelle; Great Ormond Street Hospital for Children, UCL Institute of Child Health
**Primary Subject Heading**:	Global health
Secondary Subject Heading:	Paediatrics, Health informatics
Keywords:	PUBLIC HEALTH, Information technology < BIOTECHNOLOGY & BIOINFORMATICS, Neonatal intensive & critical care < INTENSIVE & CRITICAL CARE, NEONATOLOGY

SCHOLARONE™
Manuscripts

I, the Submitting Author has the right to grant and does grant on behalf of all authors of the Work (as defined in the below author licence), an exclusive licence and/or a non-exclusive licence for contributions from authors who are: i) UK Crown employees; ii) where BMJ has agreed a CC-BY licence shall apply, and/or iii) in accordance with the terms applicable for US Federal Government officers or employees acting as part of their official duties; on a worldwide, perpetual, irrevocable, royalty-free basis to BMJ Publishing Group Ltd ("BMJ") its licensees and where the relevant Journal is co-owned by BMJ to the co-owners of the Journal, to publish the Work in this journal and any other BMJ products and to exploit all rights, as set out in our licence.

The Submitting Author accepts and understands that any supply made under these terms is made by BMJ to the Submitting Author unless you are acting as an employee on behalf of your employer or a postgraduate student of an affiliated institution which is paying any applicable article publishing charge ("APC") for Open Access articles. Where the Submitting Author wishes to make the Work available on an Open Access basis (and intends to pay the relevant APC), the terms of reuse of such Open Access shall be governed by a Creative Commons licence – details of these licences and which Creative Commons licence will apply to this Work are set out in our licence referred to above.

Other than as permitted in any relevant BMJ Author's Self Archiving Policies, I confirm this Work has not been accepted for publication elsewhere, is not being considered for publication elsewhere and does not duplicate material already published. I confirm all authors consent to publication of this Work and authorise the granting of this licence.

For peer review only

Refining clinical algorithms for a neonatal digital platform for low-income countries: a modified Delphi technique

Mari Evans,[1*] Mark H. Corden,[2,3*] Caroline Crehan,[1] Felicity Fitzgerald, [1] Michelle Heys[1,4]

[1]Great Ormond Street Institute of Child Health, University College London, London, UK
[2]Division of Hospital Medicine, Department of Pediatrics, Children's Hospital Los Angeles, Los Angeles, CA, USA
[3]Department of Pediatrics, Keck School of Medicine, University of Southern California, Los Angeles, CA, USA
[4]Specialist Children's and Young People's Services, East London NHS Foundation Trust, London, UK

[*]denotes co-first authors

Corresponding Author:
Dr Michelle Heys
UCL Great Ormond Street Institute of Child Health
30 Guilford Street
London, WC1N 1EH
United Kingdom
m.heys@ucl.ac.uk

Word count: 4020

For peer review only

ABSTRACT

Objectives

To determine whether a panel of neonatal experts could address evidence gaps in local and international neonatal guidelines by reaching a consensus on four clinical decision algorithms for a neonatal digital platform (NeoTree).

Design

Two-round, modified Delphi technique.

Setting and participants

Participants were neonatal experts from high-income and low-income countries (LICs).

Methods

This was a consensus-generating study. In round one, experts rated items for four clinical algorithms (neonatal sepsis, hypoxic ischaemic encephalopathy, respiratory distress of the newborn, hypothermia) and justified their responses. Items meeting consensus for inclusion (≥80% agreement) were incorporated into the algorithms. Items not meeting consensus were either excluded, included following revisions or included if they contained core elements of evidence-based guidelines. In round two, experts rated items from round one that did not reach consensus.

Results

Fourteen experts participated in round one, ten in round two. Nine were from high-income countries, five from LICs. Experts included physicians and nurse practitioners with an average neonatal experience of 20 years, 12 in LICs. After two rounds, a consensus was reached on 43 of 84 items (52%). Per experts' recommendations, items in line with local and WHO guidelines yet

For peer review only

not meeting consensus were still included to encourage consistency for frontline healthcare workers. As a result, the final algorithms included 53 items (62%).

Conclusion

Four algorithms in a neonatal digital platform were reviewed and refined by consensus expert opinion. Revisions to NeoTree will be made in response to these findings. Next steps include clinical validation of the algorithms.

KEYWORDS

Information technology, neonatal intensive & critical care, neonatology, public health

For peer review only

STRENGTHS AND LIMITATIONS OF THIS STUDY

- In this study, a large number of algorithm items were reviewed and evaluated, and half met consensus for inclusion in the management pathways.
- The review was conducted with experts from a broad range of countries and neonatal experience who simultaneously refined the algorithms and highlighted gaps in current evidence, emphasising the need for future research to support international neonatal guidelines.
- Our study method meant that experts were not able to meet in person, which might have promoted dialogue that would have allowed greater clarity in their collective opinion.
- The representation of neonatal experts from LICs was not as robust as from high-income countries, which may have led to an uneven evaluation of the algorithms.

For peer review only

INTRODUCTION

Globally, 2.5 million newborns die each year in the first 28 days of life.[1] Most of these deaths (98.5%) occur in low-income countries (LICs), and 40% occur on the first day of life.[2] The neonatal mortality rate has halved since 1990,[3] but modelling of global newborn mortality data suggests that a further two-thirds of current deaths could be prevented if evidence-based solutions were implemented.[2] One of the World Health Organisation (WHO) Sustainable Development Goals (SDG3) is to end preventable deaths of newborns in all countries and to reduce the neonatal mortality rate from the current rate of 18 per 1000 live births to less than 12 per 1000 by 2030.[4] Targeting newborn care in LICs is thus an urgent priority, especially the three most common causes of mortality – infections (36%), prematurity (28%), and intrapartum complications (23%).[2]

The WHO neonatal guidelines are internationally recognised as the leading and most respected source of guidance.[5] However, one of their limitations is that they are primarily based on data from high-income countries, as there is often a lack of evidence in LICs due to limited diagnostic aids, data and research.[6] The WHO aims to address the challenge of developing setting-appropriate neonatal guidelines by improving stakeholder involvement (design guidelines for specific audiences), clarity of presentation (often guidelines are too long and technical), and attention to dissemination.[7] Mobile health (m-health) technology and digital platforms are potential approaches to implementing these measures and improving the quality of newborn care.[8]

An international team of researchers, clinicians and software developers in the UK, USA, Malawi, Bangladesh and Zimbabwe co-designed and co-developed with Malawian and Zimbabwean HCWs a neonatal digital platform (NeoTree) for facility-based newborn care in

For peer review only

LICs. It combines immediate digital data capture (which is shared with HCWs via local dashboards), evidence-based algorithmic clinical decision and management support, newborn education and data linkage to national data systems on one platform.[9] The algorithms in the Malawian version of the NeoTree support decisions according to established international[10] and Malawian neonatal guidelines.[11] In situations where guidelines were not applicable, the NeoTree clinical team used clinical judgment to complete the algorithm development. In the absence of extensive trial or epidemiological data in LICs, alternative techniques to consolidate best available low-quality evidence can be used, such as expert opinion. This study aims to use the modified Delphi technique to determine whether a panel of experts in newborn care can reach a consensus opinion about key clinical decision algorithms used in a digital platform to assist HCWs caring for facility-based unwell newborns in LICs.

METHODS

Study design

This study used a two-step modified Delphi technique.[12] The Delphi technique was chosen because it is an effective method of gathering expert knowledge from geographically diverse leaders in the field to address complex clinical problems that lack evidence.

Recruitment

Twenty-two neonatal experts were invited to participate in the study. This number represented an adequate sample size[13, 14] and permitted a manageable amount of data collection. Participants were recruited if they were a physician or neonatal nurse practitioner with more than ten years neonatal experience (at least three in LICs), neonatal postgraduate training, fluency in English, internet access and willingness to participate. Neonatal experts known to the researchers for their clinical expertise, research and contributions to guideline development in LICs were identified in

For peer review only

equal numbers from both LICs and HICs. No financial incentive was offered, but reimbursement for costs of Skype calls was provided for some experts in LICs.

Algorithms and item generation

The four clinical decision algorithms selected for review were neonatal sepsis, hypoxic ischaemic encephalopathy (HIE), respiratory distress of the newborn and hypothermia. These conditions represent the leading preventable causes of neonatal mortality and are the most difficult to diagnose and manage appropriately in LICs with some of the weakest WHO GRADE recommendations and quality of evidence.[15] For example, the European definition of neonatal sepsis is two or more clinical symptoms and two or more laboratory signs in the presence of, or as a result of, suspected or proven infection.[16] This definition is not possible in LICs where laboratory investigations are not routinely available.[17]

Items were identified by comparing the algorithms side-by-side with the international (WHO) and local neonatal guidelines (Care of the Infant and Newborn in Malawi - COIN) from which they had been derived. This comparison generated a comprehensive list of items where discrepancies in diagnostic parameters and treatment recommendations required expert opinion. Once finalised, the clinical algorithms and list of items (henceforth referred to as questionnaire) were piloted with two paediatricians with neonatal experience in LICs. Ambiguous items were amended accordingly.

Delphi technique

The questionnaire was circulated by email to the experts with specific instructions at least two weeks before they were interviewed. Each algorithm was verbally and diagrammatically explained with their references specified (i.e., WHO, COIN or NeoTree research team) to aid in decision-making during the interview (online supplemental file 1). Round one interviews were

For peer review only

conducted in June and July 2018. Experts were sent up to two reminder emails to schedule their phone or Skype interview. Interviews were conducted privately from a home office. Standardised questions were used to review each item from the questionnaire. Experts were asked to rate their level of agreement for including an item in an algorithm using a five-point Likert scale. A five-point scale was chosen because evidence suggests that a five-point scale appears to be less confusing than a seven-point scale and to increase expert response rate.[18] Each rating was followed by open-ended questions to obtain the experts' rationale for their response and any amendment or additional items they would propose. All interview data were transcribed using both audio-recordings and notes made during the interview by the facilitator. All responses were anonymised (with participant numbers) and reviewed together with the quantitative results.

The upper limit of agreement amongst experts has been recommended to be set at 80% (4 or higher on the Likert scale) for Delphi studies.[13] Due to our sample size, this upper limit was used to apply greater rigor to item inclusion. Items that met consensus (≥80% agreement) were included or were modified with minor changes to wording based on expert advice. Items that did not meet consensus (<80%) were removed or modified according to the feedback from the expert panel and submitted for the second round. Items that did not meet consensus were still included if they were part of WHO and COIN guidelines so that frontline HCWs continued to follow the current standard of care. A second questionnaire was designed with modified items and expert additions from the first round (e.g., inclusion of the Thompson encephalopathy score[19]).

In round two (June and July 2019), this second questionnaire was distributed electronically to the 14 experts from round one (online supplemental file 1). A results summary from round one was sent to the experts, and the full set of anonymised results were made available at their request. Two email reminders were sent to non-responders. Experts again rated items on a Likert scale

For peer review only

and explained their ratings. Responses were analysed as described in round one, and items meeting ≥80% consensus were kept for the final NeoTree algorithms.

Patient and public involvement

While key stakeholders were involved in co-developing the NeoTree digital platform, there was no patient or public involvement in this Delphi study.

Consent procedures

The goals and processes of the project were explained to the experts in their email invitation, and consent was obtained by email agreement. Experts were verbally informed at the beginning of the first round that their responses would be kept anonymous.

RESULTS

Twenty-two neonatal experts were invited to participate. Sixteen responded; one declined due to lack of financial incentive, and one declined due to conflict of interest. All respondents had work experience in Africa; one respondent had over 20 years of clinical experience in Malawi and contributed to the development of the COIN guidelines. Demographics of the expert panel are listed in Table 1.

Table 1. Characteristics of the Delphi panel from round one.

	**Characteristics**	**N = 14 (%)**
Location	Experts from HICs	9 (64)
	Experts from LICs	5 (36)
Level of expertise	Neonatologist	6 (43)
	Paediatrician	6 (43)
	ANNP	2 (14)
Years of neonatal experience following graduate degree (mean ± SD)	Overall	20 (± 12)
	In LICs	12 (± 7)
Work experience in LICs	Africa	14 (100)
	Asia	7 (50)
	Central America	4 (28)
Country of medical degree	United Kingdom	7

For peer review only

United States	2
South Africa	2
Rwanda	1
Sudan	1
Zimbabwe	1

ANNP, advanced neonatal nurse practitioner; HICs, high-income countries; LICs, low-income countries

Round One

Fourteen experts (63% response rate) completed round one. Interviews averaged 73 minutes (40-110 minutes). Thirty-four items (45%) reached consensus (Figure 1). These items were either: (1) included, unmodified (32%); (2) included, modified (11%); or (3) changed for clarification in the second round (2%). Items that did not reach consensus (55%) were either: (1) excluded from the revised algorithm (30%); (2) included because they were part of WHO/COIN guidelines or the Thompson score (11%); or (3) changed and submitted for a second round (14%) (Table 2). The expert panel consistently stated that algorithm items must comply with WHO danger signs and COIN guidelines for neonatal sepsis, irrespective of whether the panel agreed with them. For example, experts thought that ‘bulging fontanelle’ was “*subjective; there are non-infectious causes … many babies' fontanelles bulge when they just cry.”* Another item that did not meet consensus was ‘poor feeding,’ which experts found vague for multiple reasons, including*: “it is too subjective; it depends on how long for … many newborns do not feed well on the first day of life.*” However, experts agreed that poor feeding was a sign of possible sepsis if it was *“a new onset of poor feeding when the infant had previously been feeding well.”* This item was changed to ‘new onset of poor feeding’ for the final algorithm.

Two items that were included because they are part of the COIN guidelines highlighted inconsistencies with WHO guidelines. For example, COIN uses a temperature of more than 37.5°C as a fever for a newborn, whilst WHO and most experts use more than 38°C. Therefore,

37.5°C was included for the Malawian digital platform, but 38°C will be used for other countries. Other items where a difference between the recommendations and the guidelines occurred were antibiotic choice and duration for neonatal sepsis.

Modifications usually involved adopting the language utilized by WHO or COIN, but there were items that experts felt needed clarifying. For example, experts felt that *"twitching or abnormal movements"* needed to be added to the WHO term 'convulsions' because seizures in a neonate can be very subtle. Certain items that could not be revised easily were submitted for the second round according to feedback from the expert panel. For example, experts disagreed that 'very/extremely premature (<32 weeks gestation)' was a major risk factor for sepsis if *"the baby was delivered as a clean cold caesarean section for maternal reasons and the mother was not in labour."* Eighty percent of experts highlighted that the algorithm should include weight to guide gestation because "*gestation is often unknown*" and "*you are relying on [the] Ballard score[20] which has plus or minus two weeks accuracy."* Similar opinions regarding method of delivery and the importance of birth weight were expressed for 'slightly premature (32-36 weeks gestation).' Both gestational age brackets were submitted into the second round as risk factors for sepsis after modifying the items to include WHO weight parameters to guide gestation.

Other items that did not gain consensus and were submitted for the second round included items that experts felt needed further clarification. 'Born before arrival' as a minor risk factor for sepsis was clarified to the experts that this meant the baby was born en-route to the hospital (either in a vehicle or on the roadside, both being considered dirty environments in Malawi). A 'neonate admitted with or history of a fever' as a minor risk factor for sepsis was changed to 'mother reports a non-measured fever' in the second round. Lastly, because experts considered the term

For peer review only

'birth injury' unclear, we asked them in round two to define what they considered a 'significant birth injury.'

Table 2. Round one heat chart to show which items met consensus and their outcomes.

Subject	Items	Agree	Outcome
**Sepsis Diagnosis** Major RF	Maternal fever >38°C in labour	91%	Include
	PROM > 18 hrs	74%	Include (WHO RF)
	Offensive smelling liquor	74%	Include (WHO RF); MCTW
	Very / extremely premature (<32/40 weeks)	74%	Second round
	Prolonged second stage (>3 hrs)	53%	Exclude
Minor RF	Prematurity (32-37 weeks gestation)	81%	Second round
	Born before arrival	70%	Second round
Major signs/ symptoms	Boil/abscess	93%	Include
	Grunting or severe respiratory distress or moderate-severe work of breathing	97%	Include
	Lethargy	93%	Include
	Red skin all around umbilicus	81%	Include, MCTW
	Jaundice <24 hrs old	83%	Include, MCTW
	Tachypnoea >60 bpm (>2 hrs old)	83%	Include, MCTW
	Convulsions	89%	Include, MCTW
	Pustules all over body	80%	Include, MCTW
	Bulging fontanelle	77%	Include (WHO danger sign)
	Temperature >37.5°C	73%	Include for Malawi version
	Admitted with or history of fever	68%	Second round
	History of apnoea	67%	Exclude
	Bilious vomiting	61%	Second round
Minor signs/ symptoms	Tachypnoea 60-80 bpm & <2 hrs old	85%	Include
	Pallor	81%	Include
	Weak or absent suck (AND >34/40 weeks)	73%	Exclude
	Poor feeding	73%	Include (WHO danger sign), MCTW
	Irritability	70%	Second round
	Distended abdomen	67%	Second round
	Heart rate >160 that cannot be explained by fever/crying	64%	Exclude
	Mild work of breathing	55%	Exclude
Additional RF?	Cut-off at 72 hrs for early vs late neonatal sepsis?	100%	Include
	Definition of maternal fever >38°C?	93%	Include
	Should PROM be >18 hrs or >24 hrs in LICs?	93%	Include >18 hrs
	Hypothermia < 35.5 °C	83%	Second round
	Fever in a newborn should be classified as >37.5°C in this setting?	83%	Include
	Please comment on our weighting system of Major = 100% / Minor = 50%	77%	Exclude
	Cut-off at >34/40 weeks for absent suck as a sign of sepsis?	60%	Exclude
	Reduced movement of limbs	43%	Exclude
	Joint swelling	42%	Exclude
	Criteria for 'consider meningitis'	42%	Second round
Management	Do you agree with the antibiotic doses?	93%	Include

	Do you agree with the specified sepsis investigations if possible?	83%	Include
	Antibiotic duration for symptomatic sepsis = 7-10 days?	83%	Include (change to stop at day 7 if clinically well)
	Do you agree with the antibiotic choices if no local recommendation?	80%	Include (add WHO choices)
	Antibiotic duration for asymptomatic sepsis = 5 days?	66%	Include for Malawi, exclude for international
**Birth Asphyxia Diagnosis**	Resuscitation: BVM >5 mins / CPR >10 mins	94%	Include
	Foetal distress	86%	Include, MCTW
	Apgar at 5 mins <7	78%	Include as per COIN
	Vaginal breech	76%	Exclude
	Prolonged second stage >3 hrs duration	73%	Exclude
	Vacuum delivery	69%	Exclude
	Emergency caesarean section	61%	Exclude
	Birth injury	44%	Second round
Signs/ symptoms	Convulsions	93%	Include
	Coma	89%	Include
	Lethargy	84%	Include
	Hypotonia and gestation >34/40 weeks	80%	Include
	Irritable	77%	Exclude
	Absent suck and gestation >34/40 weeks	64%	Second round
	Should birth asphyxia be classified as mild, moderate or severe?	54%	Exclude
Additional RF?	Exclude the Moro reflex in LICs due to the difficulties of training HCW in checking safely?	80%	Include as part of Thompson score
	Poor feeding	49%	Exclude
	Respiratory distress	43%	Include as part of Thompson score
	Weight >4kg	43%	Exclude
Management	Give intravenous fluids if not tolerating oral or nasogastric feeds?	97%	Include
	No passive cooling for infants in LICs?	94%	Include
**RDN Diagnosis**	Do you agree with tachypnoea of >60 bpm for the other categories of RDN?	100%	Include
	Tachypnoea of 60-80 bpm <2 hrs old without signs or symptoms of sepsis should be treated as TTN and no antibiotics given?	76%	Exclude
	History of fast/laboured/noisy breathing is relevant as a sign or symptomatic of RDN when not present on admission?	44%	Exclude
RDN diagnostic criteria	Meconium aspiration syndrome	82%	Include
	Transient tachypnoea of the newborn	66%	Exclude
	Respiratory distress syndrome	63%	Second round
	Congenital pneumonia	63%	Second round
Management	Cut-off of 90% O_2 saturations before giving oxygen?	91%	Include
	Give antibiotics in all cases except TTN?	77%	Exclude
	Time cut-off of 2 hours for TTN? Would you have a higher or lower threshold?	54%	Exclude
**Hypothermia**	Diagnostic criteria for hypothermia?	100%	Include

BPM, breaths per minute; BVM, bag valve mask; CPR, cardiopulmonary resuscitation; HCW, heath care worker; LICs, low-income countries; MCTW, minor changes to wording; PROM, prolonged rupture of membranes; RDN,

respiratory distress of the newborn; RF, risk factor; TTN, transient tachypnoea of the newborn; WHO, World Health Organisation

Key findings by algorithm

The first important finding was that the 'major' or 'minor' algorithmic weighting system (where one major risk factor for sepsis is equivalent to two minors) used to diagnose neonatal sepsis was near consensus (77%) but did not meet the 80% threshold. Experts called for further evidence before adopting this system: *"It is a difficult thing to do…you need to work out how specific and sensitive the app is by looking at blood cultures."* Two experts suggested using a sepsis risk score calculator, and another two experts highlighted that WHO only uses danger signs. This weighting system was subsequently removed from the algorithm.

The second significant algorithmic finding was on HIE. An academic expert in neonatal encephalopathy discouraged the use of the term 'birth asphyxia,' a term used by Malawian HCWs and therefore incorporated into the original algorithm.

> *You really must not call it birth asphyxia because birth asphyxia means failing to breathe at birth and what you are talking about is encephalopathy.*

Additional feedback on the algorithm focused on the combination of risk factors or clinical signs and symptoms to consider or diagnose HIE. Experts cited a lack of evidence for using risk factors to diagnose birth asphyxia and that the digital platform should only be using clinical signs and symptoms.

> *Birth asphyxia is not about risk factors. If you have encephalopathy, it is a clinical diagnosis, and it is irrelevant what your risk factors are.*

Experts recommended utilizing a validated encephalopathy score,[19, 21] which was incorporated into the HIE algorithm. The risk factors that met consensus may be used as prompts

For peer review only

to perform the Thompson score, which uses clinical signs and symptoms exclusively to diagnose HIE.

Third, for the respiratory algorithm, experts highlighted that "*It is hard to make an accurate diagnosis of a respiratory condition without investigations*." Therefore, the algorithm should focus instead on the management of respiratory distress. All respiratory conditions (respiratory distress syndrome, meconium aspiration, congenital pneumonia and transient tachypnoea of the newborn (TTN)) now fall under the umbrella diagnosis of respiratory distress of the newborn within the algorithm. For teaching purposes, the four respiratory conditions will be included as 'diagnoses to consider' in the management.

Finally, for the hypothermia algorithm, experts commented that first-line treatment for all newborns be skin-to-skin care including those who were severely hypothermic (<32°C) unless they showed any signs or symptoms of being unstable. Additionally, experts did not think it was realistic to review a newborn every 15-30 minutes when hypothermic. No major revisions were made to the hypothermia algorithm.

Round Two

Ten (71%) experts completed round two, seven electronically and three by telephone interview. Four experts dropped out (three from HICs, one from LIC); three did not respond to email reminders and one expert was unable to meet the completion deadline. Nine items (41%) reached consensus (Figure 1). These items were either (1) included, unmodified (36%) or (2) included, modified in the revised algorithm (5%). Items that did not reach consensus (59%) were either (1) excluded (41%) or (2) included, modified according to WHO guidelines or expert suggestion (18%) (Table 3).

For peer review only

In round one, experts indicated that hypothermia was a major sign of sepsis and should be included in the sepsis algorithm if persistent. In round two, we clarified that the digital platform is to be used at the time of admission onto the neonatal unit, at which point the HCW will only have one temperature reading. Experts in round two disagreed that a single temperature reading of <35.5^{0}C was a sign of sepsis and felt that it would more likely be due to environmental hypothermia, a common problem in LICs. Additionally, in round two, it was established that experts were much more concerned with extremely premature and/or <1500g neonates (88% consensus) compared to slightly premature and/or 1500-2500g neonates (62% consensus) being at risk for neonatal sepsis. Central cyanosis was an addition to the second round as an expert suggestion to include all WHO danger signs; despite missing consensus (with 78% agreement), it was ultimately included in the final sepsis algorithm to comply with WHO guidelines for danger signs.

All of the respiratory items marginally missed consensus. All three items were still included in the education sections of the digital platform with revisions in line with WHO diagnostic criteria. Modifications to the algorithms can be found in Figure 2 and online supplemental file 2.

Table 3. Round two heat chart to show which items met consensus and their outcomes.

Subject	Items	Agree	Outcome
**Sepsis Diagnosis** 'Other' RF	<32/40 weeks gestation and/or <1500g	82%	Include
	32-36/40 weeks gestation and/or 1500-2500g	62%	Exclude
	Babies born en-route to the hospital	54%	Exclude
	Mother reports a non-measured fever	52%	Exclude
Major sign	Bilious vomiting with severe abdominal distension	88%	Include
'Other' sign/ symptoms	Irritable/inconsolable baby	64%	Exclude
	Do you think a one-off T <35.5 °C should be added as a sign of neonatal sepsis in a LIC?	44%	Exclude
Additional RF?	Swollen red eyelids with pus	88%	Include
	Unconscious	86%	Include, MCTW
	Central cyanosis	78%	Include (WHO danger sign)
	Poor capillary refill or perfusion	68%	Exclude
	Does the baby look ill?	60%	Exclude
**Consider Meningitis**	Drowsy, lethargic or unconscious with T >37.5^{0}C	96%	Include
	Bulging fontanelle with T >37.5^{0}C	94%	Include
	Irritability with a high-pitched cry with T >37.5^{0}C	92%	Include

	Abnormal movements/twitching or convulsions with T $>37.5^0$C	90%	Include
	Abnormal tone with T $>37.5^0$C	80%	Include
**HIE diagnosis**	Do you agree with absent suck and gestation $<32/40$ weeks as a sign of HIE?	42%	Exclude
	How should we describe "significant" birth injury as a risk factor for HIE?	20%	Exclude
**RDN diagnosis**	Do you agree with gestation $<34/40$ for part of the diagnostic criteria for Respiratory Distress Syndrome?	74%	Include, change to WHO definition
	Do you agree with coarse crackles (instead of unilateral crackles) for part of the diagnostic criteria for congenital pneumonia?	74%	Include, change to WHO wording
	Do you agree with T $>37.5^0$C or $<36.5^0$C for part of the diagnostic criteria for congenital pneumonia?	70%	Include, change to expert suggestion

HIE, Hypoxic Ischaemic Encephalopathy; LICs, low-income countries; MCTW, minor changes to wording; RDN, respiratory distress of the newborn; RF, risk factor; T, temperature; WHO, World Health Organisation

DISCUSSION

We report the use of a modified Delphi technique to review digital clinical pathway algorithms for four neonatal conditions managed by HCWs in LICs. Approximately two thirds (62%) of the original algorithm items were ultimately included for use in the NeoTree digital platform based on consensus expert opinion and national/international guidelines. The NeoTree team revised the algorithms based on this feedback. Expert discussion emphasised gaps in evidence in neonatal care in LICs, highlighting areas for future research.

Each algorithm had components that triggered debate amongst the experts. For neonatal sepsis, three points were discussed. First, experts called for further evidence before adopting a 'major' and 'minor' algorithmic weighting system to diagnose neonatal sepsis. In response, the NeoTree research team are conducting a study in Zimbabwe and Malawi looking at which clinical indicators are predictors of positive blood cultures. Second, there was disparity in opinion regarding whether to give prophylactic antibiotics and the duration of antibiotics for newborns with risk factors for sepsis who remain clinically well without any supporting investigations (NeoTree's equivalence to asymptomatic sepsis). The WHO recommendation to administer prophylactic antibiotics for a neonate with maternal risk factors for sepsis is considered weak

For peer review only

with very low-quality evidence.[22] Despite reaching a consensus on particular risk factors (prolonged rupture of membranes, maternal fever), experts also highlighted the evidence base as weak. In terms of duration of treatment for asymptomatic sepsis, while expert opinion varied, the Malawian guidelines recommend a five-day course[11] whilst WHO recommends two days.[15] The NeoTree algorithms will therefore keep to local and international recommendations, but the NeoTree team will feed back to the Malawian COIN expert panel that consensus suggested five days is too long to treat newborns with sepsis risk factors only. Third, experts disputed the treatment choice and duration for symptomatic neonatal sepsis; incidentally, WHO recommendations lack strong evidence or efficacy.[17]

For the HIE algorithm, the Thompson score was preferred because it is simpler to perform, less time consuming and better at predicting poor outcomes in moderate and severe HIE during the first hours of life compared to the Sarnat score at 24 hours.[19] The NeoTree research team suspected that measures such as examining for posturing and Moro reflex would be relatively complicated for frontline HCWs with minimal training to assess. However, neonatal experts' experience and previous studies in LICs[23] assured the team that the score is relatively straightforward to teach.

Several points of discussion also centred on the respiratory algorithms. First, experts noted that even with investigations in HICs respiratory conditions may be difficult to diagnose.[24] Second, despite experts' concerns about antibiotic overprescribing in LICs and the need to differentiate TTN from other respiratory conditions, they did not think this was currently feasible in LICs due to limitations in HCW capacity, resources and knowledge. Thus, experts agreed that all neonates with signs of respiratory distress should have respiratory support and antibiotics. A recent study justified the use of antibiotics for tachypnoea alone in a neonate in a resource-limited setting.[25]

For peer review only

Third, experts recommended performing chest x-rays (if available) only if imaging would change management (e.g. a longer course of antibiotics for congenital pneumonia) or if the neonate was deteriorating.

With the proliferation of clinical digital platforms in HICs and LICs, there is growing concern with the quality and safety standards of their clinical guidance. Countries and organisations (including WHO) are now taking measures to ensure application developers fulfil a strict set of criteria to protect patients.[26] While the Delphi technique can establish expert consensus, it may also strengthen the safety and quality standards of clinical algorithms. This technique has been widely used in developing paper-based neonatal clinical guidelines in HICs and LICs.[27-29] There are also studies that have used the Delphi technique to develop items used in m-health tools.[30-32] Our study is unique in the application of this technique to develop algorithms on a digital platform specific to neonatal care in low-resource settings.

This study has several limitations. The choice of using a modified two-step Delphi process meant that a final face-to-face meeting was not possible, which may have prevented some exchange of important information to clarify differences in expert opinion. However, this method allowed for the contributions of geographically dispersed experts, maintained their anonymity and prevented them from conforming to other experts. The recruitment of more experts from HICs (64%) compared to LICs (36%), despite originally inviting equal numbers to participate, could have contributed to expert panel bias. We invited three Malawian clinicians as experts; the absence of their input could be another limitation, since the algorithms have initially been contextualized to the Malawian setting. However, the end goal of NeoTree development is to be applicable in a wide range of resource-limited settings; therefore, experts with a broad geographical range of

For peer review only

clinical experience were recruited. Dropouts from the first to the second round could have affected the consensus level and contributed to attrition bias.

Some factors may have contributed to selection bias. The Delphi process is time intensive, which could have meant that those clinicians who are busier with perhaps even more clinical expertise or those with limited internet access (mainly LICs) could not participate. Additionally, offering a financial incentive might have obtained a more equal representation of experts. Another drawback of the Delphi being a labour-intensive process was that a year elapsed between the two rounds. Experts may have forgotten the algorithms and items from the first round in the second round if they did not read the summary of results or refresh their knowledge of the algorithms. Experts reported that they found the layout of the second questionnaire confusing; a re-design contributed to delays.

This study used the Delphi technique to refine four clinical decision algorithms in a neonatal digital platform designed for HCWs in LICs to standardise and improve the quality of newborn care. The key to implementing the NeoTree algorithms in other LICs will be to demonstrate that clinical algorithms in a digital platform versus paper-based guidelines can aid HCWs in making faster, more accurate diagnoses and provide better, more cost-effective treatment that will ultimately improve the quality of newborn care and reduce mortality. This will require a large-scale clinical-trial evaluation. Ultimately, with consensus opinion shaping the algorithms of this digital platform, accurate data capture, immediate clinical assessment and optimal medical care may be achieved to improve neonatal outcomes.

ACKNOWLEDGEMENTS

We thank all the participants of the Delphi surveys for their invaluable contributions to revise the clinical algorithms and highlight open questions, including Dr. Helen Brotherton, Dr.

For peer review only

Simbarashe Chimhuya, Dr. Cath Harrison, Dr. Tyler Hartmann, Dr. Hammad Khan, Dr. Camilla Kindgon, Dr. Tom Lissauer, Dr. Azza Mashumba, Kathy Mellor (MBE), Professor Elizabeth Molyneux, Dr. Cally Tann, Dr. Cliff O'Callahan, Susan Quinton, and Dr. Raissa Teteli. We would also like to thank Dr. Brian Corden and Dr. Alice Myers for their reviews of the manuscript.

CONTRIBUTORS

ME, CC and MH conceived the study, generated the methodology and designed the questionnaires. ME conducted the interviews and analysed the data. ME and MC produced the first draft and contributed equally to this manuscript. CC, FF and MH provided edits and comments to the draft. All authors reviewed and approved the final version of the manuscript.

FUNDING

This work was supported in part by the Wellcome Trust Digital Innovation Award (215742/z19/z). FF is supported by the Academy of Medical Sciences, the funders of the Starter Grant for Clinical Lecturers scheme and the UCL Great Ormond Street NIHR Biomedical Research Centre. The funders had no role in the study design, data collection and analysis, or preparation of the manuscript.

COMPETING INTERESTS

CC and MH are co-founders of the NeoTree platform and continue to conduct research related to its development. FH and MH are trustees of the NeoTree Foundation. The NeoTree platform is a not-for-profit product; none of the co-authors benefit financially.

ETHICS APPROVAL

Ethics approval was not required for this study according to the University College of London Research Ethics Committee.

For peer review only

PROVENANCE AND PEER REVIEW

Not commissioned; externally peer reviewed.

DATA SHARING STATEMENT

All data relevant to the study are included in the article, uploaded as supplementary information or available upon request from the corresponding author.

PATIENT CONSENT FOR PUBLICATION

Not required.

ORCID IDs

Mari Evans https://orcid.org/0000-0001-5113-3629

Mark Corden https://orcid.org/0000-0001-6146-1634

Caroline Crehan https://orcid.org/0000-0002-3655-6954

Felicity Fitzgerald https://orcid.org/0000-0001-9594-3228

Michelle Heys https://orcid.org/0000-0002-1458-505X

For peer review only

REFERENCES

1 WHO. 2019. Newborns: reducing mortality. https://www.who.int/news-room/fact-sheets/detail/newborns-reducing-mortality [Accessed May 2020].

2 Lawn JE, Blencowe H, Oza S, et al. Every Newborn: progress, priorities, and potential beyond survival. *Lancet* 2014;384(9938):189–205.

3 WHO. 2019. Levels & trends in child mortality: report 2019. Estimates developed by the UN Inter-agency Group for Child Mortality Estimation. https://www.unicef.org/reports/levels-and-trends-child-mortality-report-2019 [Accessed May 2020].

4 WHO. 2015. WHO Guidelines Approved by the Guidelines Review Committee. https://www.who.int/publications/guidelines/guidelines_review_committee/en/ [Accessed June 2019].

5 WHO. 2017. WHO recommendations on newborn health: guidelines approved by the WHO Guidelines Review Committee. https://www.who.int/publications/i/item/WHO-MCA-17.07 [Accessed February 2021].

6 Becker JU, Theodosis C, Jacob ST, et al. Surviving sepsis in low-income and middle-income countries: new directions for care and research. *Lancet Infect Dis* 2009;9(9):577–82.

7 TDV Global. 2016. Evaluation of the Impact of WHO Publications. https://www.who.int/docs/default-source/documents/evaluation/report-evaluation-impact-who-publications.pdf?sfvrsn=577627c5_2 [Accessed February 2021].

8 Hall CS, Fottrell E, Wilkinson S, et al. Assessing the impact of mHealth interventions in low- and middle-income countries–what has been shown to work? *Glob Health Action* 2014;7:25606.

For peer review only

9 Crehan C, Kesler E, Nambiar B, et al. The NeoTree application: Developing an integrated mHealth solution to improve quality of newborn care and survival in a district hospital in Malawi. *BMJ Glob Health* 2019;4:e000860.

10 WHO. 2013. Pocket book of hospital care for children: guidelines for the management of common childhood illnesses, 2nd ed. https://apps.who.int/iris/handle/10665/81170 [Accessed June 2018].

11 COIN Course, 2017. Care of the infant and newborn in Malawi. The COIN Course Participants Manual. Available from: http://cms.medcol.mw/cms_uploaded_resources/41905_12.pdf [Accessed June 2018].

12

12 Sinha IP, Smyth RL, Williamson PR. Using the Delphi technique to determine which outcomes to measure in clinical trials: recommendations for the future based on a systematic review of existing studies. *PLoS Med* 2011;8(1):e1000393.

13 Hsu CC, Sandford BA. The Delphi Technique: Making Sense of Consensus. *Practical Assessment, Research and Evaluation* 2007;12(10). https://scholarworks.umass.edu/pare/vol12/iss1/10 [Accessed July 2018].

14 Akins RB, Tolson H, Cole BR, et al. Stability of response characteristics of a Delphi panel: application of bootstrap data expansion. *BMC Med Res Methodol* 2005;5:37.

15 WHO. 2012. Recommendations for management of common childhood conditions: evidence for technical update of pocket book recommendations: newborn conditions, dysentery, pneumonia, oxygen use and delivery, common causes of fever, severe acute malnutrition and supportive care.

For peer review only

https://apps.who.int/iris/bitstream/handle/10665/44774/9789241502825_eng.pdf?sequence=1 [Accessed June 2019].

16 European Medicines Agency. 2010. Report on the Expert Meeting on Neonatal and Paediatric Sepsis. https://www.ema.europa.eu/en/documents/report/report-expert-meeting-neonatal-paediatric-sepsis_en.pdf [Accessed July 2019].

17 Obiero CW, Seale AC, Berkley JA. Empiric treatment of neonatal sepsis in developing countries. *Pediatr Infect Dis J* 2015;34(6):659–661.

18 Babakus E, Mangold WG. Adapting the SERVQUAL scale to hospital services: an empirical investigation. *Health Serv Res* 1992;26(6):767–786.

19 Thompson CM, Puterman AS, Linley LL, et al. The value of a scoring system for hypoxic ischaemic encephalopathy in predicting neurodevelopmental outcome. *Acta Paediatr* 1997;86(7):757–761.

20 Ballard JL, Khoury JC, Wedig K, et al. New Ballard Score, expanded to include extremely premature infants. *J Pediatr* 1991;119(3):417–23.

21 Sarnat HB, Sarnat MS. Neonatal encephalopathy following fetal distress: a clinical and electroencephalographic study. *Arch Neurol* 1976;33(10):696–705.

22 Ungerer RL, Lincetto O, McGuire W, et al. Prophylactic versus selective antibiotics for term newborn infants of mothers with risk factors for neonatal infection. *Cochrane Database Syst Rev* 2004;4:CD003957.

22

23 Bhagwani DK, Sharma M, Dolker S, et al. To study the correlation of Thompson scoring in predicting early neonatal outcome in post asphyxiated term neonates. *J Clin Diagn Res* 2016;10(11):SC16-SC19.

For peer review only

24 Costa S, Rocha G, Leitao A, et al. Transient tachypnea of the newborn and congenital pneumonia: a comparative study. *J Matern Fetal Neonatal Med* 2012;25(7):992–4.

25 Tikmani SS, Muhammad AA, Shafiq Y, et al. Ambulatory treatment of fast breathing in young infants aged <60 days: A double-blind, randomized, placebo-controlled equivalence trial in low-income settlements of Karachi. *Clin Infect Dis* 2017;64(2):184–189.

26 van Haasteren A, Gille F, Fadda M, et al. Development of the mHealth App Trustworthiness checklist. *Digit Health* 2019;5:1–21

27 Kamath-Rayne BD, Thukral A, Visick MK, et al. Helping Babies Breathe, Second Edition: A Model for Strengthening Educational Programs to Increase Global Newborn Survival. *Glob Health Sci Pract* 2018;6:538–551.

28 Balikuddembe MS, Tumwesigye NN, Wakholi PK, et al. Expert perspectives on essential parameters to monitor during childbirth in low resource settings: a Delphi study in sub-Saharan Africa. *Reprod Health* 2019;16(1):119.

29 Housseine N, Punt MC, Browne JL, et al. Delphi consensus statement on intrapartum fetal monitoring in low-resource settings. *Int J Gynaecol Obstet* 2018;146(1):8–16.

30 Bakibinga P, Kamande E, Omuya M, et al. The role of a decision-support smartphone application in enhancing community health volunteers' effectiveness to improve maternal and newborn outcomes in Nairobi, Kenya: quasi-experimental research protocol. *BMJ Open* 2017;207(7):e014896.

31 Fung JST, Akech S, Kissoon N, et al. Determining predictors of sepsis at triage among children under 5 years of age in resource-limited settings: A modified Delphi process. *PLoS One* 2019;14(1):e0211274.

For peer review only

32 Pellé KG, Rambaud-Althaus C, D'Acremont V, et al. Electronic clinical decision support algorithms incorporating point-of-care diagnostic tests in low-resource settings: a target product profile. *BMJ Glob Health* 2020;5:e002067.

For peer review only

FIGURE CAPTIONS

Figure 1. Outcome of algorithm items after round one and round two of the Delphi technique.

Figure 2. Modification of the algorithms as a result of the Delphi technique.

For peer review only

Figure 1. Outcome of algorithm items after round one and round two of Delphi technique.

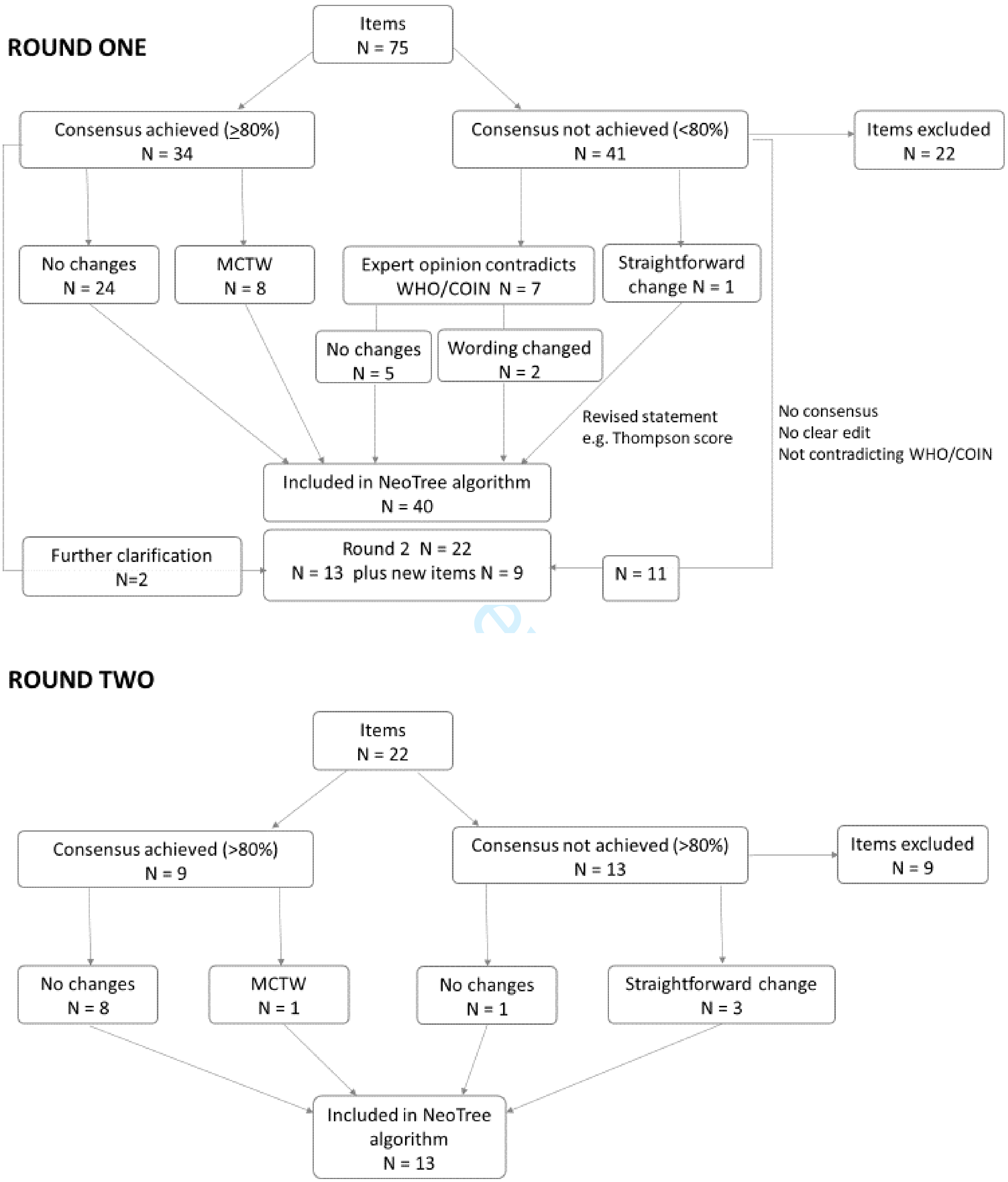

Abbreviations: COIN = Care of the Infant and Newborn in Malawi; MCTW = minor changes to wording; WHO = World Health Organisation

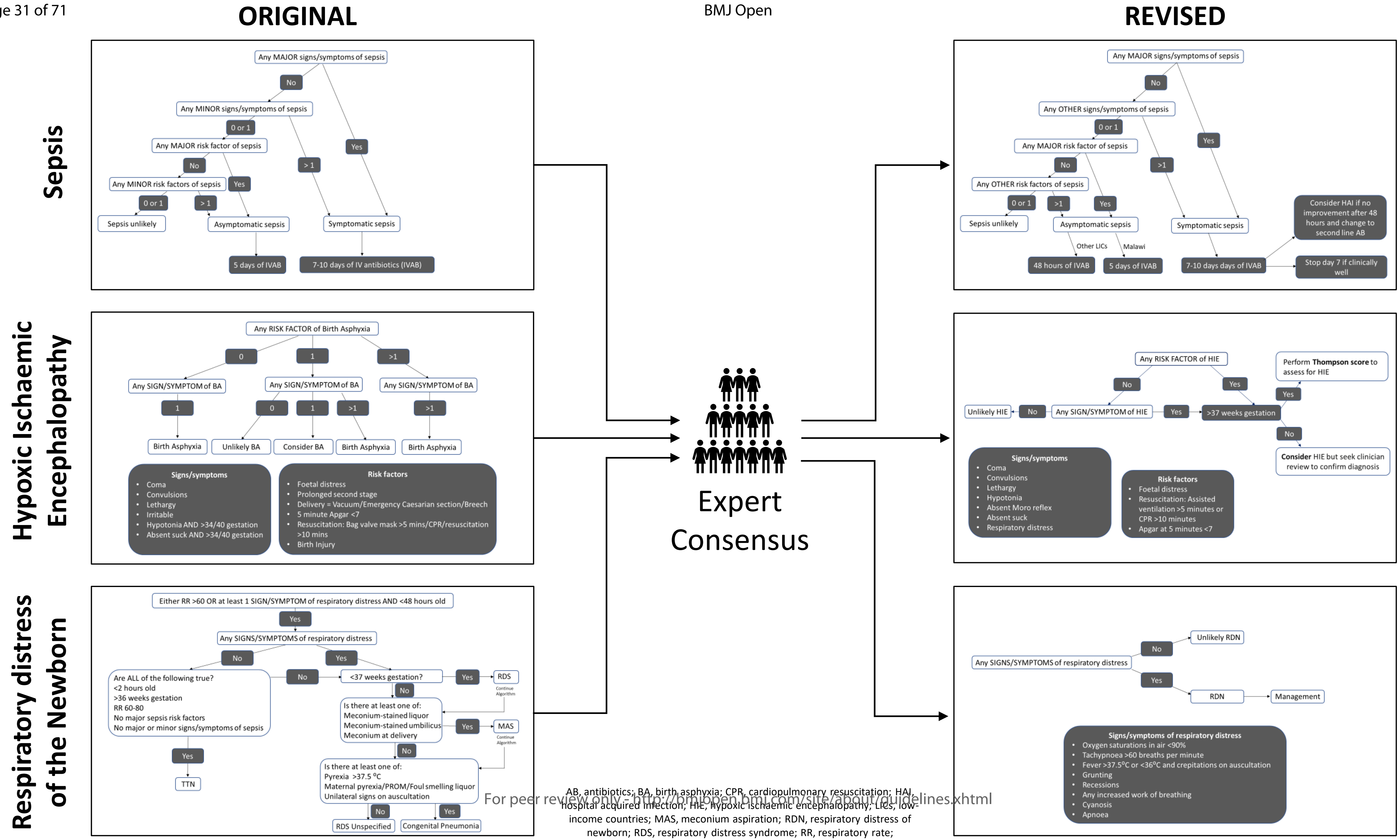

AB, antibiotics; BA, birth asphyxia; CPR, cardiopulmonary resuscitation; HAI, hospital acquired infection; HIE, hypoxic ischaemic encephalopathy; LICs, low-income countries; MAS, meconium aspiration; RDN, respiratory distress of newborn; RDS, respiratory distress syndrome; RR, respiratory rate; TTN, transient tachypnoea of newborn

Round one questionnaire

NeoTree questions on algorithms for expert review

Prior to each of these algorithms the user of the NeoTree must complete the emergency triage section. This section ensures that the baby is resuscitated as per Helping Babies Breathe, guiding the user to check for danger signs. Any abnormal parameters entered trigger immediate prompting to give appropriate resuscitation before addressing these diagnoses. Included in this will be a blood sugar measurement.

Section 1. Neonatal Sepsis

Summary of sepsis algorithm

For this part of the algorithm please assume you have the following equipment/resources:

- the NeoTree application on a tablet device
- a pulse oximeter
- a thermometer
- a clock
- a stethoscope
- oxygen
- bubble CPAP

Please assume, however, that you are working in an environment where there is no availability for blood cultures, inflammatory markers, white cell count, or chest x-ray. Lumbar puncture is available for gram stain, protein and cell count only. In the future these investigations may become more widely available in newborn facilities, and there is the potential to add these extra parameters to the NeoTree algorithm. But currently, when we say 'sepsis' we mean presumed sepsis that cannot be confirmed with laboratory investigations.

Within the NeoTree algorithm, the diagnosis of sepsis is divided into 4 categories according to the presence or absence of signs/symptoms and/or risk factors and the age of the newborn (Flowchart 1). In order to account for different levels of risk we have weighted both signs/symptoms and risk factors as 'MAJOR' or 'MINOR' according to COIN / WHO / best judgement. A MAJOR sign/symptom is weighted twice as heavily as a MINOR one, so that, for example, in the diagnosis of symptomatic sepsis either 1 MAJOR sign/symptom or 2 MINOR signs/symptoms will trigger the diagnosis. For asymptomatic sepsis only, risk factors are required. We have used a similar weighting system for risk factors.

For peer review only

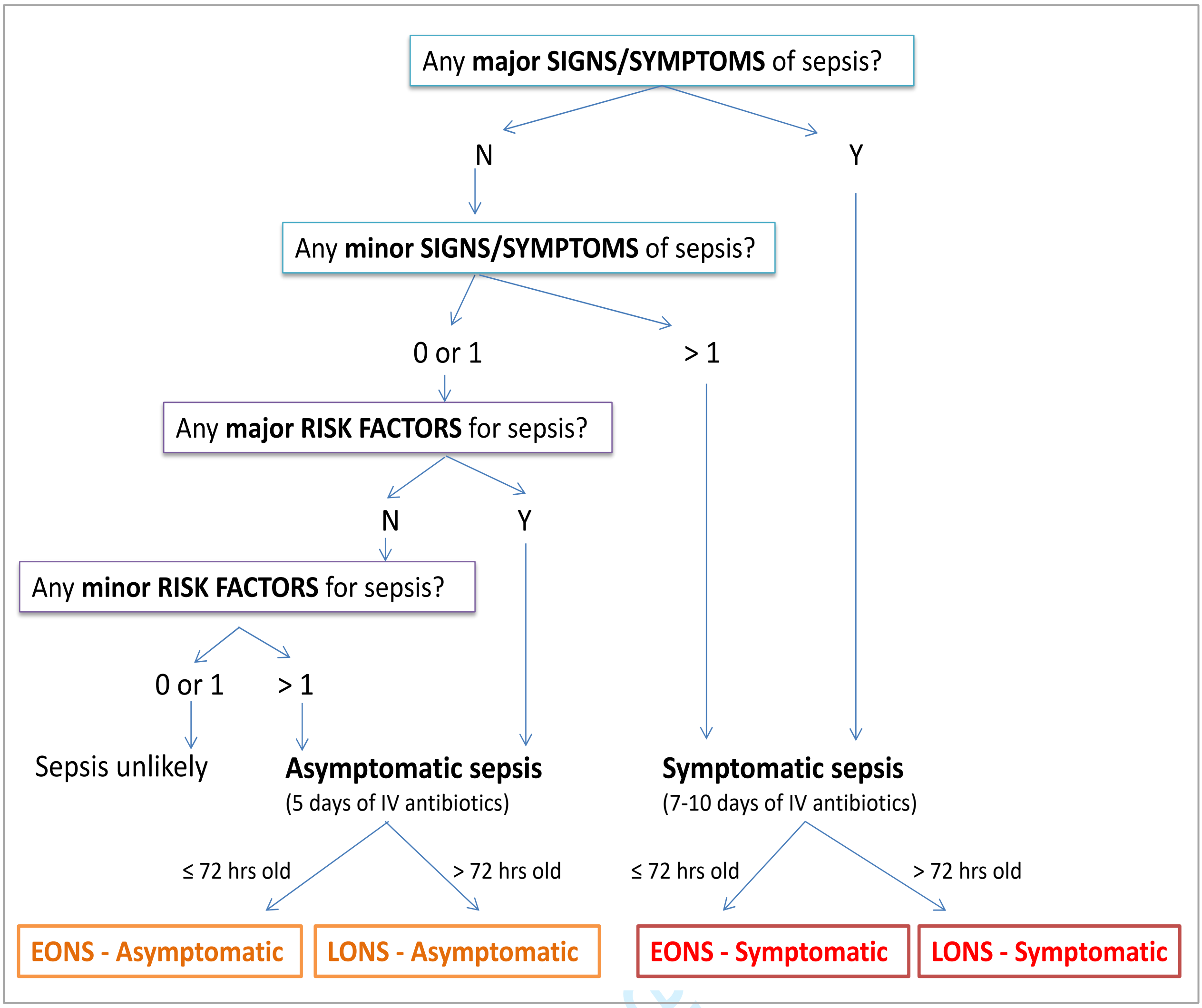

EONS = Early onset neonatal sepsis, LONS = Late onset neonatal sepsis

Flowchart 1: Categories of sepsis are divided by presence (symptomatic) or absence (asymptomatic) of signs/symptoms and by age of newborn

For peer review only

Table 1: Risk factors and signs/symptoms of sepsis

MAJOR risk factors	MINOR risk factors
1. Maternal fever >38°C in labour *+<br>2. Offensive liquor*+<br>3. Prolonged rupture of membranes (PROM) >18 hrs*+<br>4. Prolonged second stage (>3 hrs of active pushing)*<br>5. Very / extremely premature (<32/40 weeks gestation)~	1. Born before arrival (BBA)~<br>2. Prematurity (32-36/40 weeks gestation) (N.B in the app you can only round to the closest week i.e 36 or 37 weeks)~
**MAJOR signs/symptoms**	**MINOR Signs/symptoms**
1. Temperature >37.5°C*<br>2. Pustules all over body*+<br>3. Boil/abscess*+<br>4. Red skin all around umbilicus*+<br>5. Bulging fontanelle*+<br>6. Grunting or severe respiratory distress/mod-severe work of breathing*+<br>7. History of apnoea~<br>8. Jaundice <24 hrs old+<br>9. Tachypnoea >60*+ (except 60-80 bpm at <2hrs old)~<br>10. Lethargy+<br>11. Convulsions+<br>12. Bilious vomiting~	1. Mild work of breathing~<br>2. Tachycardia (HR>160) that can't be explained by fever or crying~<br>3. Tachypnoea 60-80 bpm*+ at <2hrs old~<br>4. Pallor+<br>5. Abdominal distension~<br>6. Poor feeding/vomiting+<br>7. Weak or absent suck (AND gestation >34 weeks)~<br>8. Irritability*+

bpm = breaths per minute, HR = heart rate

Notes: *Source/Evidence used: *COIN, +WHO, ~NeoTree team best judgement*

Consider Meningitis:

For the following scenarios the NeoTree will ask the health care worker (HCW) to consider meningitis:

- Temp >37.5°C* AND any of
  - Bulging Fontanelle*+
  - Hypertonia+
  - Persistent irritability*+
  - Lethargy*+

OR

- Convulsions*+ / History of convulsions*
- Hydrocephalus (obviously enlarged head or OFC >37cm)~

Notes: *Source/Evidence used: *COIN, +WHO, ~NeoTree team best judgement*

Explanatory notes for Sepsis algorithm:

- Regarding work of breathing, for the sake of simplicity the NeoTree algorithm asks the user to observe the work of breathing and rate it as mild, moderate or severe with the aid of educational videos on the app. There is the potential to include a validated respiratory distress score in future iterations.

- Hypothermia has not been included as a sign of sepsis due to the high rates of environmental hypothermia on admission in these settings. Persistent hypothermia despite warming may be included in future iterations.

For peer review only

- We have separated mild tachypnoea of 60-80 in ages <2 hours into the minor signs/symptoms so that if it exists without any other signs/symptoms or risk factors it can have the diagnosis of transient tachypnoea of the newborn rather than sepsis.

Sepsis diagnosis questions:

**Table 2: Sepsis diagnosis questions**		
**Question**	**Disagree → Agree**	**Discuss responses**
**1. Do you agree with the following MAJOR risk factors?**		
a. Maternal fever >38 °C in labour<br>b. Offensive liquor<br>c. PROM >18 hours<br>d. Prolonged second stage (>3 hours of active pushing)<br>e. Very / extremely premature (<32/40 weeks gestation)	1 2 3 4 5<br>1 2 3 4 5<br>1 2 3 4 5<br>1 2 3 4 5<br>1 2 3 4 5	
**2. Do you agree with the following MINOR risk factors?**		
a. Born before arrival (BBA)<br>b. Prematurity (32-36/40 weeks gestation)	1 2 3 4 5<br>1 2 3 4 5	
**3. Do you agree with the following MAJOR signs/symptoms?**		
a. Temperature >37.5 °C<br>b. Admitted with or history of fever<br>c. Pustules all over body<br>d. Boil/abscess<br>e. Red skin all around umbilicus<br>f. Bulging fontanelle<br>g. Grunting or severe respiratory distress or mod-severe WOB<br>h. History of apnoea<br>i. Jaundice <24 hours old<br>j. Tachypnoea >60 bpm **(except 60-80 & < 2 hours old)**<br>k. Lethargy<br>l. Convulsions<br>m. Bilious vomiting	1 2 3 4 5<br>1 2 3 4 5<br>1 2 3 4 5<br>1 2 3 4 5<br>1 2 3 4 5<br>1 2 3 4 5<br>1 2 3 4 5<br>1 2 3 4 5<br>1 2 3 4 5<br>1 2 3 4 5<br>1 2 3 4 5<br>1 2 3 4 5<br>1 2 3 4 5<br>1 2 3 4 5	
**4. Do you agree with the following MINOR signs/symptoms?**		
a. Mild work of breathing<br>b. Tachycardia that can't be explained by fever/crying<br>c. Pallor<br>d. Distended abdomen<br>e. Poor feeding/vomiting<br>f. Weak or absent suck (AND gestation >34 weeks)<br>g. Irritability<br>h. Tachypnoea 60-80 bpm & <2 hrs old	1 2 3 4 5<br>1 2 3 4 5<br>1 2 3 4 5<br>1 2 3 4 5<br>1 2 3 4 5<br>1 2 3 4 5<br>1 2 3 4 5<br>1 2 3 4 5	
**5. Are there any additional risk factors, signs or symptoms that you think should be included for a low-income setting? For example…**		

For peer review only - http://bmjopen.bmj.com/site/about/guidelines.xhtml

a. Hypothermia <35.5 °C<br>b. Joint swelling (NB NeoTree is more immediate mx).<br>c. Reduced movement of limbs	Y/N, if yes Maj/Minor<br>Y/N, if yes Maj/Minor<br>Y/N, if yes Maj/Minor	
**6. If you have not already stated. Should any of the risk factors or signs and symptoms be MAJOR or MINOR?**		
**7. Do you agree with the cut off from 72 hours for early and late neonatal sepsis?**	1 2 3 4 5	
**8. Do you agree with the definition of maternal fever being >38 degrees °C?**	1 2 3 4 5	
**9. Should PROM be >18 hours or >24 hours in this setting?**	18 hours = 1<br>24 hours = 5	
**10. Do you agree that fever in a newborn should be classified as >37.5 °C in this setting?**	1 2 3 4 5	
**11. Do you agree with the cut- off at 34 weeks gestation for the 'no suck' in a non-prem sepsis sign?**	1 2 3 4 5	
**12. Please comment on our weighting system of Major = 100%, Minor = 50%**		
**13. Please comment on the criteria for 'Consider meningitis'**		

For peer review only

Management of Sepsis:

Box 1. Management page for Sepsis in NeoTree:

IV access
Get IV access
<u>If possible,</u> perform sepsis screen
Sepsis screen: FBC, CRP, Blood Culture, +/- Lumbar Puncture (CRP is C-reactive protein a marker of infection available in some labs)

Antibiotics
Start antibiotics according to local guidelines. If no guidelines give:
-Penicillin 50,000 iu/kg 12 hourly IV/IM and
-Gentamycin 3 or 5 mg/kg/dose 24 hourly IV/IM (LBW or Term)
Length of antibiotics treatment:
a) Asymptomatic sepsis, treat for 5 days
b) Symptomatic sepsis, treat for 7-10 days
Frequency of penicillin is 6 hourly if >7 days age
Use Flucloxacillin and Gentamycin if skin infection or pustules

Supportive care
Thermoregulation: aim for temperature between 36.5 - 37.5 °C
If baby has fever remove from warmer and unwrap
Provide oxygen or CPAP as needed according to TRY CPAP algorithm
Check blood glucose and provide feeding support as needed

- Consider 10ml/kg fluid bolus if shocked as per SHOCK algorithm
- Consider 2.5mls/kg 10% dextrose if BS <2.5 mmol or 45 mg/dl – as per HYPOGLYCAEMIA algorithm

*Source/Evidence used: *COIN*

- The NeoTree antibiotic management guidelines for sepsis follows the Malawi COIN guidelines. The NeoTree recommends 5 days of IV antibiotics for asymptomatic sepsis and 7-10 days for symptomatic sepsis.

Sepsis management questions:

Table 3. Sepsis management questions		
**Question**	**Disagree → Agree**	**Discuss responses**
**1. Do you agree with the specified sepsis investigations if possible?**	1 2 3 4 5	
**2. Do you agree with the antibiotic choices and doses if no local recommendation?**	1 2 3 4 5	
**3. Do you agree with antibiotic duration?**<br>a) Asymptomatic sepsis = 5 days<br>b) Symptomatic sepsis = 7-10 days	 1 2 3 4 5<br>1 2 3 4 5	

For peer review only

Section 2. Birth Asphyxia

Summary of Birth Asphyxia algorithm:

We have divided the diagnosis of birth asphyxia into 2 categories according to the presence or absence of risk factors or signs/symptoms in Table 4:

A. ***Consider birth asphyxia*** = 1 risk factor AND 1 sign/symptom of birth asphyxia
B. ***Birth asphyxia*** = either
2 or more risk factors AND 1 sign/symptom OR
No known risk factors AND 2 or more signs/symptoms

Table 4. Risk factors, signs and symptoms for birth asphyxia

Risk factors	Signs/Symptoms
• Foetal distress*<br>• Prolonged second stage*<br>• Delivery = Vacuum / EmCS / Breech~<br>• Apgar at 5 mins <7+<br>• Resuscitation: BVM for >5 mins* / CPR* / resuscitated for >10 mins~<br>• Birth injury~	• Absent suck AND >34/40 gestation~<br>• Hypotonia AND >34/40 gestation~<br>• Irritable*+<br>• Lethargy*+<br>• Coma*+<br>• Convulsions*+

EmCS = Emergency caesarian section, BVM = Bag valve mask ventilation, CPR = Cardio pulmonary resuscitation, BW = birth weight.

Evidence/Source used: *COIN, +WHO, ~NeoTree team best judgement

For peer review only

Birth asphyxia diagnosis questions:

Table 6: Birth asphyxia diagnosis questions

Questions	Disagree → Agree	Discuss responses
**1. Do you agree with the diagnostic criteria for:**		
A. 'Consider birth asphyxia'<br>B. 'Birth asphyxia'	1 2 3 4 5<br>1 2 3 4 5	
**2. Do you agree with the following risk factors for birth asphyxia?**		
a. Foetal distress<br>b. Prolonged second stage >3 hours duration<br>c. Delivery = Vacuum / EmCS / breech<br>d. Apgar at 5 mins <7<br>e. Resuscitation: BVM >5 mins / CPR lasted >10 mins<br>f. Birth injury	1 2 3 4 5<br>1 2 3 4 5<br>1 2 3 4 5<br>1 2 3 4 5<br>1 2 3 4 5<br>1 2 3 4 5	
**3. Do you agree with the following signs/symptoms for birth asphyxia?**		
a. Absent suck and gestation >34/40<br>b. Hypotonia and gestation >34/40<br>c. Irritable<br>d. Lethargy<br>e. Coma<br>f. Convulsions	1 2 3 4 5<br>1 2 3 4 5<br>1 2 3 4 5<br>1 2 3 4 5<br>1 2 3 4 5<br>1 2 3 4 5	
4. Should birth asphyxia be classified as mild, moderate or severe?	1 2 3 4 5	
**5. Do you think the following sign/symptoms and risk factors should be included?**		
a. Poor feeding<br>b. Respiratory distress<br>c. >4kg	Y/N<br>Y/N<br>Y/N	
6. Are there any other risk factors or signs/symptoms to be added/removed?		
7. Due to the difficulties of training HCW in checking safely for the Moro reflex in these settings we have removed from the assessment. Do you agree?	1 2 3 4 5	

For peer review only

Box 2: Management page for Birth Asphyxia in NeoTree:

1. **Keep warm**
   KMC/radiant warmer

   (Unless the patient is hyperthermic)

2. **Respiratory support**
   Put on appropriate respiratory support

3. **Feeding support, anticonvulsants and antibiotics**
   Mild/Moderate:
   - Give NG feeds- most will have a poor suck reflex
   - Mother can still offer breast to develop the suck reflex
   - Check blood sugar (BS) and give 10% dextrose bolus if BS <2.5mmol/L or <45mg/dL

   Severe: where NG feeds are not tolerated, consider IV fluids.

If evidence of seizure activity give **PHENOBARIBITONE** as per convulsions page.

Reassess suck, tone, coma, seizures every 8 hours for 72 hours in hospital

Treat for sepsis with antibiotics

Convulsions page in NeoTree:

- Load phenobarbitone 20 mg/kg
- If seizures ongoing can reload twice
- Start 5 mg/kg maintenance (24 hours after loading dose)
- Stop phenobarbitone at 72 hours if no further seizures and neurologically normal

*Source/Evidence used: *COIN*

Management of Birth Asphyxia questions:

Table 7. Management of Birth Asphyxia questions

Questions	Disagree → Agree	Discuss responses
Do you agree not to do passive cooling for infants in low resource settings?	1 2 3 4 5	
Should the newborn be given IV fluids if not tolerating oral or NG feeds?	1 2 3 4 5	

Section 3. Respiratory distress of the newborn (RDN)

Summary of the RDN algorithm:

For this part of the algorithm please assume you have the following equipment/resources:

- the NeoTree application on a tablet device
- a pulse oximeter
- a thermometer
- a clock
- a stethoscope
- oxygen
- bubble CPAP

Please assume, however that you do not have chest x-ray available to you. We have divided this diagnosis into the following 4 categories:

1. Meconium aspiration

2. Respiratory Distress Syndrome (RDS)

3. Congenital pneumonia

4. Transient Tachypnoea of Newborn

Flowchart 2: RDN diagnostic algorithm

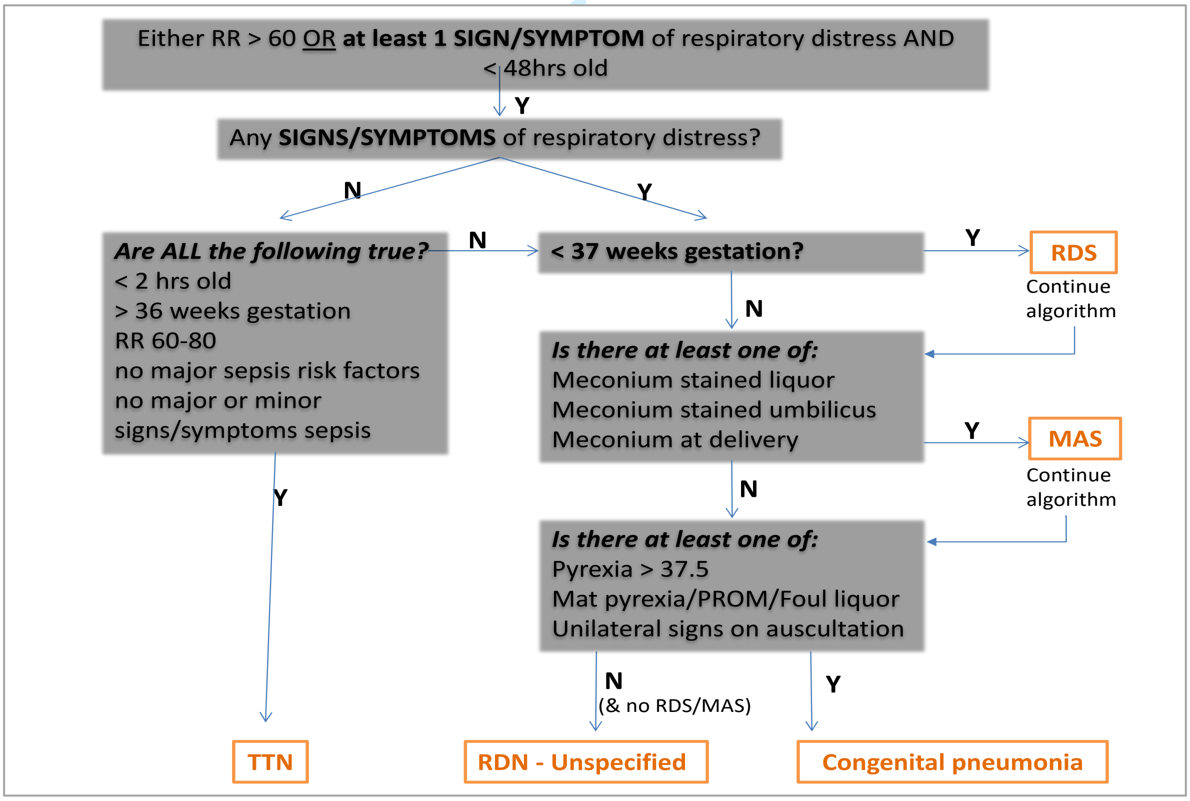

MAS = Meconium aspiration, PROM = prolonged rupture of membranes, RDN = Respiratory Distress of Newborn, RDS = Respiratory distress syndrome, RR = Respiratory rate, TTN = Transient Tachypnoea of Newborn, Y= Yes, N = No.

Table 8. Signs/ Symptoms for RDN
• Grunting or severe recessions or moderate-severe work of breathing*<br>• Blue~ / cyanotic*<br>• Apnoea~<br>• History of fast or laboured breathing+<br>• History of noisy breathing~<br>• Saturations in air or oxygen <90%~<br>• Bilateral wheeze or crackles on auscultation+<br>• History of cough (only included in algorithm for congenital pneumonia)~

Source/Evidence used: *COIN, +WHO, ~NeoTree team best judgement

RDN diagnosis questions:

Table 9: RDN diagnostic algorithm questions

**Questions**	**Disagree → Agree**	**Discuss responses**
1. Do you agree that on admission a 'history' of fast or laboured breathing or noisy breathing is relevant as a sign or symptom of RDN when not present on admission?	1 2 3 4 5	
2. Do you agree that tachypnoea of 60 - 80bpm <2 hours old without signs or symptoms of sepsis should be treated as TTN, and no antibiotics given?	1 2 3 4 5	
3. Do you agree with tachypnoea of >60 for the other categories of RDN	1 2 3 4 5	
4. Are there any other signs/symptoms to be removed or to be considered?		
5. Do you agree with the diagnostic criteria for:<br>a) MAS?<br>b) RDS?<br>c) TTN?<br>d) Congenital pneumonia?	 1 2 3 4 5<br>1 2 3 4 5<br>1 2 3 4 5<br>1 2 3 4 5	
6. Are there any other risk factors to be considered? For example: Diabetic mother for MAS and RDS		

RDN Management:

We have presented the management for all 4 types of Respiratory distress of the newborn in one management page; however, in reality they each get an individual page in the app. NB. RDN – unspecified would be treated as per the 'GENERIC' section.

For peer review only

Box 3: Management page for Respiratory Distress of the Newborn (RDN):

Includes generic advice common to all 4 sub-diagnoses. Relevant disease specific advice is also included and specified below

GENERIC:
Airway and respiratory support
Position airway & suction
If saturations <90% in air put on oxygen+
If >1kg consider CPAP according to TRY algorithm~
Feeding support
If breathing 60-80 bpm use cup/NGT
If needing CPAP use OGT
If breathing >80bpm consider IV fluids
IV Access for Antibiotics
Give antibiotics (except if TTN)

MECONIUM ASPIRATION~
Think – is this really meconium aspiration?
Just because there was meconium at delivery doesn't mean it was aspirated.
Most aspiration occurs in utero before the baby is born, so more likely if there was foetal distress in the labour, if the baby is sick with respiratory distress or has signs of birth asphyxia. Floppy babies with severe HIE and meconium aspiration are unlikely to benefit from CPAP.

TTN~
These babies are not unwell
RR is usually between 60-80 bpm and they don't usually need oxygen
This should resolve in the first few hours of life.
TTN is more likely following elective C-section due to wet lungs
Does not need antibiotics unless tachypnoea persists.

RDS~
Consult local guidelines for how to give CPAP

CONGENITAL PNEUMONIA~
Before commencing antibiotics consider a full sepsis screen but don't let this delay giving antibiotics.

*Source/Evidence used: *COIN, +WHO, ~NeoTree team best judgement*

Management of RDN questions:

Table 10: Management of RDN questions		
**Questions:**	**Disagree → Agree**	**Discuss responses**
1. Do you agree with a cut off of 90% oxygen saturations before giving oxygen?	1 2 3 4 5	
2. Do you agree to give antibiotics in all cases except TTN?	1 2 3 4 5	
3. Do you agree with the time cut-off of at 2 hours for TTN? Would you have a higher or lower threshold?	1 2 3 4 5	
4. If it is possible to perform a CXR when and how would you recommend?		
5. Please give any other comments or opinions of the above.		

For peer review only

Section 4. Hypothermia

Box 4: Hypothermia management page in NeoTree:

Mild = 36 - 36.4°C+*

Mod = 32 - 35.9°C+*

Severe = <32°C+*

1. **Warm the baby**
   Mild: Skin to skin (KMC position)+*
   Moderate /severe: place on warmer/resuscitaire~

2. **Limit heat loss**
   Make sure the baby is dry+*
   Put on a hat and wrap up the baby+*
   If on the resuscitaire put the sides up~
   Ensure room is free from drafts- windows and doors closed+*
   The 4 ways by which a baby can lose heat are:+*

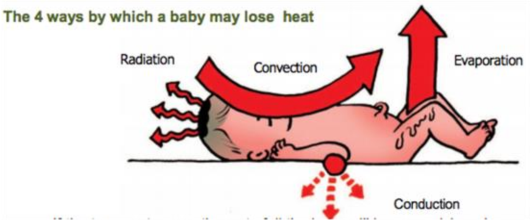

3. **Monitor**
   Measure temperature every 15-30 minutes~
   Watch out for overwarming+*
   Complete NeoTree assessment to assess for signs of infection, apnoea and hypoglycaemia*

Evidence/Source: *COIN, +WHO, ~NeoTree team best judgement

Hypothermia questions:

We have not included an algorithm for hypothermia here, as it is simply the diagnostic criteria stated at the top of the thermoregulation management page.

Table 11: Hypothermia questions		
**Questions**	Disagree → Agree	Discuss responses
1. Do you agree with the diagnostic criteria for hypothermia?	1 2 3 4 5	
2. Should we add more detail on ongoing management?	1 2 3 4 5	
3. Any other comments?	1 2 3 4 5	

References

http://www.who.int/maternal_child_adolescent/documents/child_hospital_care/en/WHO. 2013. Pocket book of hospital care for children: Guidelines for the management of common childhood illnesses

cms.medcol.mw/cms_uploaded_resources/41905_12.pdf
Ministry of Health. 2017. Care of the infant and newborn in Malawi The COIN Course Participants Manual.

For peer review only

Round two questionnaire

For items that did not reach a consensus (<80% agreement) the questions have been:

1. Removed
2. Kept because part of WHO or Malawian neonatal (COIN) guidelines
3. Changed and submitted below for a second round

For items with a consensus (>80% agreement) we have kept or changed to WHO wording on expert advice.

Please score the new items using the Likert scale of 1 (strongly disagree) to 5 (strongly agree) and provide comments for those items that you disagree with. Please read expert responses from round 1 for each item to aid your response for the second round.

Section 1. Neonatal Sepsis

Sepsis diagnosis questions

We did not reach a consensus on our major and minor weighting system approach to diagnosing neonatal sepsis, and we cannot come to a decision until we have done our clinical validation study and collected data from Zimbabwe study using blood cultures to identify which risk factors are most strongly predictive of sepsis. However, we would still like to explore the usefulness for 'other' risk factors to be included alongside the WHO and COIN danger signs.

1. Do you agree with the following 'other' risk factors for neonatal sepsis?

A. Born before arrival (BBA)

Expert responses

I would never consider this a risk factor in a high resource setting for sepsis but in a low resource setting where you have no clue where or how the baby was born, what equipment was used and what the circumstances where I can see this as a risk factor.

I don't know this as a risk factor. I hesitate because there is so much controversy with that statement and we had a lot of discussion about this in Rwanda.

I guess there is then uncertainty with all the maternal risk factors. So yes, I agree but I don't think there is any evidence behind it but I can see the logic.

It depends where. Yes, is they were born at home and un-booked mothers who have not had had antenatal care but not if born in another facility.

Depends on where they were born and who delivered/cut the cord

It depends where the baby has been born. Quite a lot of babies are born outside the hospital and that would be mean every baby would need antibiotics.

I disagree. Is this to the hospital? I will have to disagree here because depending on where you live most babies do not even come to the hospital.

Traditionally this is what people have done but I don't get very excited about it and I advise to follow the WHO guidelines.

For peer review only

Original question	% agreement	Action taken	Comments
A. Born before arrival (BBA)	64%	Change for COIN<br><br>Remove for international use	▪ BBA means that the baby was born in a vehicle (car, ambulance etc) or the roadside in Malawi which are considered dirty environments.<br>▪ Not included in WHO or COIN guidelines
**New proposed question**	**Indicate level of agreement**		**Your thoughts/comments**
Babies born en route to the hospital	1 2 3 4 5		

B. Prematurity (32-36/40 weeks gestation)

Expert responses

If the baby was delivered as a clean cold caesarean section for maternal reasons and the mother was not in labour then antibiotics should not be given. However, if the baby was born spontaneously premature then they should be given antibiotics

There was a study done recently in Tanzania, Professor Lindberg. Initially they had everyone on antibiotics and then they excluded the mothers who had caesarean sections for maternal reasons. They spoke about antibiotics being given to the mother and the fetus for 48 hours.

Gestation should also be given as weight.

Usually gestation is reported in months not weeks.

Gestation is often unknown. Every baby was treated as sepsis in Zambia. Every baby had a cannula on the neonatal unit which was a risk of infection alone. Too many babies were getting antibiotics. Every mother who had a GA their babies was admitted to the neonatal unit, a cannula sited and antibiotics were given. There was no thought process at all.

Gestation is not very useful, I only ever see 28 weeks, 32 weeks or term documented. Weight and gestation are more useful < 1.5 kg preterm, < 1kg very preterm. 1.5-2 kg could be growth restriction.

Gestation should be in combination with weight as they often don't know the gestation.

It is nice to have weight and gestation. Often prematurity is not recognised as it should be so weight is useful to highlight prematurity

You are better off using weight for gestation.

The simpler you make things the more effective they are. You are replying on ballard score which has +/- 2 weeks accuracy. So, a 31 weeker could fit into either category. I think you should just have prematurity just as a major risk factor.

Again, weight rather than gestation and low birth weight baby as a risk factor instead of prematurity and make it a major risk factor.

For peer review only

Agree for spontaneous but not for maternal reasons. Need to have weight and gestation/Ballard.

You need to have weight as well but they may be growth restricted and not premature but it is probably not a bad idea to have both premature and IUGR babies on antibiotics.

Add weight because sometimes the gestation is not known

I guess this is ok for one minor risk factor but I would also include weight to guide gestation.

I think my general comment would be the danger signs for WHO and the danger signs for essential care for every baby and essential care for small babies are the same. Those are probably the ones I would use to treat with antibiotics and not to treat with antibiotics.

Again, use weight. The weight is more useful than gestation. Being SGA is probably more important than premature.

**Original question**	**% agreement**	**Action taken**	**Comments**
B. Prematurity	71%	Change to include weight for gestation	▪ The application developers are trying to distinguish whether you are concerned with all premature babies being at risk of infection or just extreme (< 32/40) or slightly (32-26 weeks) preterm<br>▪ Not included in WHO or COIN guidelines
**New proposed question**	**Indicate level of agreement**		**Your thoughts/ comments**
<32/40 weeks gestation or <1500g	1 2 3 4 5		
32-36/40 weeks gestation or 1500-2500g	1 2 3 4 5		

C. Admitted with or history of fever – this question was originally asked as a MAJOR (DANGER) sign/symptom

Expert responses

I disagree. It needs to be repeated

It should be a minor risk factor

I agree. I would take it seriously if a mother reported that the baby had a fever because that is very unusual.

You need to say two temperatures or persistent.

There are so many mothers who over wrap their infants

For peer review only

I would monitor the baby. It should be a minor risk factor

I disagree. This depends on whether the baby is going be admitted for observation or not. Actually, I would read the reading myself because the rate of poor thermometers readings in developed countries is really high let alone in undeveloped countries so they would give every baby antibiotic. I am biased because I disagree with some of the WHOs stance on even the danger signs. I think they are too general, too many kids get antibiotics that do not need to. If you look at work done published in Rwanda, Kosovo and other countries, it's just that the antibiotic resistance rate is so high and kids are getting treatment that is unnecessary. So, I am going to lean probably most towards non-treatment than treatment.

I agree if this is the WHO or local guideline

**Original question**	**% agreement**	**Action taken**	**Comments**
C. Neonate admitted with or history of fever	57% agreement as a MAJOR risk factor	Change to 'other' risk factor and change wording	
**New proposed question**	**Indicate level of agreement**		**Your thoughts/comments**
Mother reports a non-measured fever	1 2 3 4 5		

2. Do you agree that the following is a DANGER/MAJOR sign or symptoms for neonatal sepsis?

Bilious vomiting

It could be a surgical reason and not sepsis.

In low resource settings yes we would start antibiotics

Definitely

Not alone.

It is a problem but I would not jump to sepsis.

It is a minor risk factor and it needs to be persistent to be major.

You are going to be over treating here because it could just be a congenital abnormality. It would be a minor risk factor.

It should be a minor risk factor and not stand alone.

That's not a danger sign but I agree that it's worth antibiotics but very rarely infectious. This kid is going to have one or two things wrong with it. The most likely thing is going to be a general obstruction. These babies in Africa are going to die. In a premature baby it is going is to be NEC and giving that baby antibiotics might be helpful. It's controversial.

For peer review only

Not in isolation. It should be a minor risk factor with abdominal distension

Distended abdomen

This is major risk factor in low resource settings as it usually frank fulminant NEC even with bigger babies. Surprised how common it was.

We do have to take it seriously. It is relatively subjective especially with preterm babies whose bellies always look big and round. I feel this needs 3 minors.

You again need to look for other reasons first. NEC is not as common in LIC.

If persistent

Very vague.

I disagree, you can get it from crying

This should be in combination with bilious vomiting and then this would be a major.

**Original question**	**% agreement**	**Action taken**	**Comments**
Bilious vomiting	50%	Change to combine with severe abdominal distension.	▪ Severe abdominal distension is a WHO sign of serious bacterial infection
**New proposed question**	**Indicate level of agreement**	**Please highlight whether it should be an 'other' risk factor or major (danger) sign**	**Your thoughts/comments**
Bilious vomiting with severe abdominal distension	1 2 3 4 5	Other or Major	

3. Do you agree with the following 'other' signs/symptoms of neonatal sepsis?

Irritability [*We acknowledge the comments suggesting the inclusion of "high pitched cry" but this is scored in a separate, meningitis algorithm*]

Expert responses

It is vague. More description is needed i.e. Inconsolable baby with an abnormal high-pitched cry.

It is not a sign of sepsis per say

This is subjective and needs to be defined further. A true irritable inconsolable baby I would agree. It needs videos.

I would probably do irritability and high-pitched cry

For peer review only

It could be something else.

This should be a major risk factor because the child could have meningitis

I disagree. Almost every baby in the 8-12 weeks of age is the most irritable thing in the world so I don't think so.

I would add lethargy and make this a major risk factor. I would try and simplify and not have all this major and minor and just have major. In practise this all gets too complicated and if there is something worrying you put them on antibiotics.

Original question	% agreement	Action taken	Comments
Irritability	64%	Change	▪ Not included in WHO or COIN guidelines
**New proposed question**	**Indicate level of agreement**		**Your thoughts/comments**
Irritable/ inconsolable baby	1 2 3 4 5		.

4. Do you think hypothermia < 35.5 °C should be added as a sign of neonatal sepsis in a low-income setting?

Expert responses

It is a major if persistent hypothermia despite warming

It is well recognised as a sign of sepsis. Suggested wording 'Hypothermia on two or more occasions.'

Yes, major. We know sepsis that can present as hypothermia or hyperthermia. It is just as important as a temperature of > 37.5 °C

Yes, minor. Hypothermia not responding to warming measures. Temperature instability is more useful than a specific high or low.

Yes, major but persistent hypothermia because I find many babies especially who are out born to be hypothermic.

Yes, major. Hypothermia despite efforts to warm. Hypothermia is a major problem. We have shown if you do not get your temperature up then your mortality is much higher. I would say this is very significant.

Yes, major. If it is persistent hypothermia despite normal supportive care.

Yes, major. What we have done for our definition of sepsis in the clinical trial that we are running is hypothermia < 35.5 °C after 1 hour of skin to skin. A lot of these babies are environmental, but I think the danger sign for sepsis is when you have a low temperature and you have had the best method of correcting, which is skin to skin contact and it is still low. You need to treat those for possible sepsis.

For peer review only

Yes, major. If it is persistent after addressing the environment of the baby it is a major risk factor

Yes, minor. Temperature instability is common with sepsis and might be a better term to use.

Yes, major. I am very familiar with helping babies to breathe and very familiar with the IMCI. I am not so familiar with COIN and some of this may be COIN specific. But with hypothermia it's more like the transient tachypnoea where if you are hypothermic after birth then that's something related to not drying or warming. However, if you are hypothermic days later that is less likely to be due to the environment. Most of the kids are not septic right away they take 24-40 hours to become septic especially for early onset sepsis. You tend to get that coming through mum's birth canal unless the mum's membrane has been ruptured for a long period of time. Then you may be totally asymptomatic so at first you may have a cold baby because they did not dry the baby that's one thing but there could be another baby that was totally not cold and then sporting a temperature of 35 °C because they became septic. Those are the two scenarios and there are environmental things added to it like if you are living in a cold area. Hypothermia would be more concerning to me later on than right after birth on admission to the neonatal unit. So, having new hypothermia, temperature instability or persistent hypothermia would all be concerning to me.

I personally don't think so but follow WHO.

**Original question**	**% agreement**	**Action taken**	**Comments**
Hypothermia <35.5°C	78%	Change	▪ This is a WHO and COIN danger sign. Experts agreed if hypothermia was persistent despite warming it should be included. However, the app is currently designed for just point of admission so we will only have a one-off temperature reading. Starting antibiotics and considering infection is covered in the hypothermia algorithm if the infant stays hypothermic despite attempts at rewarming.
**New proposed question**	**Indicate level of agreement**		**Your thoughts/comments**
Single measurement of temperature of <35.5°C	1 2 3 4 5		

5. Are there any additional risk factors, signs or symptoms that you think should be included for a low-income setting?

Expert responses

Particularly for preterm babies some of these signs are less specific such as reduced movement of limbs or hypotonia you expect that in preterm babies anyway. There are not

For peer review only - http://bmjopen.bmj.com/site/about/guidelines.xhtml

actually any good validated nosocomial scores for preterm babies while they are in hospital so the Rosenberg criteria is probably the best one but it still only has a sensitivity of 77%. It has 5 criteria which are apnoea, pallor, jaundice, hepatomegaly and seizures. What is difficult here is that you need a certain level of clinical skill to pick up the hepatomegaly. I am not saying they should be used here but it may be interesting to look at. Rosenberg RE, Ahmed AS, Saha SK, et al. Nosocomial sepsis risk score for preterm infants in low-resource settings. *J Trop Pediatr*. 2009;56(2):82–89.

Did the mother get any intrapartum antibiotics is probably too difficult to in cooperate as you never know what time etc

Poor capillary refill or perfusion

Hypothermia

Did the mother get any intrapartum antibiotics? Or did the mother get a GA? These factors affect whether the baby will come out hypotonic, tachypnoeic/shallow breathing and these babies are going to get antibiotics.

Eye symptoms. Red eye with pus. It would be a minor.

I don't think so. I would include all the danger signs from the WHO

Does the baby look ill?

**New proposed question**	***Rationale***	**Indicate level of agreement**	**Indicate whether it should be an 'other' risk factor or major (danger) sign**	**Your thoughts/comments**
A. Poor capillary refill or perfusion	*Expert suggestion*	1 2 3 4 5	Other or Major	
B. Unconscious	*WHO danger sign 'not moving when stimulated'*	1 2 3 4 5	Other or Major	
C. Swollen red eyelids with pus	*WHO localising sign of infection*	1 2 3 4 5	Other or Major	
D. Central cyanosis	*WHO danger sign*	1 2 3 4 5	Other or Major	
E. Does the baby look ill?	*Expert suggestion*	1 2 3 4 5	Other or Major	

6. Please comment on the criteria for 'consider meningitis'

Expert responses

I prefer unstable temperature above 38 degrees or less than 36.5 degrees Celsius.

For peer review only

Bulging fontanelle yes, hypotonia yes but would also include hypertonia, floppiness, absent suck.

Remove 37 centimetres because it does not apply to a premature baby and just have obvious enlarging head.

It should be hypertonia or hypotonia. Irritability, lethargy is fine. Hydrocephalus is a very late sign of meningitis so not helpful acutely so should be removed. Convulsions need to be described as abnormal repetitive stereotypical movement.

Yes. That is fine

Hydrocephalus is rubbish. You would not get a head circumference.

I would disagree with the hydrocephalus bit as there could be an anatomical reason for their hydrocephalus.

You don't usually get hydrocephalus very early on in meningitis. This one seems a bit odd to me. I would get rid of it. You can make the diagnosis with bulging fontanelle and everything else.

I have to say if you have hydrocephalus in the acute setting the symptoms or signs of hydrocephalus would be more important than the head circumference.

Add in high pitched cry/convulsive cry and abnormal movements (+/- convulsions). Often baby's seizures are very subtle, it may be abnormal movements of the lips, or slightly abnormal movement of the legs which people don't class as convulsions. Remove lethargy because it is non-specific. A febrile baby who is lethargic does not specifically point to meningitis.

Remove hypotonia it is too complex for nurses to assess for routine care and it should be kept simple.

Remove hydrocephalus it is a post meningitis event and they don't present with it

Overall recommendation: Persistent temperature > 37.5, bulging fontanelle, persistent irritability with a convulsive cry, abnormal movements +/- convulsions

I would not have convulsions alone to diagnose meningitis as it could be birth asphyxia, I would want to see it with a temperature. I would remove hydrocephalus as it could be an anatomical cause.

I would add hypertonia/hypotonia

Agree with all of them

Happy with these.

I am guessing the only reason for diagnosing meningitis is for antibiotic duration.

I would say they need to have a temperature over 38 °C not 37.5 °C. You are going to have a lot of kids here with temperatures of 37.5 °C which seems low. Bulging fontanelle with a temperature of 38 °C seems reasonable. Hypertonia is a tough one to diagnose. A kid who had an in-utero stroke is going to be hypertonic. Do these kids get antibiotics every time they have a fever?

Acute seizures are straight forward. I like the way WHO does it and I do not normally say this because if the baby has convulsions acutely you treat. What if your kid has a seizure

For peer review only

disorder? You would have to give antibiotics constantly which is not going to treat anything because he has a seizure disorder. I would say it has to be acute seizures. Hydrocephalus, new onset? Baby? They pick standard deviations on the head circumference which is probably appropriate for a concern but then you are still treating 2 out of every 100 kids because of they just have a big head. I would say just go with the danger signs of fever, lethargy and convulsions which will give you a secure diagnosis and the rest are not.

I would not include hydrocephalus. Hydrocephalus is a separate issue and a whole different diagnosis

**Original question**	**% agreement**	**Action taken**
Temp >37.5 °C AND any of<br>• Bulging Fontanelle<br>• Hypertonia<br>• Persistent irritability<br>• Lethargy<br>OR<br>• Convulsions/History of convulsions<br>• Hydrocephalus	28% agreement	Change
**New proposed question**	**Indicate level of agreement**	**Your thoughts/comments**
Bulging fontanelle	1 2 3 4 5	
Irritability with a high-pitched cry	1 2 3 4 5	
Drowsy, lethargic or unconscious	1 2 3 4 5	
Abnormal movements/twitching or convulsions with temp >37.5	1 2 3 4 5	
Abnormal tone with temp >37.5	1 2 3 4 5	

For peer review only

Section 2. Hypoxic Ischaemic Encephalopathy

1. Do you agree with the following risk factors for hypoxic ischaemic encephalopathy?

From expert feedback the term 'birth asphyxia,' has been changed to hypoxic ischaemic encephalopathy (HIE). In addition, NeoTree will no longer be using risk factors to aid making a diagnosis of HIE and it will be adjusted to use the already validated Thompson score. However, the risk factors that were agreed upon by experts in the first round will be a prompt on the app for the user to perform the Thompson score.

Birth injury

Expert responses

What do you mean by birth injury it needs to be defined.

As a screening tool it is a valid important thing to think about.

I disagree. Meaning what? This is really ambiguous and needs a description. It could be a facial laceration and that would not give you asphyxia.

I disagree. Need to define further. I would not consider a bruise or a cephalohematoma as an injury. It needs to be specific. However, I would still only consider as there are far more important risk factors which would be more predictive for the infant.

It depends on what the birth injury is. If they come out with a very moulded head, large cephalohematoma, fractured clavicle, erb's palsy then I would assume they have had significant problems extracting. However, something like a laceration I would not consider as a significant birth injury. You need to specify the birth injuries.

I disagree. What are we talking about when we say birth injury here? More detail is needed. Broken bones or significant neurological deficit/nerve injury I would agree with.

You need to be more specific i.e. a fracture or nerve palsy.

Disagree. Too vague. I would not include this one.

This needs to be defined.

Disagree. These need to be defined because it is a very broad term. If there are palsies, cephalohematoma or subgaleal, fractures, extensive bruising then I would agree.

Disagree. For instance, bruising does not normally cause asphyxia.

**Original question**	**% agreement**	**Action taken**	**Comments**
Birth injury as risk factor for HIE	29%	Change	▪ Not included in WHO or COIN guidelines<br>▪ App developer is keen to see if we specify the birth injuries whether this can be included
**How should we describe "significant" birth injury?**			

For peer review only

2. Do you agree with the following signs/symptoms for hypoxic ischaemic encephalopathy?

Absent suck and gestation > 34/40

Expert responses

Disagree. This is not sensitive or specific. It could be because you are septic. I really worry that the approach to this is not scientific and it is not based on existing evidence base. WHO and COIN will have followed a formal process to what is included or excluded. I worry about any of the signs and symptoms that the app developers have suggested that are not evidence based to make a recommendation. Research needs to be done for any suggestions of the app developers and recommendations cannot be made until you have the evidence.

Disagree. I think it should be 35 weeks and this really depends on how old the neonate is. This assessment needs to be at least 1 hour of age as the infant needs some transition time.

Disagree. I would have the gestation at 32/33 weeks and then I would agree. For the rest I agree but my comment around these questions is that if you are measuring these signs and symptoms too early then you can over diagnose. Often babies that have a lactic acidosis very early on but may not have a significant amount of hypoxia may be obtunded but then recover very quickly as oppose to a baby that has had a significant hypoxic hit and you need to wait at least an hour.

Disagree. I think the only reliable clinical signs are seizures, coma, flaccid and very poor tone. I would really use the Sarnat score or the Thompson criteria here. However, it is difficult to assess for HIE using the Sarnat score for preterm babies as it has mainly been designed for term babies. The Thompson score which I think was designed in East Africa has a much more pragmatic approach and I would use that to guide here.

Disagree. Just use the sarnat stages.

**Original question**	**% agreement**	**Action taken**	**Comments**
Absent suck and gestation >34/40 weeks	57%	Change but include as part of the Thompson score	
**New proposed question**	**Indicate level of agreement**		**Your thoughts/comments**
Absent suck and gestation >32/40 weeks	1 2 3 4 5		

For peer review only

Section 3. Respiratory distress of the newborn (RDN)

1. Do you agree with the diagnostic criteria for Respiratory Distress of the Newborn (RDN):

From expert feedback the NeoTree application will no longer generate a respiratory diagnosis (respiratory distress syndrome, meconium aspiration, transient tachypnoea of the newborn) and it will just guide you to treating the symptoms. However, the above possible diagnoses will still be included as teaching points. NeoTree allows for multiple diagnoses i.e the preterm baby can have a diagnosis of RDS and pneumonia.

A. Respiratory distress syndrome

Expert responses

Lower gestation to 34-35 weeks. Older than that they are not at risk.

Agree. It's reasonable. You have to both

Or diabetic mother

Yes agree. Include weight for suggested gestation.

I would say a lower gestation. The risk for a 36 weeker is relatively low. I would change gestation to weight and say < 2 kg.

1 disagree use weight < 2000g

I would say less than 34 weeks

Disagree in making a diagnosis. Just treat the symptoms

**Original question**	**% agreement**	**Action taken**	**Comments**
Respiratory Distress Syndrome	50%	Change	
**New proposed question**	**Indicate level of agreement**		**Your thoughts/comments**
Gestation <34/40 or <2000g	1 2 3 4 5		

B. Congenital pneumonia

Expert responses

I would change temperature to more than 38 °C or less than 36.5.

I would say hypothermia or hyperthermia and if you mention temperature you have to say both.

Not so sure I agree with foul smelling liquor

I would remove unilateral signs as pneumonia can be bilateral so just say coarse crackles.

I think the temperature should be more than 38 °C.

For peer review only

Disagree. Again, in my opinion, I would treat the symptoms and do not worry about diagnosing. For example, for congenital pneumonia, at CDC we had a whole panel of all the experts trying to agree on how to diagnose pneumonia. The decision of the panel was that it is impossible in a developed country to diagnose neonatal pneumonia. So, if you can't do it here how do you expect them to do it there. The temperature should also be more than 38 °C.

Again, I don't think you need risk factors. You are going to diagnose it even if there are no risk factors but it's fine.

**Original question**	**% agreement**	**Action taken**	**Comments**
Congenital Pneumonia	50%	Change	
**New proposed question**	**Indicate level of agreement**		**Your thoughts/comments**
Temp >37.5ºC or <36.5ºC	1 2 3 4 5		
Coarse crackles (instead of unilateral signs)	1 2 3 4 5		

For peer review only

ORIGINAL
Sepsis algorithm

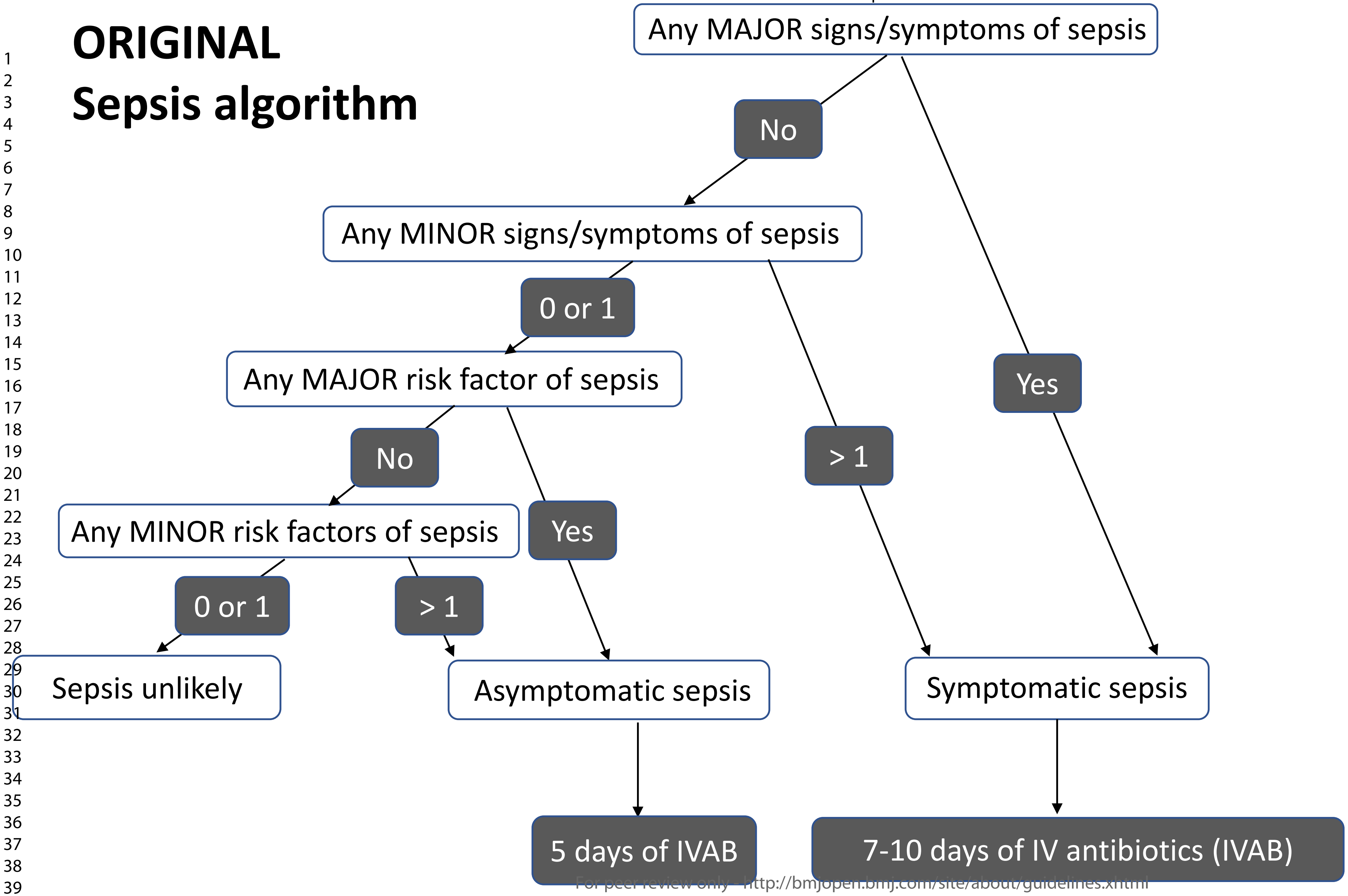

REVISED Sepsis algorithm

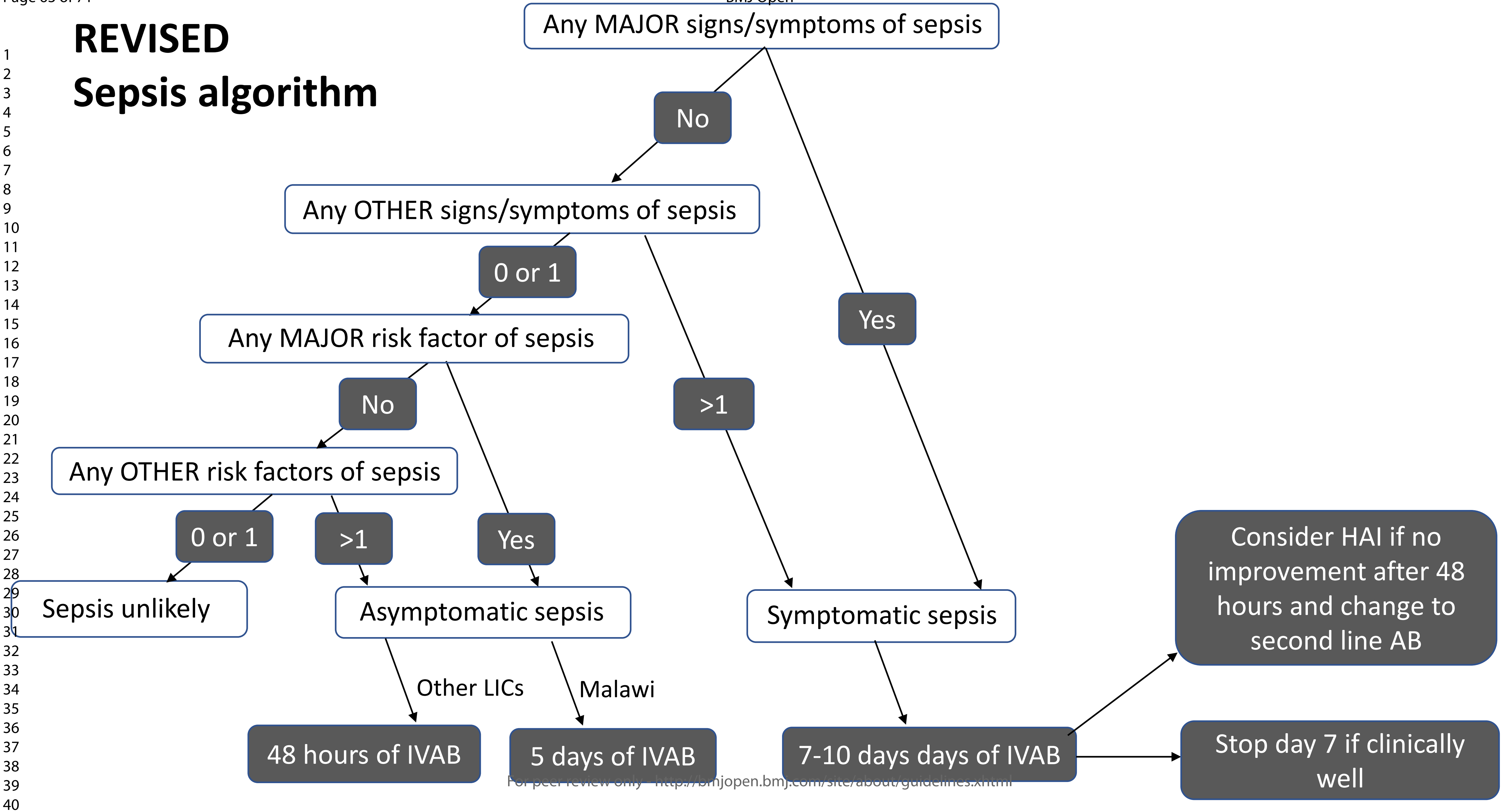

For peer review only - http://bmjopen.bmj.com/site/about/guidelines.xhtml

Revised neonatal sepsis risk factors, signs and symptoms

Risk factors

Major

- Maternal fever >38˚C in labour
- Prolonged rupture of membranes (PROM) >18 hours
- Foul smelling amniotic fluid

Other risk factors

- <32/40 weeks gestation or <1500g (spontaneous labour only)

Signs/symptoms

Major

- Neonatal temperature >37.5 ˚C
- Boil/abscess
- Grunting/severe respiratory distress/ mod-severe WOB
- Lethargy
- Umbilical redness extending to the periumbilical skin or umbilicus draining pus
- Deep jaundice: palms and soles of the baby deep yellow
- Tachypnoea > 60 bpm
- Convulsions/twitching or abnormal movements
- Many or severe skin pustules
- Bilious vomiting with severe abdominal distension
- Bulging fontanelle
- New onset of poor feeding
- Not moving when stimulated
- Swollen red eyelids with pus
- Central cyanosis

Other signs/symptoms

- Pallor

Revised neonatal sepsis management

Neonatal sepsis management

Investigations

- *If possible* perform sepsis screen: FBC, CRP, blood culture
- LP only for those who have clinical signs of meningitis (but stable) or late-onset neonatal sepsis

Antibiotic choices

- 1st line: give local recommendations
- IM/IV gentamicin and benzylpenicillin **or** ampicillin
- 2nd line: If no improvement after 48 hours change to a third-generation cephalosporin
- IV cloxacillin and gentamicin if greater risk of staphylococcal (skin infection).

Antibiotic duration (without investigations)

Asymptomatic sepsis: Treat for 48 hours and stop if well (Treat for 5 days in Malawi)

Symptomatic sepsis: Stop at day 7 if clinically well otherwise continue for 10 days

Antibiotic doses

Gentamicin (IM/IV)

<7 days: 3 mg/kg (LBW) and 5 mg/kg (normal BW) per dose once a day

>7 days 7.5 mg/kg once a day

Ampicillin (IM/IV)

<7 days: 50 mg/kg every 12 h

>7 days every 8 h

Benzylpenicillin (penicillin G) (IM)

<7 days: 50 000 U/kg every 12 h

>7 days every 6 h

Supportive care

- Thermoregulation: aim for 36.5-37.5 °C
- Provide respiratory support oxygen or CPAP as needed according to CPAP algorithm
- Check blood glucose and provide feeding support as needed
- Consider 10ml/kg fluid bolus if shocked as per SHOCK algorithm
- Consider 2.5mls/kg 10% dextrose if BS<2.5 mmol or 45 mg/dl – as per HYPOGLYCAEMIA algorithm

For peer review only - http://bmjopen.bmj.com/site/about/guidelines.xhtml

ORIGINAL
Birth Asphyxia algorithm

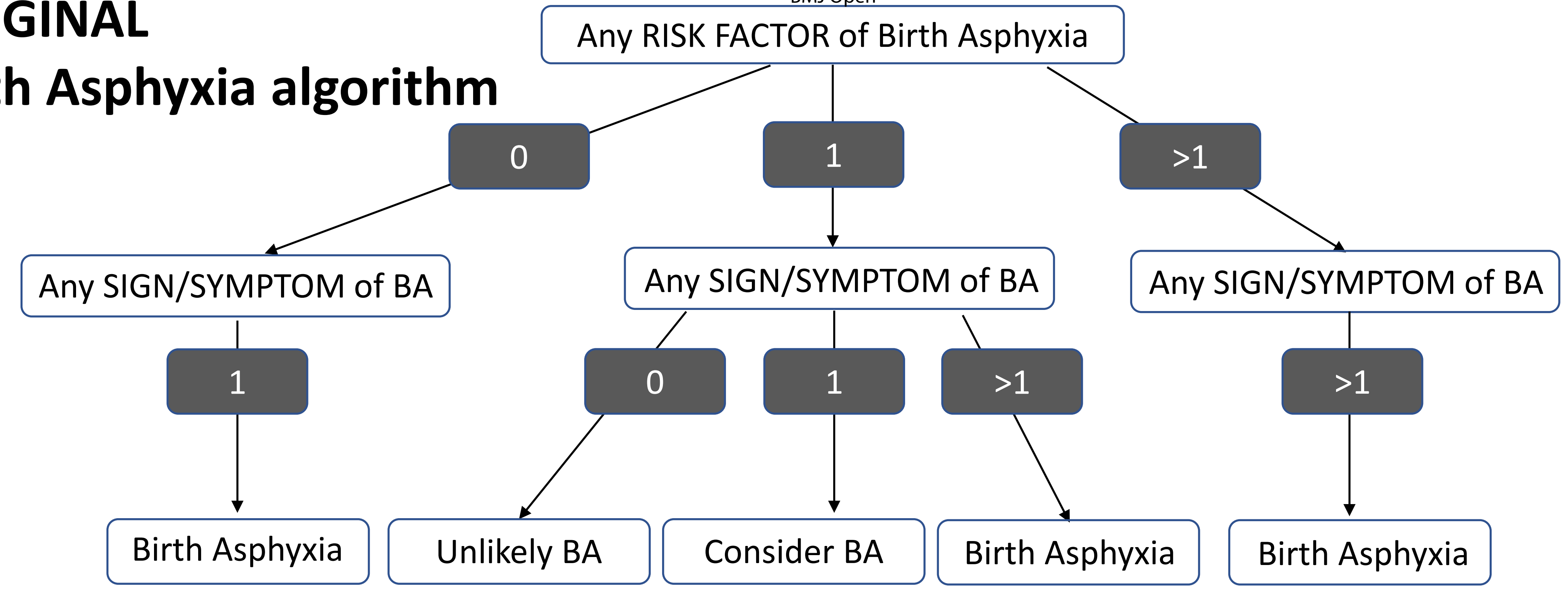

Signs/symptoms

- Coma
- Convulsions
- Lethargy
- Irritable
- Hypotonia AND >34/40 gestation
- Absent suck AND >34/40 gestation

Risk factors

- Foetal distress
- Prolonged second stage
- Delivery = Vacuum/Emergency Caesarian section/Breech
- 5 minute Apgar <7
- Resuscitation: Bag valve mask >5 mins/CPR/resuscitation >10 mins
- Birth Injury

For peer review only - http://bmjopen.bmj.com/site/about/guidelines.xhtml

REVISED Hypoxic Ischaemic Encephalopathy algorithm

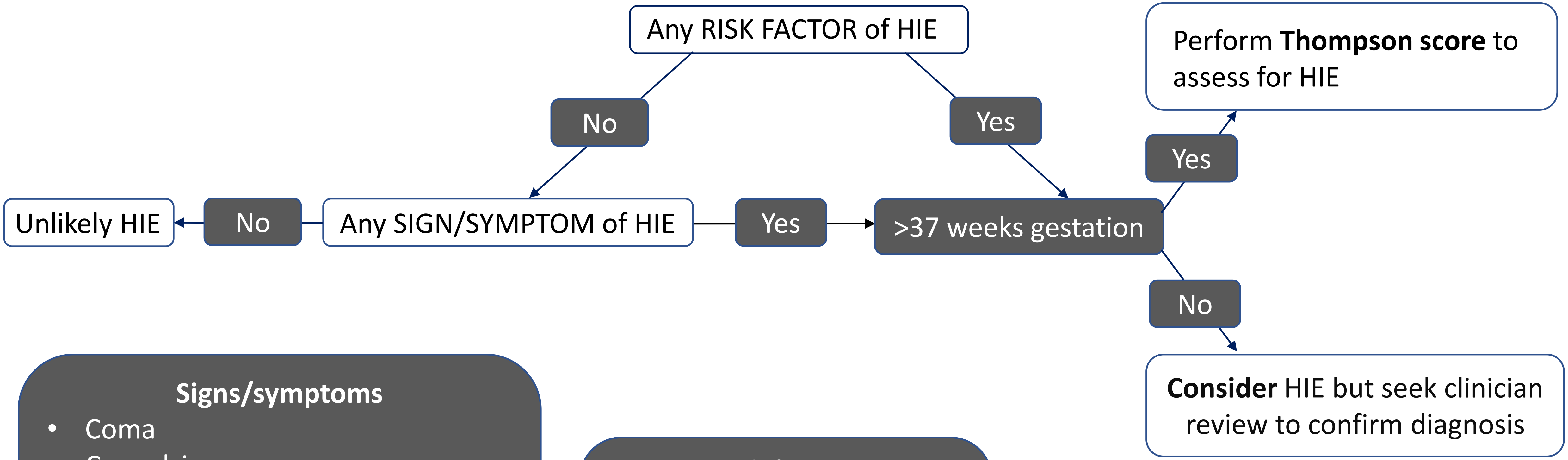

Signs/symptoms

- Coma
- Convulsions
- Lethargy
- Hypotonia
- Absent Moro reflex
- Absent suck
- Respiratory distress

Risk factors

- Foetal distress
- Resuscitation: Assisted ventilation >5 minutes or CPR >10 minutes
- Apgar at 5 minutes <7

For peer review only - http://bmjopen.bmj.com/site/about/guidelines.xhtml

Revised Hypoxic Ischaemic Encephalopathy management

HIE management

Thermoregulation
Aim for normothermia
Avoid hyperthermia and hypothermia

Respiratory support
Give respiratory support as appropriate. Give oxygen if saturations <90% in air

Feeding support, anticonvulsants and antibiotics
Give feeding support as appropriate

1. Oral feeds (breast feed or bottle if breastfeeding is not possible)
2. Give NG feeds if BS <2.5mmol/L or <45mg/dL and not tolerating oral feeds
3. Give IV fluids if BS <2.5mmol/L or <45mg/dL and not tolerating NG feed

If evidence of seizure activity give Phenobarbitone as per convulsions algorithm.
Reassess suck, tone, coma, seizures every 8 hours for 72 hours in hospital
Treat for sepsis with antibiotics

For peer review only - http://bmjopen.bmj.com/site/about/guidelines.xhtml

ORIGINAL RDN algorithm

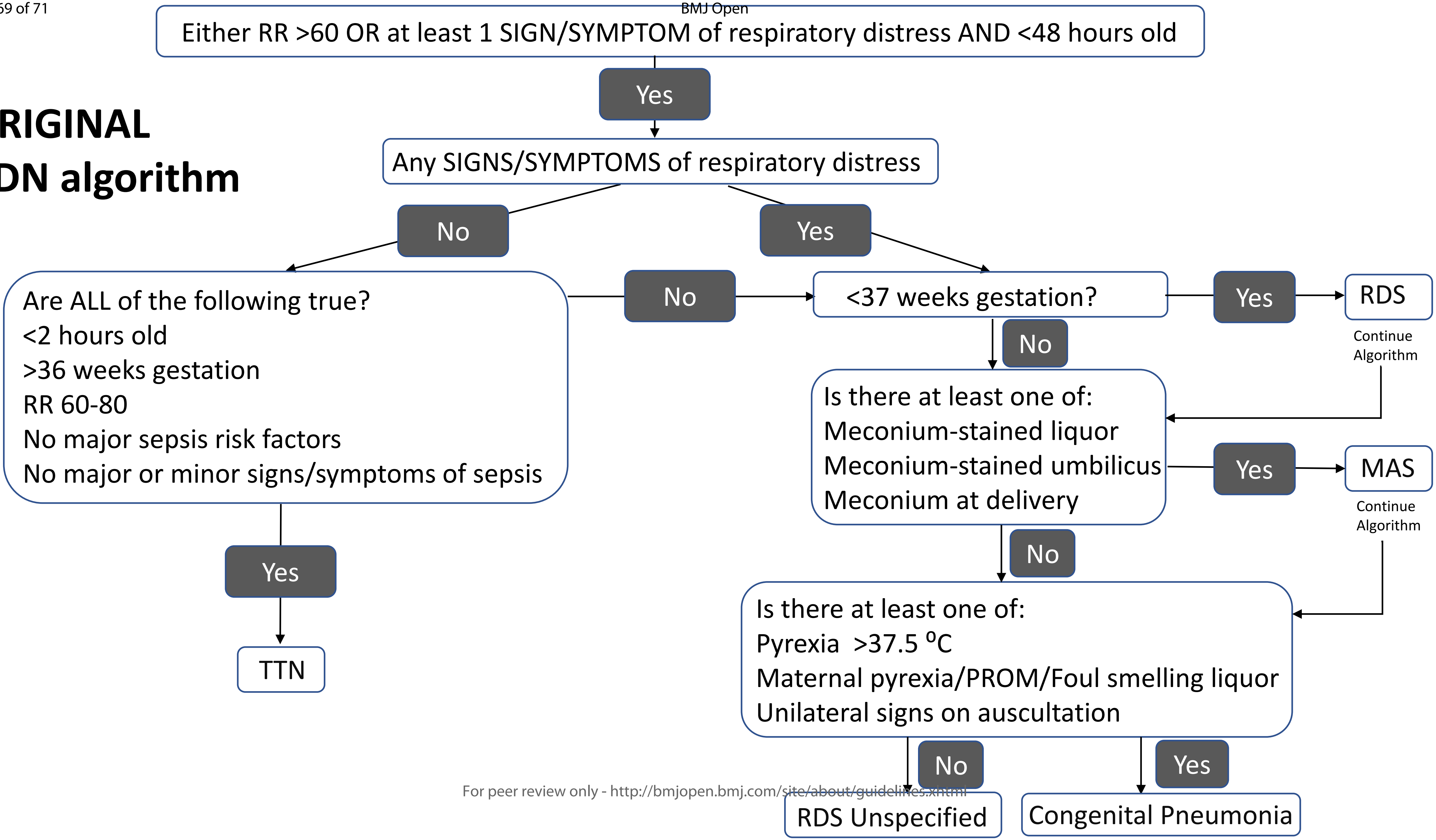

REVISED
RDN management

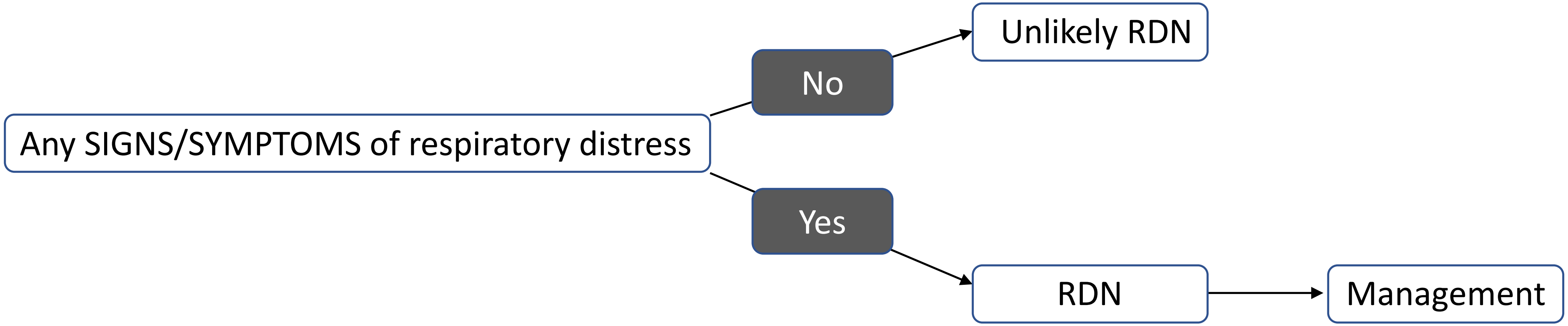

Signs/symptoms of respiratory distress

- Oxygen saturations in air <90%
- Tachypnoea >60 breaths per minute
- Fever >37.5ºC or <36ºC and crepitations on auscultation
- Grunting
- Recessions
- Any increased work of breathing
- Cyanosis
- Apnoea

For peer review only - http://bmjopen.bmj.com/site/about/guidelines.xhtml

Revised Respiratory Distress of the Newborn management

Respiratory distress of the newborn management

Investigations

Perform a chest x-ray if it will change management or if the infant is not improving as expected or deteriorating.

Airway and respiratory support

Position airway

Give oxygen if oxygen saturations < 90% in air

If > 1kg consider CPAP according to CPAP algorithm

Feeding support

If breathing 60-80 bpm use cup/NGT

If needing CPAP use OGT

If breathing > 80bpm consider IV fluids

IV Access for Antibiotics

Give antibiotics including for suspected TTN (unless have chest x-ray and can safely exclude.)

Teaching points

Meconium aspiration

- Consider if meconium stained amniotic fluid
- Just because there was meconium at delivery doesn't mean it was aspirated.
- Most aspiration occurs in utero before the baby is born, so more likely if there was foetal distress in labour or has signs of HIE
- Floppy babies with severe HIE and meconium aspiration are unlikely to benefit from CPAP.

Transient tachypnoea of the newborn (TTN)

- These babies are not unwell
- RR is usually between 60-80 bpm and they don't usually need oxygen
- This should resolve in the first few hours of life.
- TTN is more likely following elective C-section or a rapid vaginal delivery.
- Give antibiotics if unable to exclude other causes i.e no chest xray

Respiratory distress syndrome

- Consider if < 37 weeks or diabetic mother
- Benefit from CPAP see Algorithm

Congenital Pneumonia

- Consider if temperature > 37.5 ^{o} c or < 36 ^{o} c and crepitations on auscultation
- Risk factors for sepsis i.e maternal fever/PROM/foul smelling amniotic fluid

For peer review only - http://bmjopen.bmj.com/site/about/guidelines.xhtml

Hypothermia Algorithm and management

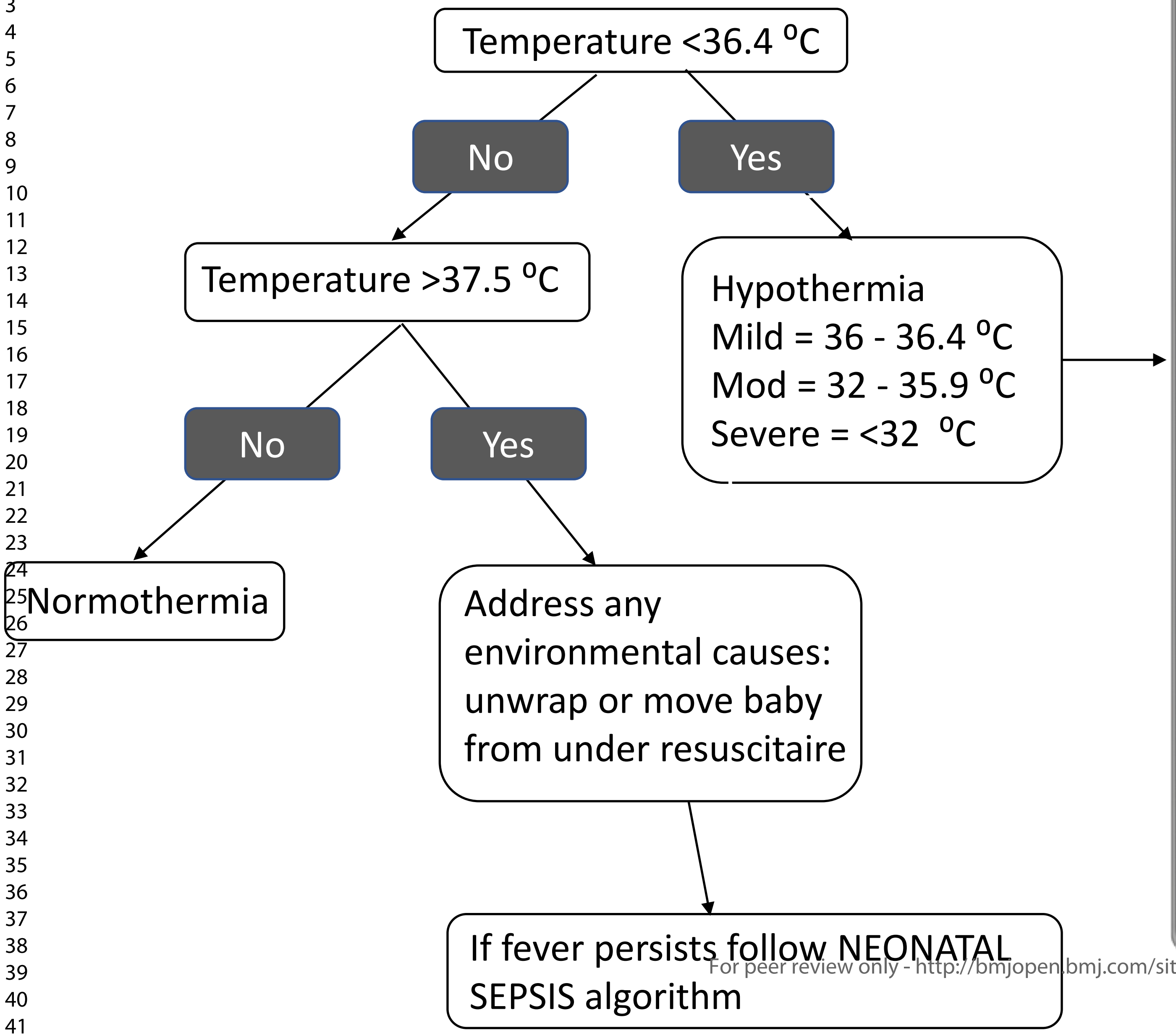

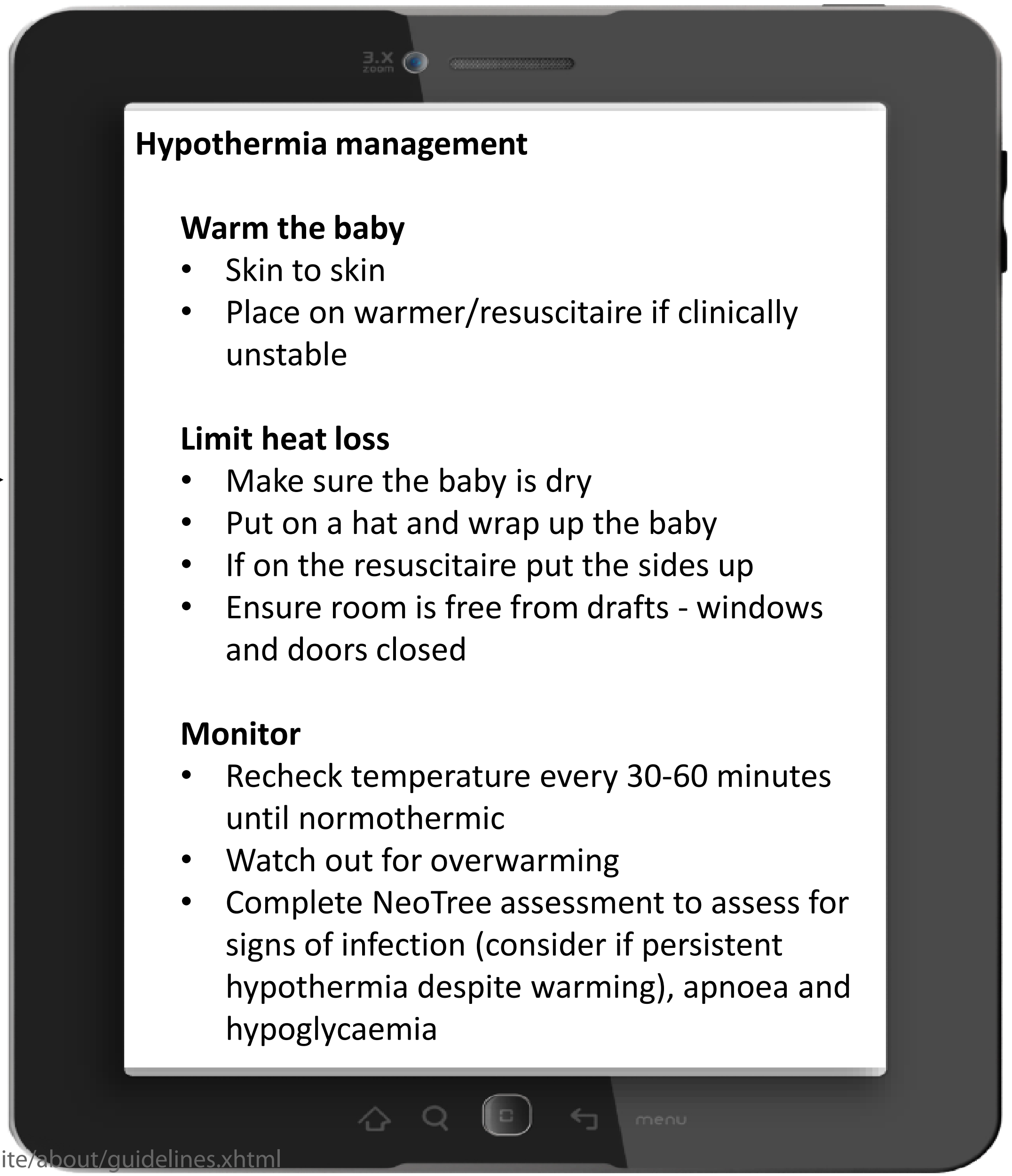

For peer review only - http://bmjopen.bmj.com/site/about/guidelines.xhtml
